# The pattern of apolipoprotein A-I lysine carbamylation reflects its lipidation state and the chemical environment within human atherosclerotic aorta

**DOI:** 10.1016/j.jbc.2022.101832

**Published:** 2022-03-15

**Authors:** Shawna Battle, Valentin Gogonea, Belinda Willard, Zeneng Wang, Xiaoming Fu, Ying Huang, Linda M. Graham, Scott J. Cameron, Joseph A. DiDonato, John W. Crabb, Stanley L. Hazen

**Affiliations:** 1 Department of Cardiovascular & Metabolic Sciences, Cleveland Clinic, Cleveland, Ohio, USA; 2 Cleveland Clinic Lerner College of Medicine, Case Western Reserve University, Cleveland, Ohio, USA; 3 Department of Chemistry, Cleveland State University, Cleveland, Ohio, USA; 4 Proteomics Shared Laboratory Resource, Cleveland Clinic, Cleveland, Ohio, USA; 5 Department of Biomedical Engineering, Lerner Research Institute, Cleveland Clinic, Cleveland, Ohio, USA; 6 Heart Vascular and Thoracic Institute, Cleveland Clinic, Cleveland, Ohio, USA; 7 Taussig Cancer Center, Cleveland Clinic, Cleveland, Ohio, USA; 8 Cole Eye Institute, Cleveland Clinic, Cleveland, Ohio, USA

**Keywords:** apolipoprotein A-I, high-density lipoprotein, cardiovascular disease, carbamylation, oxidation, homocitrulline, cyanate, myeloperoxidase, urea, apoA-I, apolipoprotein A-I, CID, collision-induced dissociation, CNO^−^, cyanate ion, CVD, cardiovascular disease, DSH, Double SuperHelix, DSSO, disuccinimidyl sulfoxide, DTPA, diethylenetriamine pentaacetic acid, HCit, homocitrulline, HDL, high-density lipoprotein, mAb, monoclonal antibody, MPO, myeloperoxidase, MS, mass spectrometry, PTM, post-translational modification, rh-apoA-I, recombinant human apoA-I, rHDL, reconstituted HDL, SCN^−^, thiocyanate ion, SPR, surface plasmon resonance

## Abstract

Protein lysine carbamylation is an irreversible post-translational modification resulting in generation of homocitrulline (*N*-ε-carbamyllysine), which no longer possesses a charged ε-amino moiety. Two distinct pathways can promote protein carbamylation. One results from urea decomposition, forming an equilibrium mixture of cyanate (CNO^−^) and the reactive electrophile isocyanate. The second pathway involves myeloperoxidase (MPO)-catalyzed oxidation of thiocyanate (SCN^−^), yielding CNO^−^ and isocyanate. Apolipoprotein A-I (apoA-I), the major protein constituent of high-density lipoprotein (HDL), is a known target for MPO-catalyzed modification *in vivo*, converting the cardioprotective lipoprotein into a proatherogenic and proapoptotic one. We hypothesized that monitoring site-specific carbamylation patterns of apoA-I recovered from human atherosclerotic aorta could provide insights into the chemical environment within the artery wall. To test this, we first mapped carbamyllysine obtained from *in vitro* carbamylation of apoA-I by both the urea-driven (nonenzymatic) and inflammatory-driven (enzymatic) pathways in lipid-poor and lipidated apoA-I (reconstituted HDL). Our results suggest that lysine residues within proximity of the known MPO-binding sites on HDL are preferentially targeted by the enzymatic (MPO) carbamylation pathway, whereas the nonenzymatic pathway leads to nearly uniform distribution of carbamylated lysine residues along the apoA-I polypeptide chain. Quantitative proteomic analyses of apoA-I from human aortic atheroma identified 16 of the 21 lysine residues as carbamylated and suggested that the majority of apoA-I carbamylation *in vivo* occurs on “lipid-poor” apoA-I forms *via* the nonenzymatic CNO^−^ pathway. Monitoring patterns of apoA-I carbamylation recovered from arterial tissues can provide insights into both apoA-I structure and the chemical environment within human atheroma.

High-density lipoprotein (HDL) is a heterogeneous particle varying in composition of protein, lipids, size, density, and electrophoretic mobility (1, 2). The major protein constituent of HDL is apolipoprotein A-I (apoA-I), comprising approximately 70% of the total protein found in the lipoprotein particle (3). Historically, HDL has been thought to be protective based on its inverse association with cardiovascular disease (CVD) and ability to promote reverse cholesterol transport, anti-inflammatory, antiapoptotic, and more recently, anticancer effects *in vitro* and *in vivo* (4, 5, 6, 7, 8, 9). Studies have shown that at sites of chronic inflammation and within atherosclerotic lesions, apoA-I/HDL is selectively targeted for various post-translational modifications (PTMs), rendering it dysfunctional (10, 11, 12, 13, 14). Such modifications may occur through various mechanisms including but not limited to carbamylation, chlorination, oxidation, glycation, nitration, and methylation (14, 15, 16, 17, 18, 19).

Carbamylation is an irreversible PTM that occurs at sites of inflammation (*e.g.*, atherosclerotic plaque) and promotes proatherosclerotic phenotypes; moreover, protein carbamylation also serves as an independent risk factor for coronary artery disease and future adverse cardiac events, including myocardial infarction, stroke, and death (20). Classically, protein carbamylation has long been known to occur in the presence of urea (Fig. 1) (21, 22). Urea slowly decomposes into cyanate (CNO^−^), which readily isomerizes into the reactive electrophile isocyanate. Isocyanate can rapidly carbamylate the ε-amino moiety of lysine residues in proteins forming carbamyllysine, also known as homocitrulline (HCit) (22). For example, protein carbamylation is known to occur during renal dysfunction, when urea levels are elevated, altering lipoprotein function (23, 24). A recently described second mechanism for protein carbamylation uses the leukocyte-derived enzyme myeloperoxidase (MPO), a heme protein abundantly found in granules of neutrophils and monocytes (Fig. 1) (20, 25). Leukocytes release MPO at sites of inflammation, and MPO is enriched within human atherosclerotic lesions (20, 25). MPO has been shown to foster protein oxidative modifications within diseased human arterial tissues (26, 27, 28, 29, 30, 31, 32, 33, 34, 35). In the presence of physiological levels of thiocyanate (SCN^−^), which is derived predominantly from both diet and smoking (20, 36), we showed that MPO can both generate CNO^−^ and catalyze protein carbamylation *in vivo* at sites of inflammation and vascular disease (20, 36). Moreover, smoking is a major risk factor for aortic atherosclerotic diseases, and prior studies highlight a link between MPO, smoking, atherosclerosis, and protein carbamylation (20, 37, 38). Several subsequent studies have further shown that MPO-catalyzed carbamylation of proteins can alter their function, including transformation of lipoproteins (both HDL and low-density lipoprotein) into dysfunctional forms (20, 23, 39, 40).

**Figure 1:**
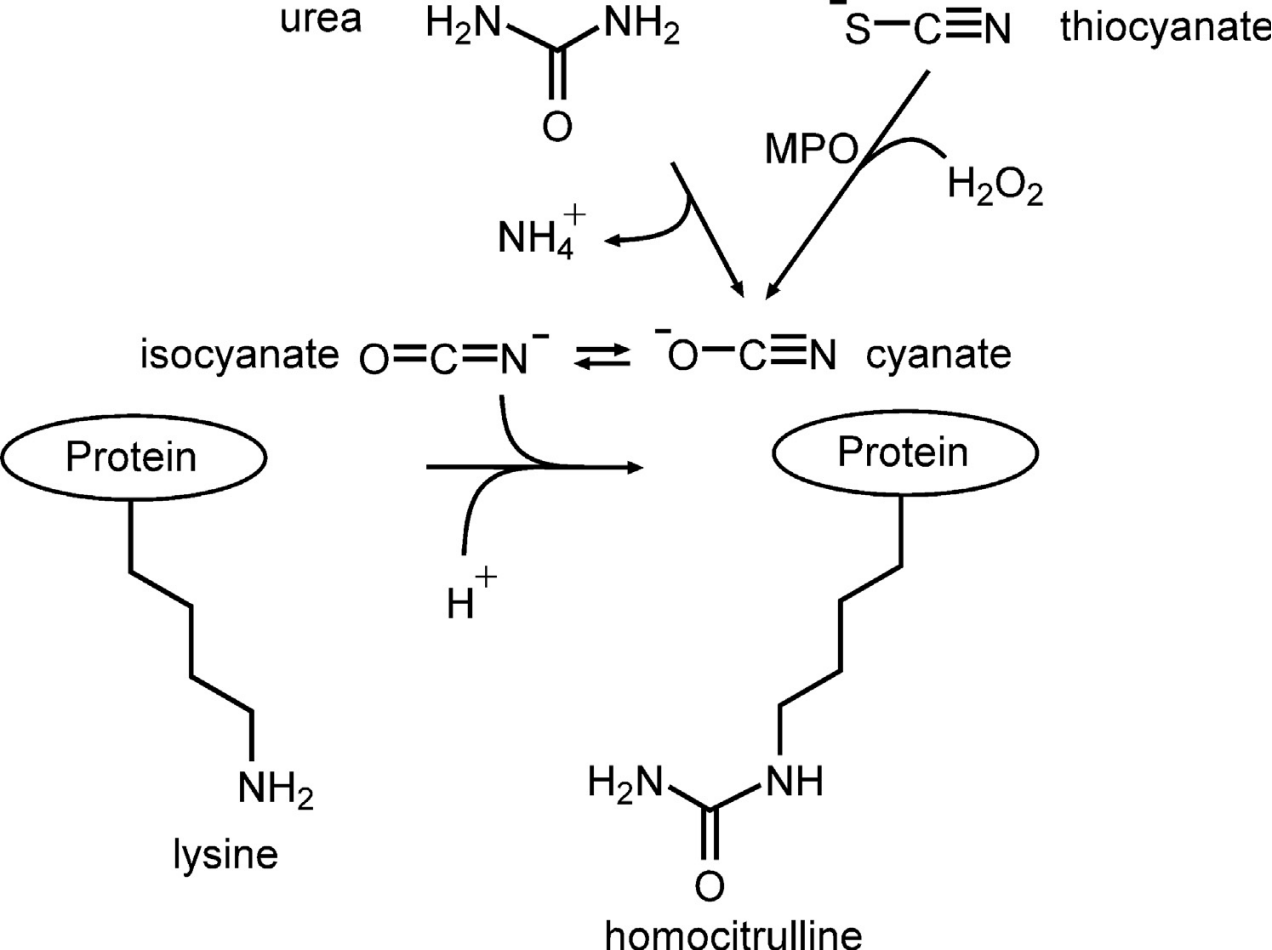
**Enzymatic and inorganic protein carbamylation pathways.** The inorganic protein carbamylation pathway is initiated by urea degradation into an equilibrium mixture of cyanate and isocyanate. Isocyanate promotes nucleophilic attack of lysine residues leading to protein-bound homocitrulline generation. Protein-bound homocitrulline can also be produced by the enzymatic pathway, which involves myeloperoxidase (MPO)-catalyzed oxidation of thiocyanate to form cyanate/isocyanate.

In this study, we first sought to demonstrate whether apoA-I recovered from human aorta is targeted for protein carbamylation within human atherosclerotic tissues. Then, by quantitatively mapping the sites of apoA-I carbamylation in model systems *in vitro* for comparisons with apoA-I recovered from human atherosclerotic aorta, we sought to determine if the site-specific pattern of apoA-I lysine carbamylation can serve as a probe yielding insights into the environment within human atherosclerotic aorta. We specifically asked (i) within aorta, are lipid-poor *versus* HDL-associated forms of apoA-I both carbamylated and (ii) does apoA-I carbamylation occur preferentially by the MPO-H_2_O_2_-SCN^−^ pathway (hereafter called “the MPO system”) or the urea-associated cyanate (CNO^−^) system. Finally, by examining the hierarchy of apoA-I lysine residue carbamylation in reconstituted HDL (rHDL) by the MPO system *in vitro*, we also sought to examine whether carbamylation patterns could serve as a probe to gain insights into the structure of rHDL.

## Results

### The HCit content of apoA-I recovered from human atherosclerotic lesions is significantly higher than that of plasma total protein-bound HCit

**Figure 2:**
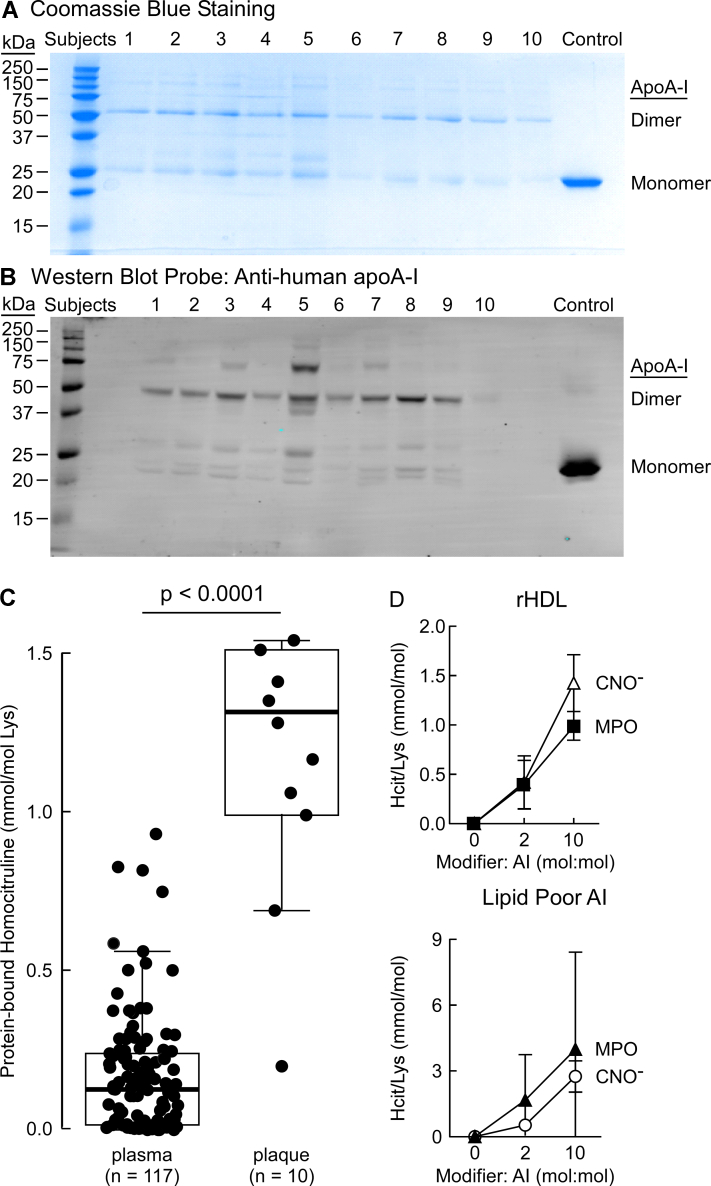
**Characterization of apoA-I isolated from human lesions.***A*, reducing SDS-PAGE gel of apoA-I immunoprecipitated from human atherosclerotic lesions collected from 10 subjects. Bands for the apoA-I monomer and dimer in all samples are indicated (lanes 1–10). *Last lane* shows the monomeric recombinant apoA-I as control. B, Western Blot analysis of apoA-I isolated from human lesions and probed using “in house” 10G1.5 anti-apoA-I-specific antibody. *C*, LC–MS/MS quantification of homocitrulline (HCit) in plasma apoA-I (117 subjects) and isolated from human lesions (10 subjects). *p* Value shown was determined using Wilcoxon rank sum test. *D*, LC–MS/MS quantification of HCit in *in vitro*-modified apoA-I in response to increasing moles of CNO^−^ or hydrogen peroxide (H_2_O_2_) to moles of apoA-I. *Top panel* in *D*, dose–response curve using >0.05 mg/ml rHDL carbamylated using increasing moles of H_2_O_2_ or CNO^−^: moles of apoA-I. *Bottom panel*, titration curve using >0.05 mg/ml lipid-poor apoA-I carbamylated using increasing moles of H_2_O_2_ or CNO^−^: moles of apoA-I. apoA-I, apolipoprotein A-I; CNO^−^, cyanate ion; rHDL, reconstituted high-density lipoprotein.

In initial studies, we sought to examine the extent of protein-bound lysine carbamylation on apoA-I recovered from human atherosclerotic plaque. In view of the fact that we have shown commercially available antibodies for apoA-I have bias for recognition of different oxidized apoA-I forms (41), established immune-isolation methods were used to quantitatively recover total apoA-I from human atherosclerotic tissues (see the Experimental procedures section, (41)). Figure 2*A* shows a Coomassie blue–stained gel of human atherosclerotic aortic apoA-I recovered by immune-affinity isolation from 10 subjects and separated on a 12% reducing SDS-PAGE gel. In panel *B*, a parallel SDS-PAGE gel was run, and proteins transferred onto a membrane probed with high-affinity antihuman apoA-I-specific antibody (monoclonal antibody [mAb] 10G1.5) for Western blot analysis. Visual inspection of both Coomassie and Western blot gels (Fig. 2, *A* and *B*) reveals the presence of monomer at low levels and more abundant higher molecular weight forms of apoA-I. The presence of significant amounts of dimer and other apoA-I multimers in the SDS-PAGE denaturing gel (Fig. 2*A*) indicates that a substantial amount of apoA-I in the atherosclerotic artery wall is crosslinked, as previously reported (41).

Using stable isotope dilution LC–MS/MS, we quantified the content of protein-bound HCit levels in circulating blood from subjects (n = 117) with normal renal function and without evidence of CVD, diabetes, or other comorbidities (Table S1), and compared it with protein-bound HCit within the apoA-I recovered from human atherosclerotic aortic lesions (n = 10) (Table S2). Notably, apoA-I carbamylation within the human atherosclerotic artery wall (expressed as ratio to precursor amino acid, lysine) was significantly higher than that observed in total protein-bound HCit recovered from the plasma compartment (Fig. 2*C*).

### Establishing *in vitro* carbamylation conditions

To establish a clinically relevant *in vitro* level of apoA-I carbamylation, we performed dose–response experiments by varying the molar ratio of either CNO^−^ or the MPO/H_2_O_2_/SCN^−^ system to lipid poor apoA-I or rHDL (Fig. 2*D*). In general, the extent of total carbamylated lysine (HCit) produced was higher in lipid-poor apoA-I compared to rHDL with both model carbamylation systems, but few differences were otherwise noted in total HCit formation in comparison between CNO^−^ *versus* the MPO system. Importantly, from these studies, we concluded that the extent of apoA-I and rHDL lysine carbamylation induced by the *in vitro* systems at a molar ratio of 10:1 (carbamylating reagent: apoA-I) resulted in approximately the same degree of lysine carbamylation as observed in apoA-I recovered from human atheroma (Fig. 2, *C* and *D*). Accordingly, we used a 10:1 M ratio of carbamylating reagent to apoA-I for preparation of samples for proteomic characterization of the carbamylation sites.

### Distinct site-specific patterns of apoA-I lysine carbamylation are observed by CNO^−^*versus* the MPO/H_2_O_2_/SCN^−^ system

**Figure 3:**
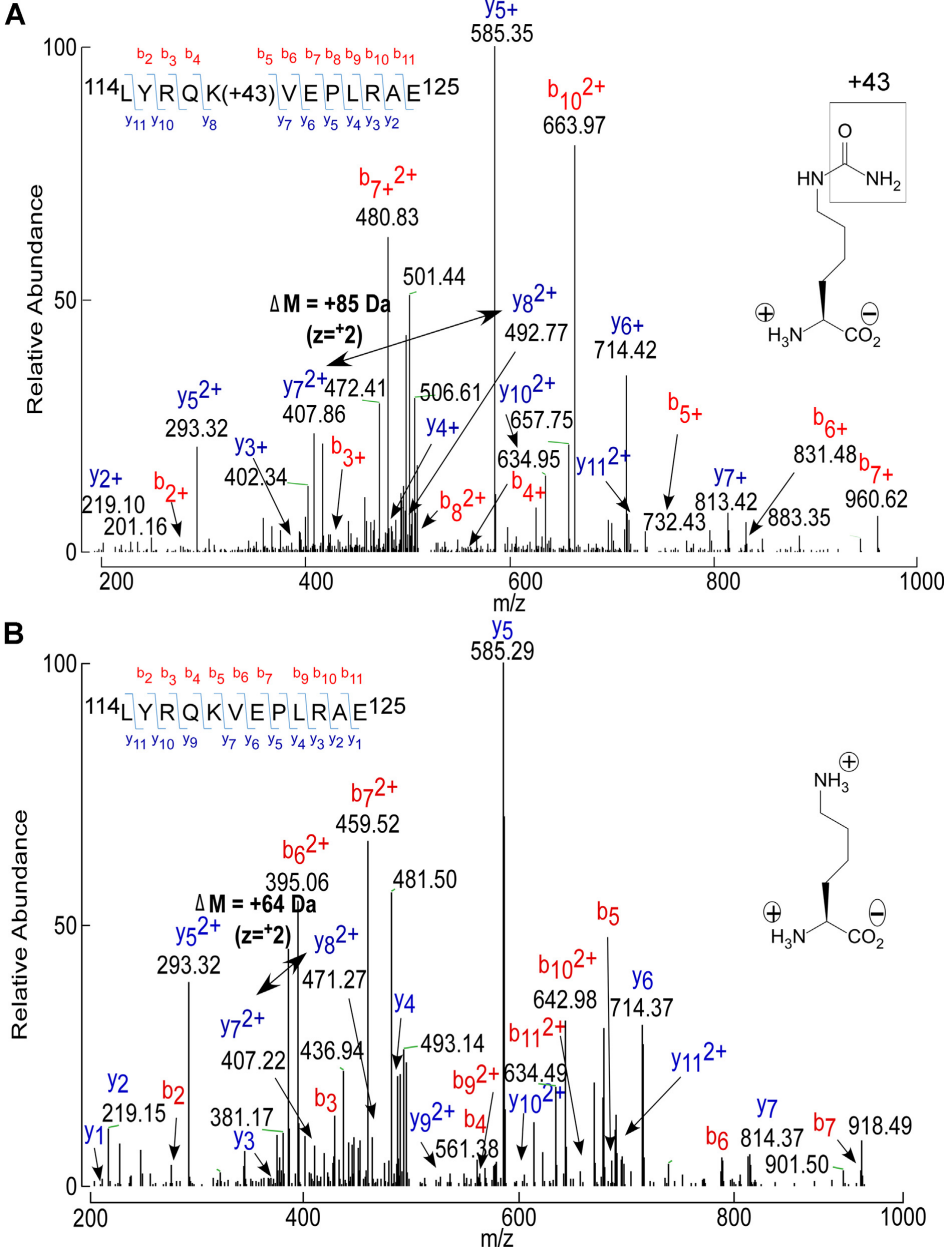
**LC–MS/MS spectra of GluC-digested peptide L**_**114**_–**E**_**125**_**containing either carbamylated K**_**118**_**or the unmodified K**_**118**_**of apoA-I.** Recombinant apoA-I samples were subjected to GluC protease digestion and LC–MS/MS proteomic analysis. *A*, MS/MS CID spectrum of L_114_YRQKVEPLRAE_125_ peptide containing the carbamylated lysine K_118_. This triply charged peptide has an *m/z* of 772.93 Da and was found within −0.56 ppm of its theoretical mass. *B*, MS/MS CID spectrum of L_114_YRQKVEPLRAE_125_ peptide containing unmodified K_118_. This unmodified peptide is triply charged with an *m/z* of 501.29 Da and was found within −1.91 ppm of its theoretical mass. The mass of the unmodified b_7_ ion is 918.40 Da and shifts to 960.62 Da when K_118_ is carbamylated. apoA-I, apolipoprotein A-I; CID, collision-induced dissociation.

Next, lipid-poor apoA-I and rHDL were carbamylated by either CNO^−^ or the complete MPO/H_2_O_2_/SCN^−^ system (10:1, carbamylating reagent: apoA-I), and then examined by quantitative proteomics. We were mindful that traditional tryptic digestion of proteins is sensitive to lysine modifications, so we employed GluC for protease digestion (see the Experimental procedures section). Peptide coverage for the *in vitro*-modified apoA-I was ≥90% for each of the four *in vitro* systems under investigation (apoA-I *versus* rHDL and CNO^−^ *versus* the MPO system), and is provided in Tables S5–S8. Of interest, of the 21 lysine residues within human apoA-I, only 14 were observed to be carbamylated, depending upon the *in vitro* carbamylation system employed. An example of the collision-induced dissociation (CID) spectrum of a typical apoA-I peptide recovered from rHDL harboring a carbamyllysine (HCit) residue *versus* its corresponding unmodified parent peptide (LYRQK_118_VEPLRAE) is shown in Figure 3. Notably, PTM generating the carbamyllysine residue (HCit) at K_118_ produces the anticipated mass addition of 43 Da at K_118_. In such a manner, the peptides harboring HCit observed in each of the *in vitro* systems (CNO^−^ *versus* MPO and rHDL *versus* lipid-poor apoA-I) were quantified and are listed in Tables S5–S8. Full scan CID mass spectra for each HCit harboring apoA-I peptide observed are provided in Figs. S6–S22.

**Figure 4:**
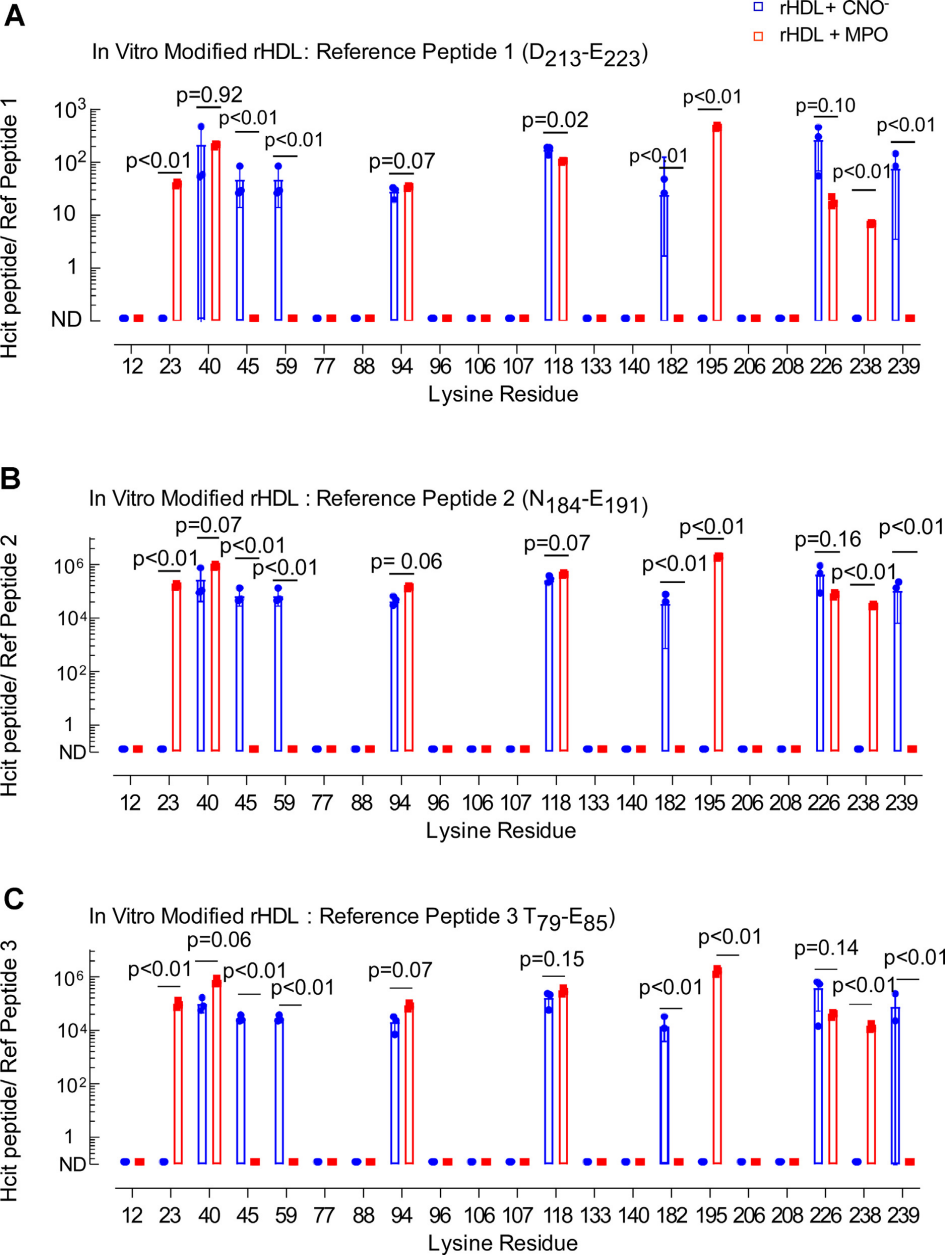
**Quantification of carbamylated rHDL-modified *in vitro* by the MPO or CNO**^**−**^**system.** Proteomics analysis of samples for peptide sequencing and quantitation of *in vitro*-modified rHDL. *A*–*C*, Ratios of peak area of modified lysine-containing peptides to peak area of individual reference peptides multiplied by 10^4^ are denoted as HCit Peptide/ref for each respective reference peptide. *p* Values were calculated using Student’s *t* test. CNO^−^, cyanate ion; HCit, homocitrulline; MPO, myeloperoxidase; rHDL, reconstituted high-density lipoprotein.

Aware that analyzing a purified recombinant protein used in *in vitro* experiments is less complicated than analyzing the same protein recovered from a complex environment (like human tissue), we sought to achieve a more robust quantification of the HCit content for site-specific residues both *in vitro* and *in vivo* within apoA-I samples. First, in early pilot studies, we noted that the parent (unmodified) peptide corresponding to highly carbamylated residues observed *in vitro* was sometimes not observed in apoA-I recovered from aortic tissues. We thus early on abandoned the idea of presenting results as a ratio of carbamylated relative to precursor (unmodified parent peptide). Therefore, we also quantified multiple different endogenous apoA-I native peptides that were simultaneously monitored in each sample to serve as reference peptides that could be used as a denominator to test for reproducibility of quantified data obtained (presented as a ratio of post-translationally modified peptide/reference peptide) (Fig. 4). As outlined in the Experimental procedures section, our control studies showed that the use of the adopted reference peptide strategy enabled semiquantitative assessment of apoA-I peptide levels, including within samples recovered from human atherosclerotic aortic tissues, with good reproducibility.

Focusing first on quantifying apoA-I site-specific carbamylation in rHDL, the relative abundance of apoA-I peptides harboring each detected HCit is shown in Figure 4. Along the *X*-axis, we indicate the lysine residue position in the primary amino acid sequence of mature apoA-I where protein-bound HCit was detected, whereas on the *Y*-axis, we plot the peptide abundance relative to the indicated reference peptide. Panels *A*, *B*, and *C* show results using each of three different reference peptides as denominator (selection of reference peptide was based on multiple criteria as outlined both in the Experimental procedures section and Table S4; full scan CID mass spectra for each selected apoA-I reference peptide used are provided in Figs. S23–S26). Of note, similar patterns of lysine carbamylation (both sites and relative abundances) were observed using each of the three different reference peptide candidates, instilling confidence in the relative label-free quantitation approach employed for peptides harboring HCit.

When selecting reference peptides, we looked for peptides that were readily detected by mass spectrometry (MS) in all samples and that did not contain lysine, methionine, histidine, or other nucleophilic or readily oxidizable residues that might be modified/oxidized either under the *in vitro* carbamylation systems examined or *in vivo*. However, when examining apoA-I isolated from the *in vitro* and *in vivo* samples, we noted not all reference peptide candidates showed comparable reproducibility in being observed (Table S4). The peptide D_213_–E_223_ was ultimately selected as the preferred reference peptide employed for all analyses because it was easily detected with high confidence in all *in vitro* and *in vivo* samples and represented the major peptide form observed for the indicated sequence (Table S4).

**Figure 5:**
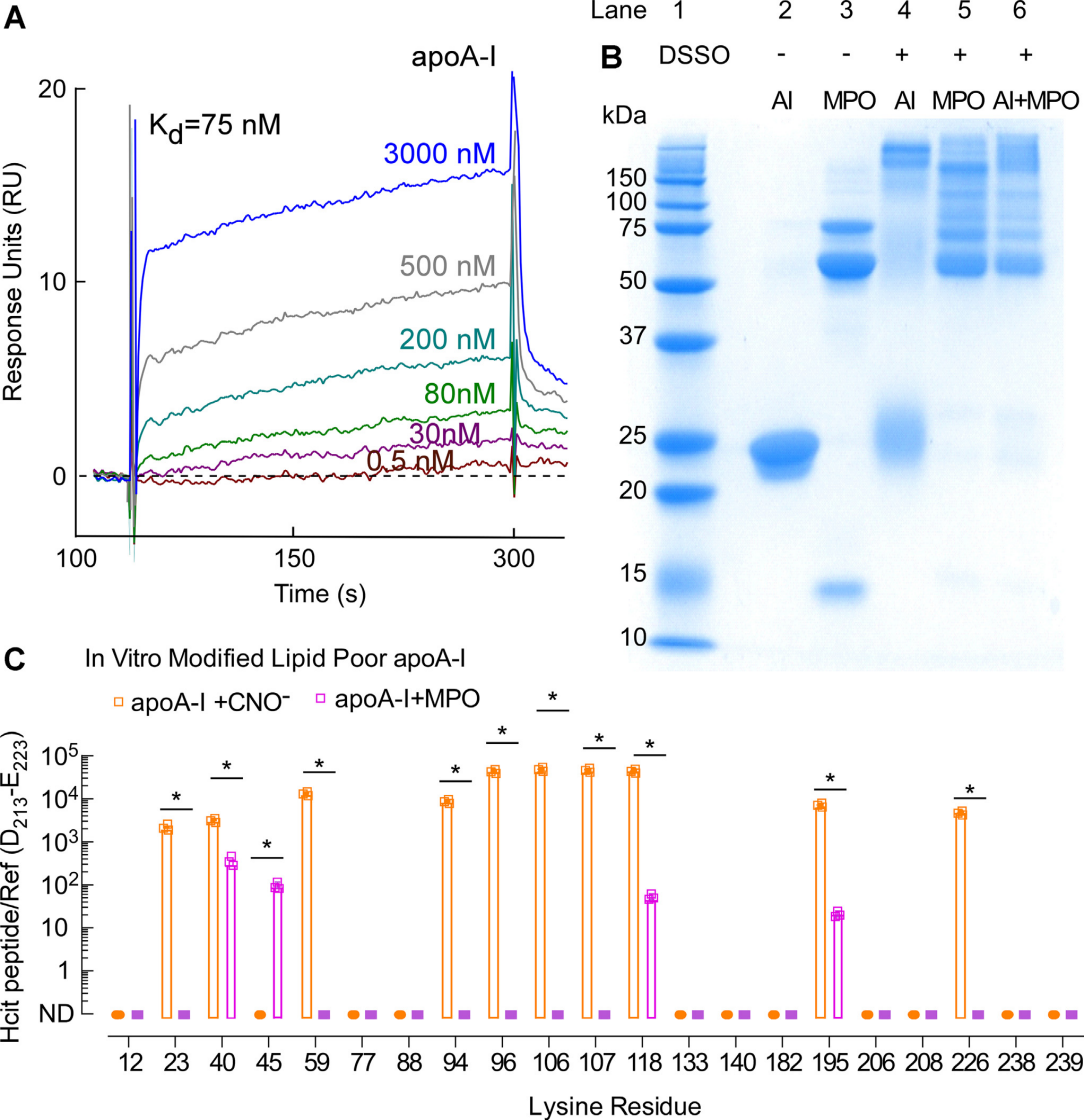
**SPR sensorgrams of lipid-poor apoA-I binding to MPO and quantification of lipid-poor apoA-I *in vitro* carbamylated by the MPO or CNO**^**−**^**system.***A*, SPR sensorgrams depicting MPO binding to lipid-poor apoA-I at various concentrations of apoA-I as indicated. MPO was immobilized on a CM5 sensor chip, and apoA-I was flown over the sensor chip surface in concentrations ranging from 0.5 nM to ∼3 μM as indicated. *B*, reducing SDS-PAGE gel of native and crosslinked apoA-I and MPO: lane 1: molecular weight marker; lane 2: apoA-I only; lane 3: MPO only, lane 4: apoA-I + DSSO (crosslinker); lane 5: MPO + DSSO; and lane 6: MPO + apoA-I + DSSO. *C*, ratios of peak area of *in-vitro*-modified lipid-poor apoA-I peptides (containing carbamylated lysine) to peak area of apoA-I reference peptide multiplied by 10^4^. *p* Values were calculated using Student’s *t* test. apoA-I, apolipoprotein A-I; CNO^−^, cyanate ion; DSSO, disuccinimidyl sulfoxide; MPO, myeloperoxidase; SPR, surface plasmon resonance.

While evaluating the pattern of site-specific apoA-I lysine carbamylation observed (using different carbamylation systems) may at first impression seem difficult, upon further examination some unique differences began to emerge. While high-affinity binding of MPO to apoA-I in rHDL has been reported (10, 42), the same is not the case for lipid-poor apoA-I. We hypothesized that the reproducible differences in apoA-I residue carbamylation observed between CNO^−^ *versus* the MPO/H_2_O_2_/SCN^−^ system might suggest MPO also binds with high affinity to lipid-poor apoA-I, enabling some residues to be preferentially carbamylated. Therefore, we sought to determine whether MPO binds to lipid-poor apoA-I. To measure the binding affinity of MPO interaction with lipid-poor apoA-I, we used surface plasmon resonance (SPR) spectroscopy as described under the Experimental procedures section. A strong binding interaction between lipid-poor apoA-I and MPO was observed (Fig. 5*A*). The apparent dissociation constant of MPO binding to apoA-I was calculated from the dose-dependent sensorgram data (Fig. 5*A* and Table S11), indicating nanomolar range affinity (*K*_*D*_ = 75 nM). In addition, we used the disuccinimidyl sulfoxide (DSSO) crosslinker to determine whether MPO and apoA-I can be crosslinked, indicative of a higher molecular weight complex. Lane “AI + MPO” in the Coomassie gel in Figure 5*B* shows that MPO and lipid-poor apoA-I readily form high–molecular weight complexes upon crosslinking with DSSO. The presence of both apoA-I and MPO within high–molecular weight complexes was further confirmed by Western blot analysis, revealing both immunoreactive apoA-I and MPO within multiple high–molecular weight bands (Fig. S2).

Next, the same approach for the quantification of HCit content (as previously done with rHDL) was used for lipid-poor apoA-I modified *in vitro* by either CNO^−^ or the MPO system. Figure 5*C* depicts the site-specific pattern of lipid-poor apoA-I carbamylation and HCit abundances (reported as the ratio of peptide harboring HCit relative to the peak area of the reference peptide D_213_–E_223_). Like in the case of rHDL, it is evident that the enzymatic (MPO) and nonenzymatic (CNO^−^) carbamylation systems produce distinct patterns of apoA-I lysine residue PTM, with MPO showing a more limited and localized carbamylation pattern (*open purple bars*, Fig. 5*C*) than CNO^−^ (*open orange bars*, Fig. 5*C*). Upon more detailed examination, it is noticeable that the MPO system exclusively carbamylates K45 (*open purple bar*, Fig. 5*C*), whereas CNO^−^ selectively carbamylates K23, K59, K94, K96, K106, K107, and K226 (*open orange bars*). Furthermore, K40, K118, and K195 were carbamylated by both MPO and CNO^−^ systems. More details about the HCit harboring apoA-I peptide quantitation of lipid-poor apoA-I using the *in vitro* carbamylation systems are shown in Fig. S3 and Tables S7 and S8. Each of the full-scan CID fragmentation spectra of apoA-I peptides harboring PTMs used in our proteomics analyses is given in the Supporting Information section.

### The pattern of apoA-I lysine carbamylation within human atherosclerotic aorta indicates the protein is being exposed to a complex reactive environment

**Figure 6:**
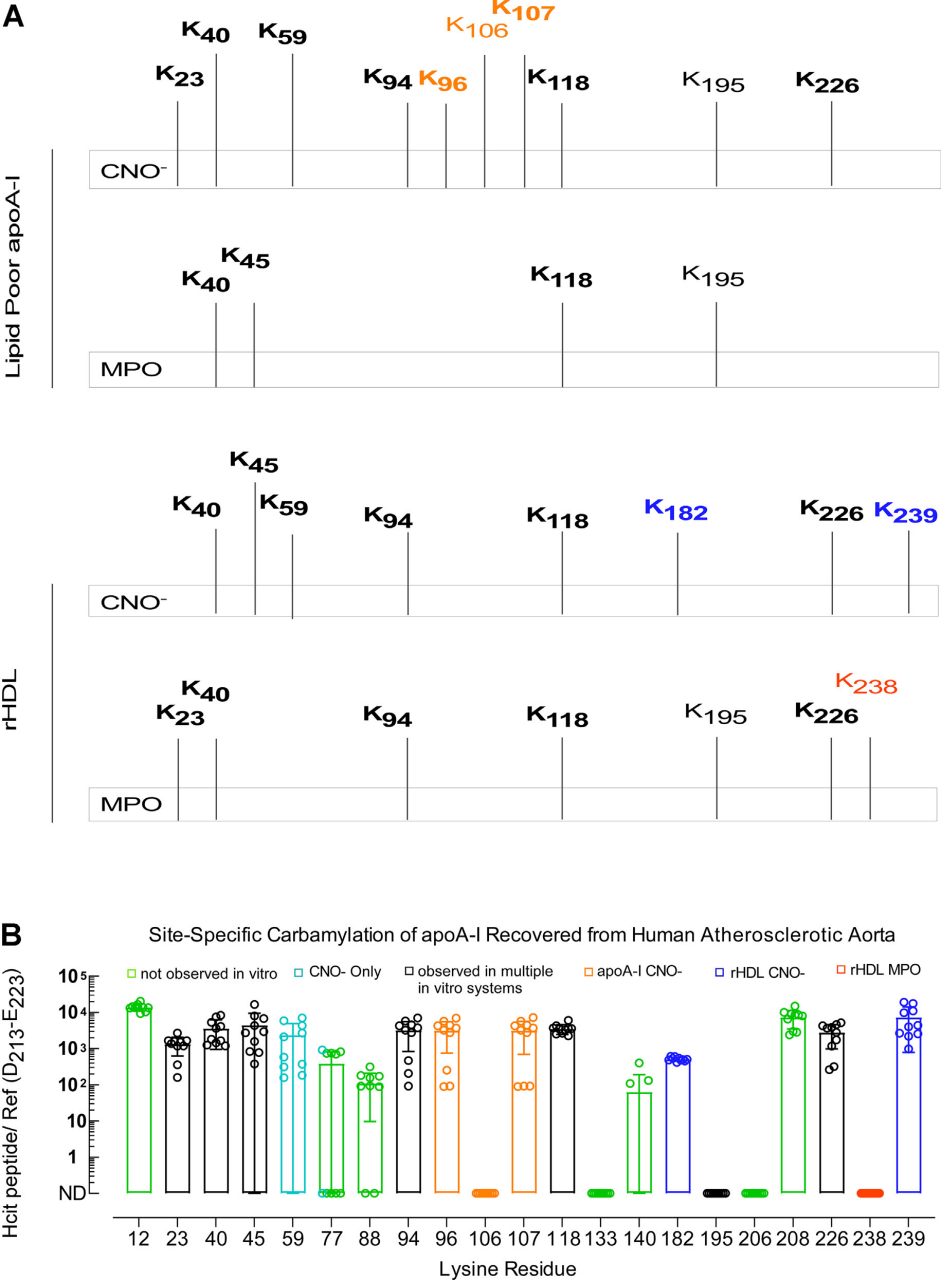
**Summary of *in vitro* carbamylated lysine residues in lipid-poor apoA-I/rHDL and quantification of carbamylated apoA-I recovered from human atherosclerotic aorta.***A*, summary of lysine residues carbamylated by the CNO^−^ or MPO system in lipid-poor apoA-I (*top*) and in rHDL (*bottom*). *B*, proteomics analysis of samples for peptide sequencing and quantitation from apoA-I isolated from human lesions. Quantitation is shown as ratios of peak area of modified lysine-containing peptides to peak area of the reference peptide multiplied by 10^4^. apoA-I, apolipoprotein A-I; CNO^−^, cyanate ion; MPO, myeloperoxidase; rHDL, reconstituted high-density lipoprotein.

ApoA-I recovered from human atherosclerotic lesions (n = 10 subjects) was subjected to quantitative proteomic analyses similar to that performed for the *in vitro*-modified rHDL and lipid-poor apoA-I. Table S2 lists peptide coverage observed (>90%) following digestion and proteomics analysis of apoA-I isolated from distinct (n = 10) human atherosclerotic aortas. The results of site-specific carbamylation observed in the recovered apoA-I are shown in Figure 6*B*. Also displayed using the same *X*-axis (lysine location in apoA-I) is the summary of the patterns of site-specific apoA-I carbamylation observed *in vitro* using either the CNO^−^ or MPO systems, for lipid-poor apoA-I and rHDL (Fig. 6*A*). For the *in vivo* apoA-I data, the values displayed on the *Y*-axis are again the ratios of the peak area of peptides containing modified lysines (*i.e.*, HCit) to the peak area of the reference peptide D_213_–E_223_. Quantitation of HCit in the *in vivo* apoA-I samples using each of the three individual reference peptides showed comparable results (Fig. S4). Furthermore, fragmentation spectra of each of the human aortic tissue–derived apoA-I peptides harboring a carbamylated lysine used in these analyses are given in the Supporting Information section.

Review of the apoA-I site-specific carbamylation data from human aortic tissues shows several interesting findings. First, a larger number of apoA-I lysine residues (16 of 21) were observed to be carbamylated than were carbamylated within apoA-I modified by either CNO^−^ or the MPO/H_2_O_2_/SCN^−^ system *in vitro*, and with either lipid-poor or rHDL forms. Thus, several residues (*e.g.*, K12, K77, K88, K140, and K208), carbamylated in apoA-I recovered from the aortic lesions, were not detected using any of the *in vitro* carbamylation systems (Fig. 6*B*, *green bars*). Second, overall, the sites of carbamylation observed in apoA-I recovered from human aorta represented a mixture of those observed as being carbamylated *in vitro* by either CNO^−^ or MPO systems, on both lipid-poor apoA-I and rHDL.

For ease of comparing the *in vivo versus in vitro* site-specific carbamylation data for apoA-I, the carbamylated lysine residues detected *in vitro* (Fig. 6*A*) are vertically aligned with the carbamylated lysine residues detected *in vivo* (Fig. 6*B*), and the lysine residues carbamylated also *in vivo* are labeled in *boldface* in Figure 6*A*. Despite observing distinct patterns of lysine residues carbamylated *in vitro* by MPO *versus* CNO^−^ for both rHDL and lipid-poor apoA-I, we noticed that K238 (labeled *red*), the only apoA-I lysine residue specifically carbamylated *in vitro* by MPO only in rHDL, was not observed in carbamylated form in apoA-I recovered from aortic tissues (Fig. 6*B*). On the other hand, K182 and K239 (labeled *blue*), the only lysine residues specifically modified *in vitro* by CNO^−^ in rHDL, were detected *in vivo*. This suggests that, at least in part, the carbamylated apoA-I recovered from human atherosclerotic plaque originates from HDL carbamylated by urea-derived CNO^−^. In addition, K96 and K107 (labeled *orange*), specifically modified *in vitro* by CNO^−^ in lipid-poor apoA-I, were also observed *in vivo*, suggesting that carbamylated apoA-I recovered from human atherosclerotic tissue originates in part from lipid-poor apoA-I carbamylated by CNO^−^.

Other apoA-I lysine residues carbamylated *in vitro* either by MPO or CNO^−^ observed *in vivo* are K23, K40, K45, K59, K94, K96, K107, K118, K182, and K226. Their detection implies that the *in vivo* carbamylated apoA-I may originate either from an HDL particle or lipid-poor form of apoA-I. On the other hand, the detection of carbamylated K12, K77, K88, K140, and K208 only in apoA-I recovered from human atherosclerotic plaque suggests that in the artery wall, apoA-I may also be present in molecular states different from that examined in the lipid-poor and rHDL forms examined here, such as might exist for highly crosslinked and heavily oxidized apoA-I (41). In addition, we recognize that HDL is heterogeneous *in vivo* (*i.e.*, there are many subclasses of particles with more than two apoA-I chains per particle and various lipid compositions (43)), which may also contribute to the difference in lysine carbamylation patterns observed *in vivo versus in vitro*. We note, however, that previous studies by others using an alternative MPO-generated oxidant, HOCl, concluded that distinct HDL subclasses did not influence their intrinsic susceptibility to oxidative attack by HOCl (44). Finally, we note that there were three lysine residues (K106, K195, and K238) carbamylated *in vitro* but not observed *in vivo*: (i) K106, which was specifically modified by CNO^−^ when in lipid-poor apoA-I; (ii) K195, which was modified by either MPO or CNO^−^ when in rHDL and by CNO^−^ when in lipid-poor apoA-I; and (iii) K238, which was carbamylated by MPO exclusively but only in rHDL.

**Figure 7:**
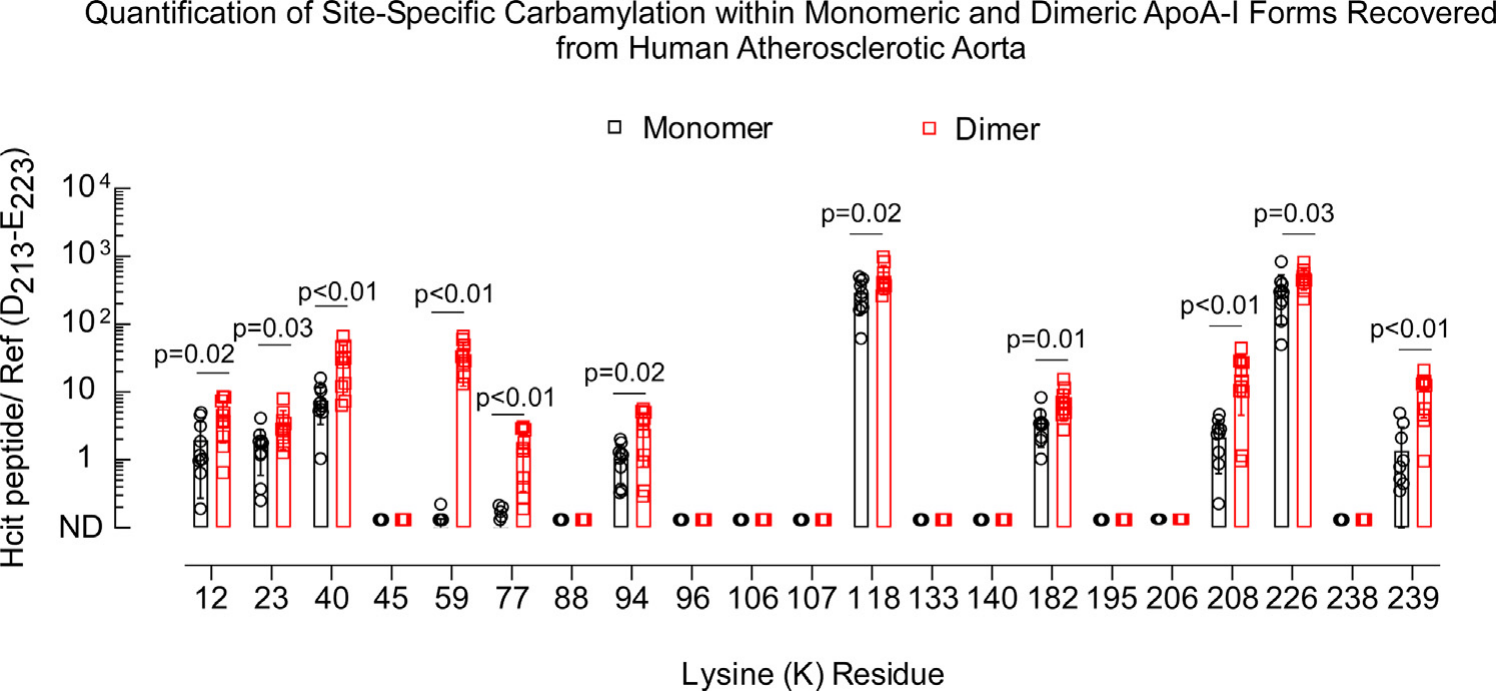
**Quantifications of monomeric and dimeric carbamylated apoA-I recovered from human atherosclerotic aorta.***A*, in-gel digestion proteomics analysis of samples for peptide sequencing and quantitation from monomeric and dimeric apoA-I isolated from human lesions. Quantitation is shown as ratios of peak area of modified lysine-containing peptides to peak area of the reference peptide as described in the Experimental procedures section. apoA-I, apolipoprotein A-I.

We noted earlier that apoA-I in the aortic wall is substantially crosslinked, predominantly existing in the dimeric form (Fig. 2, *A* and *B*). Therefore, we further inquired whether the extent or pattern of lysine carbamylation is different in monomeric *versus* dimeric apoA-I, as described under the Experimental procedures section. Our analysis showed that dimeric apoA-I was more extensively carbamylated than monomeric apoA-I recovered from human aortic lesions, suggesting carbamylation occurred to a greater extent in the more heavily oxidized and cross-linked dimeric apoA-I form (Fig. 7). Moreover, the site-specific pattern of carbamylation was similar in direct comparisons of monomeric *versus* dimeric apoA-I forms except for lysine residues K59 and K77, which were only substantially carbamylated in the apoA-I dimeric form.

### Pattern of apoA-I lysine residue carbamylation as a tool to interrogate current models of rHDL and lipid-poor apoA-I

**Figure 8:**
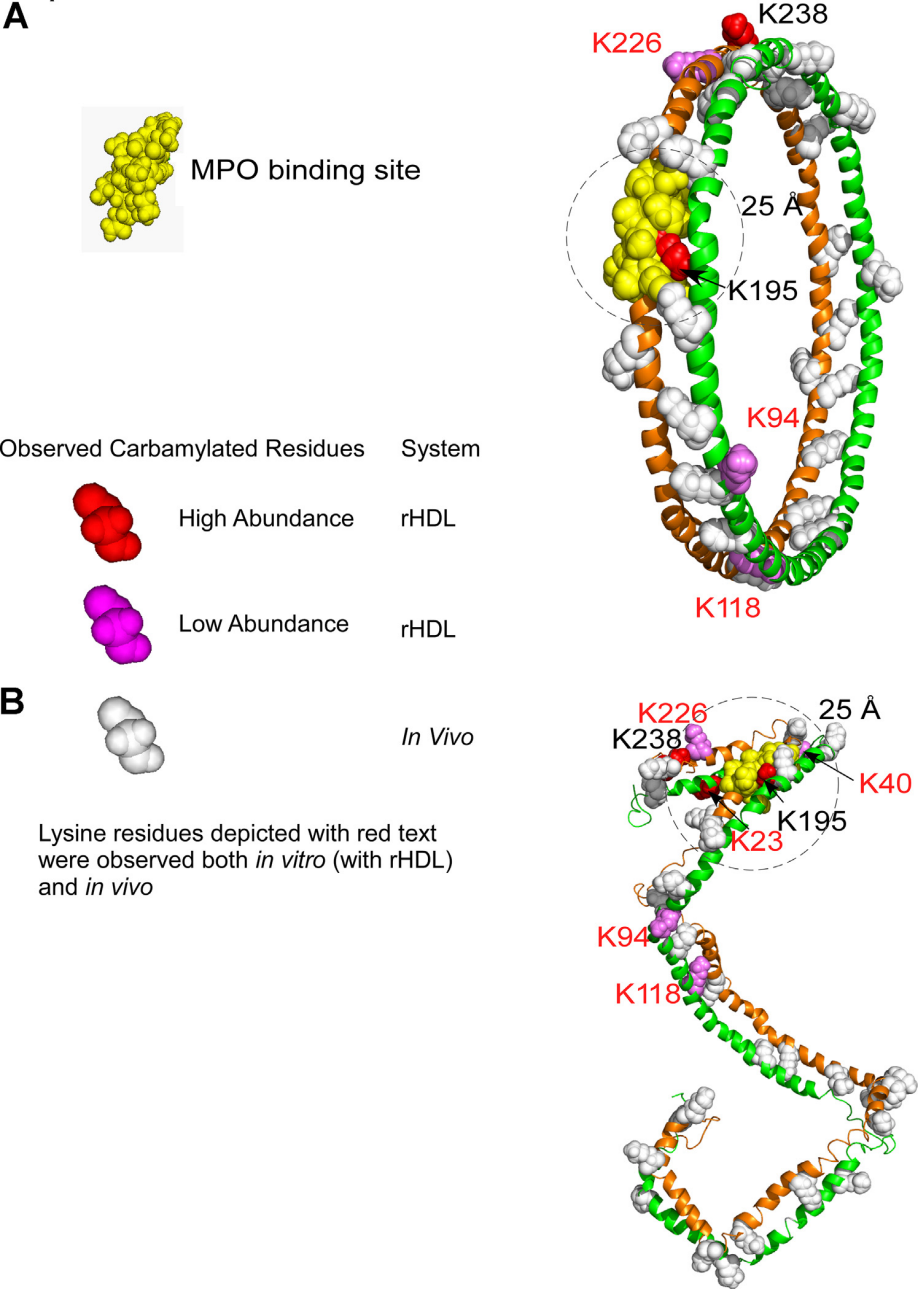
**Mapping *in vivo* detected or MPO *in vitro* carbamylated lysine residues onto models of rHDL.***A*, carbamylated lysine residues depicted in the Double Belt model of rHDL. The carbamylated lysine residues are shown explicitly as *spheres*, whereas the rest of the protein is shown in *cartoon* (secondary structure) representation. The carbamylated lysine residues are colored *red* (*red spheres*) if found in high abundance and in *violet* if found in lower abundance. *White spheres* with *red text* indicate carbamylated lysine residues observed either *in vivo* or *in vitro*. The MPO-binding site (A_190_–L_203_) is depicted with *yellow spheres*. *B*, carbamylated lysine residues depicted in the Double Superhelix (DSH) model of rHDL. The same color-coding scheme was used as for the Double Belt model. The DSH model has two distinct and unsymmetrical MPO-binding sites, and five carbamylated lysine residues out of seven are less than 25 Å (*circled area*) from this MPO-binding site (*top*). MPO, myeloperoxidase; rHDL, reconstituted high-density lipoprotein.

The idea that protein structure can be investigated by decorating the protein surface with PTMs during *in vitro* modification has previously been used as a tool to further interrogate protein structure (45, 46, 47, 48). We therefore posed the question whether apoA-I lysine carbamylation in rHDL produced *in vitro* can be used to further interrogate models for the structure of rHDL and lipid-poor apoA-I. Popular models of rHDL have been reported using various biochemical and computational methods (11, 49, 50). In a final series of analyses, we hypothesized that the pattern of lysine carbamylation observed in *in vitro* model systems can be used to further interrogate currently accepted models for the structure of rHDL, for example, the Double Belt model (49) *versus* the Double SuperHelix (DSH) model (51), and a lipid-poor apoA-I model based on small angle X-ray measurements (52). In the Double Belt model of rHDL, two N-terminus-truncated (D_1_–N_43_) apoA-I chains are oriented antiparallel and stacked on top of each other in a closed circular belt, wrapping around a lipid bilayer (49). Earlier studies interrogated the location of MPO interaction on apoA-I in rHDL using hydrogen-deuterium exchange MS, revealing a presumed MPO-binding site (apoA-I domain A_190_–L_203_) where upon MPO binding, hydrogen-deuterium exchange of backbone amide hydrogens is hindered (10). Because rHDL contains two antiparallel apoA-I chains, there are two potential sites for MPO binding; however, the carbamylation patterns produced by MPO bound to either site on an rHDL particle described by the Double Belt model should be indistinguishable because this model is symmetric. In Figure 8*A*, we show the structure of the Double Belt model with lysine residues in rHDL observed to be carbamylated *in vitro* by the MPO system indicated (*red and violet spheres*). Also mapped are lysine residues carbamylated within apoA-I recovered from human atherosclerotic aorta; lysine residues displayed with *white spheres* or labeled with *red text* were observed *in vivo*. K195 and K238 (labeled with *black text*) were not observed *in vivo*. Also shown is a theoretical “zone” of significant probability of apoA-I carbamylation by MPO with a sphere of 25 Å radius (*black dotted line*—for visual inspection/illustration only) centered on the previously mapped MPO-binding site (A_190_–L_203_, *yellow spheres*). Of the seven apoA-I lysine residues in rHDL carbamylated *in vitro* by MPO (K23, K40, K94, K118, K195, K226, and K238), only lysine K195 is within this “effective” carbamylation space in the Double Belt model (Fig. 8*A*). K23 is not present because the Double Belt model uses an N-terminal truncated mutant of apoA-I. While K238 in rHDL was observed to be carbamylated by MPO in high abundance, it is well outside the 25 Å radius zone, as are the other less abundant carbamylated K40, K94, K118, and K226.

In contrast to the Double Belt model, the DSH model of rHDL is based on small-angle neutron scattering with contrast variation analysis of rHDL particles reconstituted with full-length apoA-I (51). The two apoA-I chains in this latter model are also arranged antiparallel but in an open conformation wrapping around a partially micellar lipid phase (51). One of its distinctive features is that the two N termini/C termini are open and not symmetric (in contrast to the Double Belt model), which makes the two MPO-binding sites distinct (Fig. 8*B*). Notably, the arbitrary 25 Å radius space around the MPO-binding site in this model of rHDL includes the majority (five of seven) of the carbamylated lysine residues (K23, K40, K195, K226, and K238) produced by the MPO system. We conclude that the open unsymmetrical helical architecture of apoA-I in the DSH model seems better suited to account for the pattern of carbamylated lysine residues in rHDL produced *in vitro* by the MPO system.

In separate analyses, the eight lysine residues in rHDL modified by urea-derived CNO^−^ (K40, K45, K59, K94, K118, K182, K226, and K239) were mapped on the Double Belt and DSH models and are shown in Fig. S5. The highly abundant carbamylated lysine residues are colored with *dark blue*, and we notice that in both HDL models (Fig. S5, *A* and *B*), they are nearly uniformly spread throughout the protein chain. The *white spheres* indicate carbamylated lysine residues observed *in vivo* and *in vitro*. Lysine residues labeled with *black text* were observed to be carbamylated only *in vitro*.

**Figure 9:**
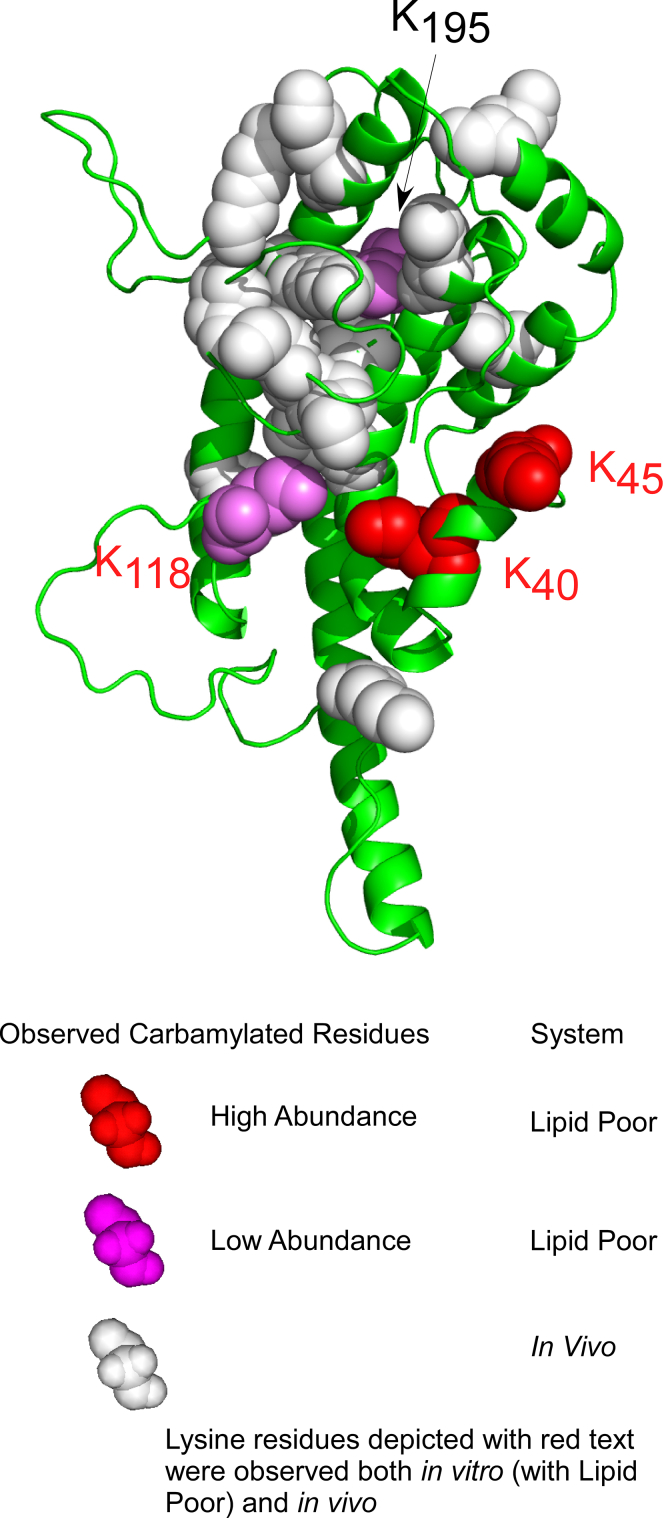
**Lysine residues carbamylated in lipid-poor apoA-I mapped onto a model of lipid-poor apoA-I.** Lysine residues carbamylated *in vitro* by the MPO/H_2_O_2_/SCN^−^ system are depicted with *red spheres* if they were found in high abundance and with *violet* if found in low abundance. Carbamylated lysine residues shown with *white spheres* were detected *in vivo*. Carbamylated lysine residues with *red labels* were detected either *in vitro* or *in vivo*. apoA-I, apolipoprotein A-I; MPO, myeloperoxidase; SCN^−^, thiocyanate ion.

We next focused our attention on whether the observed pattern of apoA-I lysine residue carbamylation in lipid-poor apoA-I could serve as a tool to interrogate the structure of lipid-poor apoA-I. While several models of full-length lipid-poor apoA-I have been proposed (52, 53, 54), in this study, we used a model based on small-angle X-ray measurements published earlier (52). In Figure 9, we applied the same color scheme as in Figure 8 to show lysine residues in lipid-poor apoA-I that were carbamylated *in vitro* by the MPO/H_2_O_2_/SCN^−^ system (K40, K45, K118, and K195). Since the MPO-binding site on lipid-poor apoA-I has not yet been identified, we do not know which lysine residues are in close proximity to MPO when bound to lipid-poor apoA-I, but we note that the lysine residues carbamylated either *in vitro* or *in vivo* (*white spheres*) are clustered together, unlike in the rHDL models. This is perhaps not surprising considering that in this particular model of lipid-poor apoA-I, the protein is rather compact and much smaller than rHDL. The 10 lysine residues in lipid-poor apoA-I observed to be carbamylated *in vitro* by CNO^−^ (K23, K40, K59, K94, K96, K106, K107, K118, K195, and K226) were separately mapped on the model of lipid-poor apoA-I and shown in Fig. S5*C*. The five (of 10) carbamylated lysine residues of lipid-poor apoA-I (K59, K96, K106, K107, and K118) detected in highest abundance are colored *dark blue*. As in the case of rHDL, the carbamylated lysine residues are spread along the entire protein chain, but because of the compactness of the apoA-I monomer in this model, many of the carbamylated lysine residues are clustered together.

## Discussion

Our overarching hypothesis in the present studies was that because MPO binds tightly (nanomolar affinity) to apoA-I in both rHDL (10) and lipid-poor forms (this study), the residues within apoA-I that are carbamylated by MPO-generated CNO^−^ should be in close proximity to the MPO-binding site. Thus, the patterns of site-specific modification of apoA-I recovered from human atherosclerotic lesions may provide insights into the environment in which apoA-I is carbamylated within a plaque-laden artery wall. Indeed, our initial *in vitro* studies demonstrate that the original concept has merit since both rHDL and lipid-poor apoA-I show distinct patterns of site-specific lysine carbamylation when subjected to the MPO/H_2_O_2_/SCN^−^ system *versus* free diffusion (*i.e.*, the urea-driven CNO^−^ system). Thus, we observed that when using the MPO/H_2_O_2_/SCN^−^ system, nucleophilic residues in apoA-I near the MPO-binding site were preferentially modified. Therefore, the *in vitro* data observed suggest the ability to potentially interrogate the following questions—(i) whether apoA-I is carbamylated by MPO/H_2_O_2_/SCN^−^ *versus* CNO^−^ and (ii) whether in HDL or lipid-poor form.

Our proteomics analysis of apoA-I recovered from human lesions shows extensive carbamylation. Based on the differential sites of *in vitro* carbamylation observed in lipid-poor apoA-I and rHDL, we would conclude that lysine modification in apoA-I recovered from human aorta tissue is consistent with it being carbamylated predominantly in a lipid-poor form. Of note, some of the carbamylated lysine residues found in apoA-I isolated from human lesions were not detected as being sites of carbamylation in our *in vitro* studies. However, a significant number of lysines preferentially carbamylated *in vivo* overlap with those modified in lipid-poor apoA-I or HDL *in vitro* by MPO and CNO^−^. Only one apoA-I lysine residue was observed in rHDL (K238) to selectively be modified by MPO exclusively, and not by the *in vitro* CNO^−^ system, and this residue was not detected as a site carbamylated in apoA-I recovered from human lesions. However, multiple lysines in lipid-poor apoA-I carbamylated *in vitro* by the MPO system were observed in the apoA-I recovered from human aortic lesions. We were also intrigued by the fact that K195, which was extensively carbamylated *in vitro* by the MPO system in rHDL, was not observed as a site carbamylated *in vivo* despite being in close proximity to the MPO-binding site on HDL/apoA-I. This suggests a conformational change of apoA-I around K195 in the artery wall that hinders its susceptibility for carbamylation, such as involvement in a salt bridge or limited solvent accessibility. We also note that the absence of seeing any specific carbamylated peptide merely indicates it was below our limit of detection and does not rule out its existence *in vivo*.

One interesting finding was that dimeric forms of apoA-I harbored a greater extent of carbamylation than monomeric forms. One logical explanation of this finding is that some of the same sources of oxidative crosslinking (*i.e.*, MPO) in apoA-I in lesions also can foster carbamylation. However, we also note that oxidatively cross-linked dimeric apoA-I forms may have longer residence times in tissues and thus may accumulate PTMs like carbamyllysine to a greater extent. So in itself, observing a higher level of carbamylation in dimeric apoA-I forms cannot be taken as evidence of MPO as a source of that heightened degree of carbamylation. Interestingly, apoA-I K59 and K77 were significantly more carbamylated in the apoA-I dimer, than in the monomer, in contrast to the other apoA-I sites where the difference in carbamylation between dimer and monomer is less pronounced. These results also suggest that within the apoA-I dimer in the artery wall, K59 and K77 may also be more solvent exposed to the carbamylating environment (relative to the monomer).

Multiple prior studies demonstrated that HDL is a selective target for MPO-catalyzed oxidative modifications *in vivo* (10, 14, 15, 16). Notably, carbamylation levels of apoA-I recovered from lesions reported here were higher than 3-chlorotyrosine levels, a specific PTM of reactive halogenating species generated exclusively by MPO (10, 26, 40, 55). In addition, MPO-derived chlorinating species have been shown to rapidly decompose SCN^−^ and urea, thus producing another carbamylation source within the artery wall (56).

Examining the patterns of apoA-I carbamylation by MPO provides insights into the structure of defined rHDL particles that are generated by the traditional cholate dialysis methods (at a molar ratio of 100:10:1, 1-palmitoyl-2-oleoyl-*sn*-glycero-3-phosphocholine:cholesterol:apoA-I). Our analysis of the *in vitro* carbamylation data for rHDL indicates that the DSH model of rHDL accounts better for the pattern of carbamylated lysine residues produced by MPO. Specifically, our analysis shows that one of the previously identified MPO-binding sites on apoA-I in the DSH model clusters five (of seven) carbamylated lysine residues near the center of the MPO-binding site. By comparison, the Double Belt model accommodates only one carbamylated lysine within the same space.

This study has several limitations. First, it is notable that the analysis of carbamylation in human aortic tissue is limited to diseased lesion tissue, given the difficulty in accessing normal human aorta tissue samples. In addition, many of the carbamylated lysine residues in apoA-I recovered from human lesions were not detected in our *in vitro* studies. The reasons for this are likely manifold. While urea-driven and MPO-catalyzed protein carbamylation are the major pathways known, there may be additional unrecognized mechanisms responsible for carbamylation. More likely, our studies were limited to testing two *in vitro* carbamylation pathways against two presumed abundant conformations of apoA-I, namely, lipid poor and rHDL (albeit of a single lipid composition). Alternative structures of apoA-I no doubt exist within the artery wall, including the multiplicity of HDL forms that exist as variations in lipid cargo occur. In addition, previous studies demonstrated that the majority of apoA-I recovered from human lesions is not HDL associated but rather exists predominantly in lipid-poor form (41). Moreover, apoA-I in human arterial tissues is heavily oxidized and crosslinked and thus may exists in various alternative conformations (41).

Indeed, in the present studies, apoA-I recovered from aortic lesions showed a high degree of crosslinking and multimeric molecular weight forms (represented the majority of apoA-I protein by both Coomassie staining recovered and Western analyses with anti-apoA-I antibodies). As a result, this would alter the detection of peptides by the MS. We did observe differences in the GluC enzyme digestion pattern, for example, larger (miscleaved) unmodified peptides with multiple possible modification sites were found. Finally, another potential limitation of the present studies is that the quantification of site-specific PTMs in an extensively oxidized and cross-linked protein from complex tissue samples of multiple compositions is difficult to normalize across studies. Typically, one might present the post-translationally modified peptide as a ratio with its unmodified parent form (in the denominator). But as noted, in early pilot studies, we found that many of the parent peptides were simply not detectable in apoA-I recovered from arterial tissues. Moreover, protease cleavage of proteins is a critical step in MS analyses, and oxidative and PTMs of apoA-I significantly impacted the reproducibility of protease cleavage and peptide recovery following oxidation. We attempted to address these issues by using an atypical protease cleavage enzyme not sensitive to lysine modification (as opposed to trypsin). We also tried alternative quantitative proteomics methods including isobaric tagging and use of recombinant ^15^N-labeled apoA-I as internal standards. However, both of these approaches were difficult to implement (see the Experimental procedures section). After extensive screening, we ultimately settled on utilizing an endogenous reference peptide approach (with three distinct referent peptides, each of which were identified with high confidence and in high abundance in all examined *in vitro* and *in vivo* samples). Moreover, results using each of the three distinct referent peptides provided comparable qualitative results, offering further confidence in the overall approach.

One major advancement with the present study is the mapping of lysine carbamylation sites observed in apoA-I recovered from diseased arterial tissues. Our more current understanding of the relationship between HDL and CVD emphasizes the importance of the function of apoA-I/HDL, instead of the quantity of its cholesterol cargo (reviewed in Refs. (40, 57, 58, 59)). Furthermore, past studies have shown that carbamylation of HDL impacts its function, including loss of antiapoptotic activity (19) and changes in intracellular cholesterol metabolism in macrophages with only one carbamylated lysine residue per HDL being sufficient to induce cholesterol accumulation and lipid droplet formation (40). In more recent studies, carbamylation of HDL has also been reported to induce loss of anti-inflammatory and antioxidative activities (56), reduction in the extent of cholesterol efflux from cholesterol-loaded macrophages (35), and significant inhibition in multiple endothelial cell repair functions observed with native apoA-I or HDL but not with carbamylated forms (60). Carbamylation of HDL has also been reported to decrease the ability of HDL to activate lecithin-cholesterol acyltransferase activity, an essential step in particle maturation and reverse cholesterol transport (56). In addition, carbamylated HDL has been shown to result in a gain of proinflammatory function through the promotion of monocyte adhesion (61). Finally, carbamylation of HDL has been reported to decrease paraoxonase activity (a major HDL-associated anti-inflammatory enzyme), potentially serving as a mechanism for adversely impacting the antioxidative and anti-inflammatory functions of HDL (56). For all these aforementioned studies, the sites of carbamylation associated with these functional changes were not explored. The present studies are the first to map the sites carbamylated on apoA-I *in vitro* and in apoA-I recovered from atherosclerotic diseased artery wall. They thus are a critical step in further efforts to examine the role of site-specific apoA-I carbamylation on impacting the myriad of functions attributed to apoA-I and HDL to be examined in future studies.

## Experimental procedures

### General procedures

Human MPO was purified as previously described (10). Briefly, MPO from human leukocytes was purified by sequential lectin affinity and gel filtration chromatography (62), and any trace contaminating eosinophil peroxidase was removed through sulphopropyl Sephadex column chromatography (63). MPO purity was established by showing Reinheit Zahl value greater than 0.84 (absorbance at 430 nm/absorbance at 280 nm), SDS-PAGE analysis with Coomassie blue staining, and in-gel tetramethylbenzidine peroxidase staining (64). The concentration of MPO was determined spectrophotometrically using an absorption coefficient of 89,000 M^–1^ cm^–1^ per heme (65). Crystalline catalase from bovine liver was purchased from Sigma (catalog number: C1345). H_2_O_2_ concentrations were determined spectrophotometrically (molar absorption coefficient, ε_240_ = 39.4 M^–1^ cm^−1^) before use (66). SDS-PAGE was performed using 12% polyacrylamide gels to fractionate reduced proteins as described by Laemmli (67). For Western blot analyses, proteins were transferred onto polyvinylidene difluoride (Immobilon-FL) membrane and then developed for detection and quantification using the Odyssey imaging system (LI-COR Biosciences). Membranes were probed with an anti-apoA-I mAb 10G1.5 developed and characterized previously (41) and then quantified for apoA-I abundance following use of Li-Cor IRDye 680RD secondary antibodies (catalog number: 925-68070; LI-COR Biosciences), as previously described (41).

### Quantification of total protein-bound HCit

Total protein-bound HCit was quantified in human lesions and *in vitro* apoA-I modified studies with either lipid-poor apoA-I or rHDL samples, using a previously described stable-isotope dilution HPLC with online tandem MS method (20). Analyses were performed following delipidation, desalting, and overnight protein hydrolysis with hydrochloric acid. [^13^C_6_, ^15^N_2_]Lys and [^13^C, ^15^N]HCit were added to the protein pellet, and samples were then hydrolyzed overnight with hydrochloric acid. [^13^C, ^15^N]HCit was synthesized by reaction of polylysine with SCN^−^ and H_2_O_2_ in the presence of MPO followed by acidic hydrolysis and purification, as described previously (68). Sample acid hydrolysates were passed over mini solid-phase DSC-SCX extraction columns (Discovery DSC-SCX SPE tubes; 1 ml; Supelco, Inc), and analytes (lysine and HCit) were resolved following injection onto a Scherzo SW-C18 column (3 × 150 mm) (Imtakt USA) interfaced to a Shimadzu 8050 triple-quadrupole mass spectrometer. The Shimadzu UPLC was multiplexed to the tandem mass spectrometer with analytes ionized using electrospray ionization in positive-ion mode with multiple reaction monitoring of parent and characteristic daughter ions specific for each analyte, as previously described (20). The UHPLC system used a discontinuous mobile phase gradient generated between 0.01% formic acid in water (mobile phase A) *versus* 0.2% formic acid in methanol (mobile phase B).

### Study samples

All subjects gave written informed consent, and the Institutional Review Board of the Cleveland Clinic approved all study protocols. All human studies performed abided by the Declaration of Helsinki. The content of total protein-bound HCit in subjects with no evidence of CVD, and normal renal function (Table S1), was examined from patient plasma samples obtained from GeneBank at the Cleveland Clinic (clinicaltrials.gov number: NCT00590200), a large and well-characterized single-center cohort with associated tissue repository and longitudinal follow-up clinical database (16, 69, 70, 71). Protein-bound HCit levels were determined from plasma of stable subjects undergoing elective diagnostic cardiac evaluations. Only subjects with normal renal function (estimated glomerular filtration rate >90; calculated using the Chronic Kidney Disease Epidemiology Collaboration creatinine and cystatin C formula method) (72) and without history of diabetes mellitus, coronary artery disease, peripheral artery disease, and significant angiographic evidence of atherosclerosis (<50% stenosis in all major coronary vessels during coronary artery angiography) were included in the present analyses.

### Human aortic tissue sample preparation

Human aortic tissues were obtained as discarded material at time of organ removal from transplant recipients and stored at −80 °C in antioxidant containing buffer under argon atmosphere until use, as previously described (41). Age and gender of subjects from whom human atherosclerotic aortic samples were used in recovery of apoA-I can be found in Table S2. Tissues analyzed in this study, by gross inspection, were morphologically severe atherosclerosis in an advanced disease state (complex and necrotic plaques). Tissue was rinsed in ice-cold normal saline, submerged in argon-sparged 65 mM sodium phosphate buffer (pH 7.4) supplemented with 100 μM diethylenetriamine pentaacetic acid (DTPA) (pH 7.0) and 100 μM butylated hydroxytoluene, and stored at −80 °C in screw cap specimen containers in which headspace was purged with argon. Atherosclerotic tissues were from subjects (n = 10) with an average age of 75 years ± 4 years. Tissues were removed from −80 °C and thawed in sealed container in an ice/water bath. All tissue homogenization procedures were performed at 0 to 4 °C. Wet weight of the aorta was determined, and the tissue was cut into small pieces and then suspended in ice-cold Ca^2+^-free and Mg^2+^-free PBS supplemented with 100 μM DTPA (pH 7.4). A protease inhibitor cocktail (10 μM final; Sigma–Aldrich; catalog no.: P8340) and PMSF (0.5 μM final) were added both initially and in all subsequent solutions used for homogenization and apoA-I isolation. PMSF stock solutions were made in anhydrous ethanol and stored at −20 °C. Aortic tissues were homogenized in ice/water bath with a motor-driven Brinkmann homogenizer for 30 s intervals five times with 2 min rest between homogenizations. Care was taken throughout homogenization to maintain a temperature at or close to 0 °C by keeping the homogenization vessel submerged within slush (ice/water bath). The crude homogenate was centrifuged at a speed of 15,000*g* for 30 min at 0 °C, and the supernatant was used for apoA-I immunoaffinity isolation using cocktail of high-affinity anti-apoA-I antibodies as previously described (16, 41).

### Immunoprecipitation of apoA-I

To quantitatively immunoprecipitate total apoA-I from samples (tissue homogenates or plasma), we used the mAbs 2D10.5 and 10G1.5, which we previously have shown to quantitatively recover apoA-I (including both native and oxidized forms) (41), and a commercially available antihuman apoA-I (LS Bio; catalog number: LS-C316470). mAb 2D10.5 and 10G1.5 hybridoma cells were expanded and used for large-scale antibody production in serum-free media. Antibody was purified using protein A/G (Thermo Scientific Pierce) as described (41). Immunoaffinity resin for apoA-I isolation was generated by covalently coupling mAb 2D10.5, 10G1.5, and commercially available antihuman apoA-I to AminoLink Plus (Pierce Chemical) resin at a density of 1.5 mg antibody per ml of resin in an amine-free buffer using a 1:1:1 ratio of each antibody (PBS, pH 7.4). Unreacted resin sites were blocked using the addition of excess ethanolamine. Antibody flow through was used to determine a crosslinking efficiency of greater than 90%. The resin was washed with 20× volume of 1 M Tris, pH 7.4, 1 M NaCl, and equilibrated with 1× PBS, pH 7.4, prior to immediate usage.

### Proteomics analysis

Protein preparations in 50 mM sodium phosphate buffer, pH 7.0, were digested overnight at 37 °C with MS-grade GluC protease (Promega) at a ratio of 1:20 (w/w) of enzyme to substrate. We noted improved and better peptide coverage and recovery of carbamylated peptides using in-solution digestions. Therefore, we utilized in-solution digestion (with GluC), as the method of choice for sample preparation, unless otherwise indicated. In-gel GluC digestion methodology of excised Coomassie blue–stained immunoaffinity–precipitated apoA-I bands (monomer and dimer) were performed, that is, Coomassie blue–stained gels used for in-gel digestion and downstream HPLC–MS/MS analysis were first excised and then washed/destained overnight with ethanol (50%)/acetic acid (5%) at room temperature. The following day, gel pieces were dehydrated with acetonitrile and dried in a SpeedVac for 6 min. Proteins in the dried bands were reduced with 0.1 ml of 65 mM DTT (Fisher Scientific; catalog no.: BP172–5) at room temperature for 30 min, followed by alkylation for another 30 min at room temperature with 0.1 ml of 162 mM iodoacetamide (Fisher Scientific; catalog no.: AC122270050). Gel pieces were dehydrated two times with 0.2 ml acetonitrile and rehydrated with 0.2 ml of 100 mM ammonium bicarbonate. Next, 40 μl of MS-grade GluC protease (Promega) was added to the dried gel pieces. An additional 20 μl of 50 mM ammonium bicarbonate was added to completely cover the gel pieces and digested overnight at room temperature. For extraction of the protein from the gel pieces, 80 μl of extraction buffer (50% acetonitrile + 5% formic acid) was added twice and incubated for 10 min at room temperature. The gel pieces were microcentrifuged for 10 s, and the supernatants were combined and transferred into a new 0.5 ml tube and dried at room temperature in a Speedvac for 3 h. Finally, 30 μl of 1% acetic acid was added into the dried peptides and transferred into an HPLC vial with cap (Sun-Sri; catalog no.: 200 050 & 501 313) for further mass spectrometric analysis.

Digested peptides were analyzed on a Thermo Fisher Scientific UltiMate 3000 UHPLC system interfaced with a Thermo Fisher Scientific Orbitrap Fusion Lumos Tribrid mass spectrometer or LTQ-Orbitrap Velos, when indicated. Liquid chromatography was performed prior to MS/MS analysis for peptide separation. The HPLC column used is a Thermo Scientific Acclaim PepMap 100 C18 reversed-phase capillary chromatography column (Thermo Fisher Scientific) 75 μm × 15 cm, 2 μm, 100 Å. Five microliters of the peptide extract was injected and peptides eluted from the column by a 110-min acetonitrile/0.1% formic acid gradient at a flow rate of 0.30 μl/min and introduced to the source of the mass spectrometer.

An LTQ-Orbitrap Velos was used for LC–MS/MS analysis of *in vitro* carbamylation specimens and Orbitrap Fusion Lumos Tribrid mass spectrometer was used for analysis of *in vivo* atherosclerotic plaque specimens (Thermo Fisher Scientific). The LTQ-Orbitrap Velos instrument was operated in a data-dependent manner, which involves cycling between an MS1 scan performed in the Orbitrap from 300 to 1700 Da at a resolution of 60,000 followed by MS/MS experiments performed in the ion trap on the 10 most abundant ions. Dynamic exclusion was enabled for ions fragmented twice in 30 s, and these ions were placed on an exclusion list for 60 s.

The Fusion Lumos instrument was operated in a data-dependent manner, which involves cycling between an MS1 scan performed in the Orbitrap from 300 to 1700 Da at a resolution of 120,000 followed by MS/MS experiments performed in the ion trap on the most abundant ions for a cycle time of 3 s. Dynamic exclusion was enabled for ions fragmented once in 30 s, and these ions were placed on an exclusion list for 60 s.

Peak lists were generated with Proteome Discoverer 2.3 (Thermo Fischer Scientific). The resulting Unified Search Files (∗.srf) were searched against the UniProt FASTA of apoA-I using the search parameters given in Table S3. Modifications used for the searches included oxidized methionine and tryptophan (variable), carbamylated lysine (variable mass addition of 43 Da) and acetylated lysine (variable). GluC peptides with a maximum of five missed cleavage sites were allowed in the database searches. Monoisotopic precursor ions were searched with a tolerance of 10 ppm with 0.6 Da for the fragment ions on the data obtained from the hybrid LTQ-Orbitrap Lumos or LTQ-Orbitrap Velos mass spectrometers. Unidentified fragment ions in all fragmentation spectra were manually validated with the use of Protein Prospector (University of California, San Francisco; https://prospector.ucsf.edu/prospector/mshome.htm). All carbamylated peptides presented have MS1 spectra consistent with carbamylation and not acetylation. The peak areas extracted from chromatograms corresponding to each fragmentation spectra were used for quantitation.

### Quantification of site-specific carbamylation

In initial studies, we compared and contrasted three different approaches for quantification of carbamylated peptides: the use of ^15^N-labeled recombinant human apoA-I (rh-apoA-I) as an internal standard, isobaric tagging, and label-free quantitation. We noted that isobaric tagging utilizing a 10-plex isobaric labeling reagent (per the manufacturer instructions) induced a modest but reproducible artifact in our unmodified *in vitro*-generated samples, that either represented artifactual carbamylation, an isobaric modification at the same retention time as carbamylation (*e.g.*, another PTM seen at the same retention time and mass), or isobaric overlap at the MS1 level. The latter would result in observing reporter ions in the MS/MS spectra because of ratio compression/isobaric overlap from the precursor ions (73, 74). The use of ^15^N-labeled recombinant apoA-I worked consistently well for the *in vitro* samples but became problematic when used for *in vivo* immunoprecipitated apoA-I recovered from human atherosclerotic plaque because it was difficult to achieve consistent labeled-to-unlabeled protein molar ratio in all samples following the purification by immunoprecipitation (*i.e.*, much of the sample recovered was either extensively crosslinked or heavily fragmented). On the other hand, the use of relative label-free quantitation proved to be consistent for all *in vitro* and *in vivo* samples (*e.g.*, Table S4; full scan CID mass spectra for the reference apoA-I peptides used are provided in Figs. S23–S26), and our control studies showed that the use of a reference peptide strategy enabled reproducible quantitation of apoA-I peptides within all samples. However, we recognize that differences in ionization efficiencies between target and reference peptides are limitations of this approach, providing only semiquantitative data on peptides. Nevertheless, in this study, protein-bound carbamylation was quantified using the previously described proteomic relative label-free quantitation methodology (75, 76).

Carbamylation peptide quantitation data are displayed as the peak area of carbamylated lysine containing peptide divided by the peak area of an endogenous apoA-I reference peptide multiplied by 10^4^. For any carbamylated lysine residue that was identified in multiple different peptides, the areas of all peptides harboring the carbamylated residue were combined. Initially, we sought to evaluate the reproducibility of this method by using multiple different reference peptides as the denominator. The criteria for selecting reference peptide candidates were the following: (1) the reference peptide should not contain amino acid residues potentially modifiable by the reagents used for carbamylation (*e.g.*, lysine residues and if possible, methionine, histidine, or other nucleophilic or readily oxidizable residue); (2) the reference peptide should be present and highly abundant in all sample replicates; (3) the reference peptide should be present in all samples from analysis groups (both *in vitro* carbamylated and apoA-I recovered from human atherosclerotic aortic plaque). Similar results were generated when relative quantification was presented using either the top three reference peptides or a weighted average of the three endogenous reference peptides (*e.g.*, Table S4 and Figs. S3 and S4); however, in the end, we selected the reference peptide D_213_–E_223_ (reference peptide 1) to use as denominator for peptide quantification because it was detected with high confidence in all samples examined (both *in vitro* and *in vivo* (*e.g.*, Table S4 and Figs. S3 and S4)).

### Production of rh-apoA-I

rh-apoA-I protein was expressed from endotoxin-free *Escherichia coli* (Clear Coli) with removal of the His tag, as previously described (77, 78). The protein was purified using nickel–nitrilotriacetic acid chromatography (78). Care was taken during all steps to avoid use of urea during the isolation procedure. Purified apoA-I was stored at −80 °C under argon atmosphere in 6 M guanidinium hydrochloride until use. Upon usage, apoA-I was thawed, extensively dialyzed in PBS (pH 7.0), supplemented with 100 μM DTPA (added to PBS from 1000× pH 7.0 stock) to remove any trace of redox-active metals. The purity of recombinant apoA-I protein was determined by 12% reducing SDS-PAGE and Coomassie blue staining.

### Preparation and purification of rHDL

rHDL was prepared from purified rh-apoA-I using the Jonas sodium cholate dialysis method with a molar ratio of apoA-I:1-palmitoyl-2-oleoyl-*sn*-glycero-3-phosphocholine:cholesterol of 1:100:10 (79). rHDL particles were purified by gel filtration chromatography with the use of a Sephacryl S300 column (GE Healthcare) on a Bio-Rad Biologics DuoFlo FPLC (Bio-Rad), as previously described (11). The size of rHDL containing apoA-I was estimated from 4 to 20% nondenaturing equilibrium gel electrophoresis (using precast gels; Bio-Rad) by comparison with protein standards of known Stokes diameter (GE Healthcare) as previously described (77). Nondenaturing gels were then analyzed by Coomassie blue staining. The amount of cholesterol was determined using a LiquiColor enzymatic cholesterol test kit (Stanbio Laboratory). Total phospholipid content in the rHDL was quantified by microphosphorous assay (80).

### *In vitro* carbamylation of apoA-I and rHDL

Protein (either in lipid-poor form or within an HDL particle) was subjected to *in vitro* carbamylation by one of two methods: (1) carbamylation with the complete MPO/H_2_O_2_/SCN^-^ system (30 nM MPO, 100 μM SCN^−^) using a molar ratio of 10 mol of H_2_O_2_ to 1 mol of apoA-I (apoA-I molecular weight used for calculations = 28,300 kDa) and (2) carbamylation by CNO^−^ carried out using 10 mol of KCNO for each mol of apoA-I, in 50 mM sodium phosphate (pH 7.0), at 37 °C. Following carbamylation, samples were extensively dialyzed with multiple buffer exchanges at 4 °C against 1× PBS supplemented with 100 μM DTPA (pH 7.4); DTPA was not added in the final dialysis round.

### SPR analysis

The kinetic constants *k*_on_, *k*_off_ and the apparent equilibrium dissociation constant (*K*_*D*_) for purified human MPO binding to purified lipid-poor apoA-I were determined using SPR spectroscopy (BIAcore 3000 SPR biosensor; BIAcore, AB). Briefly, MPO was immobilized on a CM5 sensor chip through primary amino groups using the manufacturer's directions and as previously described (77). Lipid-poor apoA-I was captured on the sensor chip through interaction with immobilized MPO by injecting apoA-I at a flow rate of 15 μl/min in 10 mM PBS buffer (pH 7.4) into the flow cell. To determine the *K*_*D*_, varying concentrations of apoA-I (0.5 nM to 3 μM) were flowed over immobilized MPO in binding buffer (10 mM PBS, pH 7.4) at a flow rate of 20 μl/min. The apparent *K*_*D*_ was obtained by fitting background-subtracted SPR-binding data to the 1:1 binding with a drifting baseline model in the BIAevaluation program, version 4.0 (BIAcore, AB).

### Preparation of cross-linked protein samples

ApoA-I and MPO (1:1, mol:mol) were crosslinked using 300-fold molar excess of the DSSO crosslinker for 1 h at room temperature, 100 mM sodium phosphate, 150 mM NaCl, pH 7.4. The reaction was quenched using 20 mM Tris–HCl (pH 8.0). Protein cross-linked samples were then fractionated on a reducing 12% SDS-PAGE gel and stained with Coomassie blue to detect protein bands.

### Statistical analysis

We used Student’s *t* test (2-tailed) to report differences between the MPO and CNO^−^ system. However, we used Wilcoxon rank sum test to report the differences between total protein-bound HCit and protein-bound HCit in apoA-I isolated lesions in Figure 2 and differences between monomer *versus* dimer in Figure 7. *p* Values for statistical significance are reported for *p* ≤ 0.05. All data are presented as mean ± SD. Statistical tests used to compare conditions are indicated in figure legends. GraphPad Prism (GraphPad Software, Inc), version 9.0 and R (R Core Team), version 3.4.2 (2017) were used for generation of graphs and statistics.

## Data availability

The MS raw data were deposited to the ProteomeXchange Consortium (PRIDE repository) (81) and can be accessed with the dataset identifier PXD027881. PRIDE accessible raw file names for *in vitro*-modified apoA-I and apoA-I isolated from human lesion samples used in the proteomics analysis can be found in Table S12.

## Supporting information

This article contains supporting information.

## Conflict of interest

S. L. H. reports being named as coinventor on pending and issued patents held by the Cleveland Clinic relating to cardiovascular diagnostics and therapeutics, being a paid consultant for 10.13039/100004357Procter & Gamble and Zehna Therapeutics, having received research funds from 10.13039/100004357Procter & Gamble, Zehna Therapeutics, and 10.13039/100016545Roche Diagnostics, and being eligible to receive royalty payments for inventions or discoveries related to cardiovascular diagnostics or therapeutics from Cleveland HeartLab, a wholly owned subsidiary of 10.13039/100015627Quest Diagnostics, 10.13039/100004357Procter & Gamble, and Zehna Therapeutics. All the other authors declare that they have no conflicts of interest with the contents of this article.
