# Supplementary material for: The pattern of apolipoprotein A-I lysine carbamylation reflects its lipidation state and the chemical environment within human atherosclerotic aorta
Source: J Biol Chem. 2022 Mar 15;298(4):101832. doi: 10.1016/j.jbc.2022.101832 (PMC9010765; doi:10.1016/j.jbc.2022.101832)
Supplement: Supporting information [file mmc1.pdf]

Supporting Information

The pattern of apolipoprotein A-I lysine carbamylation reflects its lipidation state and the chemical environment within human atherosclerotic aorta

Shawna Battle[1,2], Valentin Gogonea[1,3], Belinda Willard[4], Zeneng Wang[1,2], Xiaoming Fu[1], Ying Huang[1], Linda M. Graham[2,5,6], Scott J. Cameron[1,2,6,7], Joseph A. DiDonato[1,2], John W. Crabb[2,8], and Stanley L. Hazen[1,2,3,6]

[1] Department of Cardiovascular & Metabolic Sciences, Cleveland Clinic, Cleveland, OH
[2] Cleveland Clinic Lerner College of Medicine, Case Western Reserve University, Cleveland, OH
[3] Department of Chemistry, Cleveland State University, Cleveland, OH
[4] Proteomics Shared Laboratory Resource, Cleveland Clinic, Cleveland, OH
[5] Department of Biomedical Engineering, Lerner Research Institute, Cleveland Clinic, Cleveland, OH, USA
[6] Heart Vascular and Thoracic Institute, Cleveland Clinic, Cleveland, OH
[7] Taussig Cancer Center, Cleveland Clinic, Cleveland, OH
[8] Cole Eye Institute, Cleveland Clinic, Cleveland, OH

Address correspondence to:
Stanley Hazen, Dept. of Cardiovascular and Metabolic Sciences/NC10, Lerner Research Institute, Cleveland Clinic, 9500 Euclid Ave., Cleveland, OH 44195. Tel.: 216-445-9763; Fax: 216-444-9404; E-mail: hazens@ccf.org.

Table of Contents

Figure/ Table	Title	Page
Table S1	Demographics and laboratory values of Clinical Cohort used in the analysis of protein bound HCit content in plasma	3
Table S2	Age and gender of subjects from whom human atherosclerotic aortic samples were used in recovery of apoA-I for proteomic analysis	4
Table S3	Peptide search parameters for Proteome Discoverer program	5
Table S4	Abundance and frequencies of detection of reference peptide candidates used in the proteomic analysis	6 - 7
Table S5	*In vitro* CNO- modified rHDL/apoA-I peptides containing carbamylated lysine residues detected in the proteomic analyses	8
Table S6	*In vitro* MPO modified rHDL / apoA-I peptides containing carbamylated lysine residues detected in the proteomic analysis	9
Table S7	*In vitro* CNO^- modified lipid poor apoA-I peptides containing carbamylated lysine residues detected in the proteomic analysis	10
Table S8	*In vitro* MPO modified lipid poor apoA-I peptides containing carbamylated lysine residues detected in the proteomic analysis	11
Table S9	Human atherosclerotic aorta apoA-I peptides containing carbamylated lysine residues detected by the proteomic analysis	12-13
Table S10	Xcorr Sequest scores for Human atherosclerotic lesion apoA-I peptides containing carbamylated lysine residues detected during proteomic analyses	14 - 15
Table S11	Kinetic and binding affinity data for lipid poor apoA-I binding to MPO determined by Surface Plasmon Resonance spectroscopy	16
Table S12	PRIDE accessible raw file names for *in- vitro* modified apoA-I and apoA-I isolated from human lesion samples used in the proteomics analysis	17-19
Figure S1	Characterization of rHDL particles	19
Figure S2	Western blot analysis of lipid poor apoA-I crosslinked with MPO	20
Figure S3	Quantification of carbamylated lysine residues in lipid poor apoA-I modified *in vitro* by the MPO or CNO^- system	21
Figure S4	Quantification of site-specific carbamylation of apoA-I recovered from human atherosclerotic aorta	22
Figure S5	Lysine carbamylation sites identified by the proteomic analysis of rHDL and lipid poor apoA-I modified *in vitro* by CNO^-	23
Figure S6-S22	Annotated MS/MS spectra, table of fragmentation ions, and table indicating where detected (*in vivo* and *in vitro* samples) for each human apoA-I peptide detected harboring a carbamyllysine residue	24-40
Figure S23-S26	Annotated MS/MS spectra and tables of fragmentation ions for apoA-I reference peptides	41-44

Table S1. Demographics and laboratory values of the clinical cohort subset (GeneBank, GB) used in the analysis of protein bound HCit content in plasma.

Characteristics	GB subset (n=117)
Age (yr)	60.7(55.6-66.5)
Sex (% male)	45.6
SBP (mmHg)	131.0(120.0-144.0)
HgbA1C (%)	5.5(5.2-5.9)
HDLc (mg/dL)	38.8(32.0-48.0)
LDLc (mg/dL)	105.0(87.0-123.0)
Cholesterol (mg/dL)	171.4(151.9-194.3)
Triglyceride (mg/dL)	97.5(77.3-140.8)
eGFR (mL/min/1.73 m^2)	80.4(70.3-91.4)
CRP (mg/dL)	1.74(0.86-4.57)

Clinical and laboratory characteristics of GB subjects (n=117) for whom protein-bound HCit was quantified in plasma proteins. Subjects included were confirmed to be without known cardiovascular or metabolic diseases, and with normal renal function, as described under Experimental Procedures. Plasma was analyzed for protein bound HCit content using stable isotope dilution LC/MS/MS methods as described. Values shown are median (interquartile range) for continuous variables, and percent for dichotomous variables.

Abbreviations: Systolic blood pressure (SBP), hemoglobinA1C (HgbA1C), high density lipoprotein cholesterol (HDLc), low density lipoprotein cholesterol (LDLc), estimated glomerular filtration rate (eGFR), C-reactive protein (CRP)

Table S2. Age and gender of subjects from whom human atherosclerotic aortic samples were used in recovery of apoA-I for proteomic analysis.

Human aorta sample	Age	Gender	Sequence coverage %
1	79	Male	93
2	76	Female	93
3	74	Male	93
4	81	Male	90
5	76	Male	90
6	75	Female	93
7	73	Female	92
8	68	Female	90
9	82	Female	90
10	71	Male	90

Human aortic tissue was obtained as discarded material at time of organ removal from transplant recipients. Atherosclerotic donor tissues were from subjects (n=10) of average age 75yr +/- 4yr. Both males and females were analyzed. ApoA-I was immunoprecipitated from aortic tissue homogenates using a cocktail of anti-apoA-I antibodies using methods shown to result in quantitative recovery of total apoA-I from samples, as described under Experimental Procedures. Recovered apoA-I protein was subjected to proteolysis with GluC and mass spectrometry was performed as described under Experimental Procedures. Proteome Discoverer 2.3 was used to identify peptide sequences and % coverage.

Table S3. Peptide search parameters for Proteome Discoverer.

**Protein Database: ApoA1_His.fasta**
- Enzyme Name: GluC
- Maximum Missed Cleavage Sites: 5
**Tolerances:**
- Precursor Mass Tolerance: 10 ppm
- Fragment Mass Tolerance: 0.6 Da
- Max. Identical Dynamic Modifications Per Peptide: 3
- Max. Dynamic Modifications Per Peptide: 4
**Fixed Value PSM Validator:**
Maximum Delta Cn: 0.05
Maximum Rank: 0
**Protein FDR Validator:**
Target FDR (Strict): 0.01
Target FDR (Relaxed): 0.05
**Dynamic Modification**:
- Oxidation / +15.995 Da (M, W)
- Carbamylation / +43.006 Da (K)
- Acetylation / +42.001 Da (K)

Peak lists were generated with Proteome Discoverer 2.3 (Thermo Fischer Scientific, Waltham, MA) using the parameters listed. The resulting Unified Search Files (*.srf) were searched against the Uniprot FASTA of human apoA-I. Modifications used for the searches included, oxidized methionine (variable), oxidized tryptophan (variable), carbamylated lysine (variable, mass addition 43 Da) and acetylated lysine (variable). GluC peptides with a maximum of 5 missed cleavage sites were allowed in the database searches. Monoisotopic precursor ions were searched with a tolerance of 10 ppm with 0.6 Da for the fragment ions.

Table S4. Abundance and frequency of detection of reference peptide candidates used in the proteomic analysis.

Reference peptide candidate		Sample									
		rHDL+MPO		rHDL+CNO^-		apoA-I+MPO		apoA-I+CNO^-		Human plaque	
		RPA (%)	DFRP (%)	RPA (%)	DFRP (%)	RPA (%)	DFRP (%)	RPA (%)	DFRP (%)	RPA (%)	DFRP (%)
Cluster 1 of reference peptide candidates											
1A	^{213}DLRQGLLPVLE^{223}	99±0.2	100	66±12	100	82±0.5	100	87±1	100	98±0.4	100
1B	^{206}KAKPALEDLRQGLLPVLE^{223}	1±0.2	100	22±6	100	14±0.9	100	11±1	100	2±0.4	100
1C	^{199}HLSTLSEKAKPALEDLRQGLLPVLE^{223}	<0.001	100	12±6	100	4±0.4	100	2±0.1	100	<0.1±.01	80
Cluster 2 of reference peptide candidates											
2A	^{79}TEGLRQE^{85}	100±0	100	100±0	100	4±0.3	100	6±0.5	67	79±15	100
2B	^{81}GLRQEMSKDLE^{91}	<0.001	100	<0.001	100	96±0.3	100	94±0.5	100	9±5.6	100
2C	^{79}TEGLRQEMSKD^{89}	<0.001	100	<0.001	100	<0.001	100	<0.001	100	12±13	100
Cluster 3 of reference peptide candidates											
3A	^{184}NGGARLAE^{191}	<0.1±0.1	100	0.3±0.1	100	11±4.6	100	35±1.3	67	68±16	100
3B	^{180}ALKENGGARLAE^{191}	99±0.1	100	99±0.1	100	89±4.6	100	65±1.2	67	32±16	100
Cluster 4 of reference peptide candidates											
4A	^{63}QLGPVTQE^{70}	24±30	100	26±24	67	62±3.5	100	21±2	100	99±0.1	100

4B	^{63}QLGPVTQEFWDNLEKE^{78}	<0.001	100	17±15	100	1.0±0.1	100	<0.001	100	<0.1±0.1	70
4C	^{35}GSALGKQLNLKLLDNWDSVTSTFSKLREQLGPVTQEFWDNLEKE^{78}	76±30	100	65±11	100	37±3.5	100	79±2	100	0.5±0.6	90
Cluster 5 of reference peptide candidates											
5A	^{71}FWDNLE^{76}	-	-	-	-	1±0.1	100	1±0.1	100	-	-
5B	^{71}FWDNLEKE^{78}	-	-	-	-	56±0.4	100	55±0.9	100	-	-
5C	^{71}FWDNLEKETE^{80}	-	-	-	-	43±0.5	100	44±1	100	-	-

To select optimal apoA-I peptides that might serve as an internal reference peptide for relative quantification of carbamyllysine harboring apoA-I peptides in samples, we used the following criteria: 1) the reference peptide candidate doesn't contain lysine residues and if possible, methionine, histidine, or other nucleophilic or oxidizable residues; 2) the reference peptide candidate should be readily detectable in all sample replicates; 3) the reference peptide candidate should be readily detectable in all samples from all analyses groups (i.e. rHDL + MPO; rHDL + CNO^- ; apoA-I + MPO; apoA-I + CNO^-; and Human plaque).

RPA (%) = average reference peptide candidate abundance ± standard deviation = 100 X (peak area of reference peptide candidates / (sum of peak areas of reference peptide candidates in the same peptide cluster, (e.g. peptides 1A, 1B, 1C from reference peptide candidates cluster 1)).

DFRP (%) = detection frequency of reference peptide candidate = 100 x (number of times reference peptide candidate was observed in replicate samples / total of replicate samples). The DFRP is listed in bold face if the reference peptide was not seen in all replicates and therefore its priority for use was reduced.

(-) indicates reference peptide candidate not used in analysis.

We observed similar qualitative results, whether using three different single reference peptides, vs. a weighted average of the top three performing reference peptides (e.g. see Figure S3, and S4). However, based on the data analyses shown above, we also noted that only reference peptide D_{213}-E_{223} showed optimal characteristics (i.e. it was the only reference peptide observed in all *in vitro* and *in vivo* samples). We therefore elected to use this internal reference peptide as the denominator for all apoA-I quantifications.

Table S5. *In vitro* CNO^- modified rHDL/apoA-I peptides containing carbamylated lysine residues detected in the proteomic analyses

Peptide Sequence	m/z	Charge	Δm (ppm)	Xcorr Sequest Score
GSALGKQLNLK_{45}LLDNWDSVTSTFSKLRE	791.67	4	-0.2	5.89
VK_{94}AKVQPYLDDFQKKWQEE	808.08	3	-1.58	3.04
MELYRQK_{118}VEPLRAELQE	725.71	3	0.08	3.22
LYRQK_{118}VEPLRAE	515.62	3	-0.32	2.91
LYRQK_{118}VEPLRAELQE	639.01	3	0.03	3.4
ALK_{182}ENGGARLAE	636.33	2	-0.5	3.14
SFK_{226}VSFLSALEEYTKKLNTQ	792.75	3	1.1	4.95
SFK_{226}VSFLSALEE	700.35	2	0.09	3.98
YTKK_{239}LNTQ	519.78	2	0.19	2.44
GSALGK_{40}QLNLK_{45}LLDNWDSVTSTFSK_{59} LREQLGPVTQE	1004.78	4	0.44	5.14
Reference peptide candidates				
^{213}DLRQGLLPVLE^{223}	626.86	2	-0.85	2.84
^{79}TEGLRQE^{85}	416.71	2	0.32	2.82
^{184}NGGARLAE^{191}	394.20	2	-0.86	3.03

LC-MS/MS analysis of GluC digests from *in vitro* modified rHDL (CNO^-) preparations yielded identification of ≥ 90% of apoA-I protein sequence (Table S2). Peptide sequence, m/z, Δm, charge and Xcorr produced by Proteome Discoverer for the *in vitro* CNO^- modified rHDL/apoA-I peptides containing carbamylated lysine residues included in the proteomic analysis are listed above.

Carbamylated lysine residue within the peptide sequence is shown in red.

m/z = the ratio of the mass (Da) and the charge (a.u.) of the digested peptide.

Δm = mass difference (ppm) between measured peptide and its theoretical mass.

Charge (positive ion mode) = peptide charge (a.u.).

Xcorr (Proteome Discoverer) = cross-correlation value from the search.

Table S6. ***In vitro*** **MPO modified rHDL/apoA-I peptides containing carbamylated lysine residues detected in the proteomic analysis.**

Peptide Sequence	m/z	Charge	Δm (ppm)	Xcorr Sequest Score
LATVYVDVLK_{23}DSGRDYVSQFE	816.40	3	-2.07	4.82
GSALGK_{40}QLNLKLLDNWDSVTSTFSKLRE	791.67	4	-0.2	6.38
VK_{94}AKVQPYLDDFQKKWQEE	808.08	3	-1.58	3.7
LYRQK_{118}VEPLRAE	515.62	3	3.83	2.88
YHAK_{195}ATEHLSTLSEKAKP	524.77	4	-0.02	1.78
SFK_{226}VSFLSALEE	700.35	2	-2.53	4.43
YTK_{238}KLNTQ	519.78	2	-0.99	2.71
Reference peptide candidates				
^{213}DLRQGLLPVLE^{223}	626.86	2	-0.85	3.03
^{79}TEGLRQE^{85}	416.71	2	0.32	2.48
^{184}NGGARLAE^{191}	394.20	2	-0.86	2.95

LC-MS/MS analysis of GluC digests from *in vitro* modified rHDL (MPO) preparations yielded identification of ≥ 90% of apoA-I protein sequence. Peptide sequence, m/z, Δm, charge and Xcorr produced by Proteome Discoverer are listed above.

Carbamylated lysine residue is shown in red.

m/z = the ratio of the mass (Da) and the charge (a.u.) of the digested peptide.

Δm = mass difference (ppm) between measured peptide and its theoretical mass.

Charge (positive ion mode) = peptide charge (a.u.).

Xcorr (Proteome Discoverer) = cross-correlation value from the search.

Table S7. *In vitro* CNO⁻ modified lipid poor apoA-I peptides containing carbamylated lysine residues detected in the proteomic analysis.

Peptide Sequence	m/z	Charge	Δm (ppm)	Xcorr Sequest Score
LATVYVDVLK$_{23}$DSGRDYVSQFE	816.40	3	-2.07	3.05
GSALGK$_{40}$QLNLKLLDNWDSVTSTFSKLREQ LGPVTQE	1004.78	4	-0.59	5.07
VK$_{94}$AKVQPYLDDFQKKWQEE	808.08	3	-1.58	4.58
LYRQK$_{118}$VEPLRAELQE	639.01	3	-1.02	3.87
LYRQK$_{118}$VEPLRAE	515.62	3	3.83	3.06
YHAK$_{195}$ATEHLSTLSE	543.93	3	-2.22	1.44
SFK$_{226}$VSFLSALEE	700.35	2	-2.53	3.58
VKAK$_{96}$VQPYLDDFQK$_{106}$K$_{107}$WQEE	836.76	3	8.17	1.22
GSALGK$_{40}$QLNLKLLDNWDSVTSTFSK$_{59}$ LRE	1055.22	3	-1.37	7.01
Reference peptide candidates				
^{213}DLRQGLLPVLE^{223}	626.86	2	-0.85	2.85
^{63}QLGPVTQE^{70}	436.22	2	-1.78	2.5
^{71}FWDNLE^{78}	823.36	1	-0.86	1.87

LC-MS/MS analysis of GluC digests from *in vitro* CNO⁻ modified lipid poor apoA-I preparations yielded identification of ≥ 90% of apoA-I protein sequence. Peptide sequence, m/z, Δm, charge and Xcorr produced by Proteome Discoverer are listed above.

Carbamylated lysine residue is shown in red.

m/z = the ratio of the mass (Da) and the charge (a.u.) of the digested peptide.

Δm = mass difference (ppm) between measured peptide and its theoretical mass.

Charge (positive ion mode) = peptide charge (a.u.).

Xcorr (Proteome Discoverer) = cross-correlation value from the search.

Table S8. ***In vitro*** **MPO modified lipid poor apoA-I peptides containing carbamylated lysine residues detected in the proteomic analysis.**

Peptide Sequence	m/z	charge	Δm (ppm)	Xcorr Sequest Score
GSALGK_{40}QLNLKLLDNWDSVTSTFSKLRE	791.67	4	0.03	6.31
GSALG K_{40}QLNLKLLDNWDSVTSTFSKLREQLGPVTQE	1004.78	4	-0.59	4.83
LYRQK_{118}VEPLRAE	515.62	3	-3.17	2.88
YHAK_{195}ATEHLSTLSE	543.93	3	-1.65	1.24
SGRDYVSQFEGSALGK_{40}QLNLK_{45}LLDNWD	1047.18	3	2.67	1.48
Reference peptide candidates				
^{213}DLRQGLLPVLE^{223}	626.86	2	-0.85	2.94
^{63}QLGPVTQE^{70}	436.22	2	-1.78	2.51
^{71}FWDNLE^{78}	823.36	1	-0.86	1.85

LC-MS/MS analysis of GluC digests from *in vitro* modified (MPO) lipid poor apoA-I preparations yielded identification of ≥ 90% of apoA-I protein sequence. Peptide sequence, m/z, Δm, charge and Xcorr produced by Proteome Discoverer are listed above.

Carbamylated lysine residue is shown in red.

m/z = the ratio of the mass (Da) and the charge (a.u.) of the digested peptide.

Δm = mass difference (ppm) between measured peptide and its theoretical mass.

Charge (positive ion mode) = peptide charge (a.u.).

Xcorr (Proteome Discoverer) = cross-correlation value from the search.

Table S9. Human atherosclerotic aorta apoA-I peptides containing carbamylated lysine residues detected by the proteomic analysis.

Peptide Sequence	m/z	Charge	Δm (ppm)
DEPPQSPWDRVK_{12}D	537.92	3	-2.65
LATVYYVDVLK_{23}D	639.85	2	-1.41
VLK_{23}DSGRDYVSQFE	843.41	2	-4
VYVDVLK_{23}DSGRDYVSQFE	1081.5	2	1.15
LATVYYVDVLK_{23}DSGRDYVSQFE	816.41	3	0.1
GSALGK_{40}QLN	465.75	2	-2.0
GSALGK_{40}QLNLK	586.34	2	0.64
ALGK_{40}QLNLKLLDNWDSVTSTFSKLRE	766.41	4	-5.44
SALGK_{40}QLNLKLLDNWDSVTSTFSKLRE	1036.2	3	-3.33
GSALGK_{40}QLNLKLLD	504.96	3	-2.19
GSALGKQLNLK_{45}LLDNWDSVTS	1151.6	2	-0.89
LNLK_{45}LLDNWDSVTSTFSKLRE	841.45	3	0.63
QFEGSALGKQLNLK_{45}LLDNWDSVTSTFSKLRE	714.38	5	1.16
WDSVTSTFSK_{59}LRE	799.89	2	-1.97
NWDSVTSTFSK_{59}LRE	856.91	2	-0.96
LKLLDNWDSVTSTFSK_{59}LRE	765.74	3	1.03
NLKLLDNWDSVTSTFSK_{59}LRE	803.75	3	-3.54
GSALGKQLNLKLLDNWDSVTSTFSK_{59}L	960.18	3	0.20
FWDNLEK_{77}ETE	677.3	2	-1.16
GLRQEMSK_{88}DLEE	747.35	2	-0.78
TEGLRQEMSK_{88}DLEE	862.39	2	-0.71
VK_{94}AKVQPYLD	602.34	2	-2.07
VK_{94}AKVQPYLDD	659.85	2	-2.05
VKAK_{96}VQPYLD	602.34	2	0.97
FQKK_{107}WQEE	583.28	2	-1.36
YRQK_{118}VEPLRAE	477.93	3	-1.53
LYRQK_{118}VEPLRAE	515.62	3	-2.45
MELYRQK_{118}VEPLRAE	602.32	3	-1.84
MELYRQK_{118}VEPLRAELQE	725.71	3	-3.2
LQEK_{140}LSPLGEE	643.33	2	-0.60
K_{140}LSPLGEEMRD	667.32	2	-1.82
ALK_{182}ENGGARLAE	636.33	2	-7.60
KAK_{208}PALE	400.24	2	-3.08
SFK_{226}VSFL	435.74	2	-1.13
SFK_{226}VSFLSA	514.77	2	0.54

Peptide Sequence (Table S9 Continued)	m/z	charge	Δm (ppm)
SFK_{226}VSFLSAL	571.32	2	2.23
K_{226}VSFLSALEE	583.31	2	-1.17
SFK_{226}VSFLSALE	635.84	2	-1.64
FK_{226}VSFLSALEE	656.84	2	-0.99
SFK_{226}VSFLSALEE	700.36	2	-0.61
YTKK_{239}LNT	455.75	2	0.53
YTKK_{239}LNTQ	519.78	2	-1.81
K_{40}QLNLK_{45}LLDNWDSVTSTFSKLRE	706.12	4	-2.25
SALGK_{40}QLNLK_{45}LLDNWDSVTSTFSKLRE	788.17	4	-0.5
GSALGK_{40}QLNLK_{45}LLDNWDSVTSTF	850.78	3	-0.71
GSALGK_{40}QLNLK_{45}LLDNWDSVTSTFSK_{59}LRE	791.68	4	1.81
VK_{94}AK_{96}VQPYLDDFQ$K_{106}$$K_{107}$WQEE	808.08	3	-0.90
EVK_{94}AK_{96}VQPYLDDFQ $K_{106}$$K_{107}$WQEE	851.1	3	-0.69
Reference peptide candidates			
^{63}QLGPVTQE^{70}	436.21	2	1.06
^{213}DLRQGLLPVLE^{223}	626.86	2	-0.33
^{79}TEGLRQE^{85}	416.72	2	-1

LC MS/MS analysis of GluC peptide digests generated from apoA-I isolated from human atherosclerotic aorta preparations yielded coverage of ≥ 90% of apoA-I protein sequence. Peptide sequence, m/z, Δm and charge are listed in the table above.

Carbamylated lysine residue is shown in red

m/z = the ratio of the mass (Da) and the charge (a.u.) of the digested peptide

Δm = mass difference (ppm) between measured peptide and its theoretical mass

Charge (positive ion mode) = peptide charge (a.u.)

Table S10. Xcorr Sequest scores for human atherosclerotic lesion apoA-I peptides containing carbamylated lysine residues detected during proteomic analyses.

	Human subject aorta carbamylated apoA-I peptide Xcorr Values (n=10)									
	1	2	3	4	5	6	7	8	9	10
Peptide Sequence										
DEPPQSPWDRVK_{12}D	2.16	2.38	2.26	2.33	2.65	2.26	2.38	2.32	2.93	2.11
LATVYVDVLK_{23}D	-	-	-	-	-	2.39	-	-	-	-
VLK_{23}DSGRDYVSQFE	-	2.46	-	-	3.18	2.01	-	2.26	2.25	2.63
VYVDVLK_{23}DSGRDYVSQFE	-	-	2.46	-	-	-	-	-	-	-
LATVYVDVLK_{23}DSGRDYVSQFE	3.71	3.31	-	3.31	3.72	4.04	3.57	3.65	3.4	3.73
GSALGK_{40}QLN	2.71	2.15	-	2.61	2.74	2.27	2.64	2.72	2.9	-
GSALGK_{40}QLNLK	-	2.62	-	-	-	-	-	-	-	-
ALGK_{40}QLNLKLLDNWDSVTSTFSKLRE	4.41	-	-	-	-	3.54	4.29	-	4.88	5.02
SALGK_{40}QLNLKLLDNWDSVTSTFSKLRE	4.22	-	4.8	-	4.98	-	-	-	-	-
GSALGK_{40}QLNLKLLD	2.85	3.11	-	-	-	2.64	2.69	2.03	2.56	-
GSALGKQLNLK_{45}LLDNWDSVTS	-	-	-	-	-	-	2.85	-	3.26	3.31
LNLK_{45}LLDNWDSVTSTFSKLRE	-	3.52	-	-	-	-	-	-	-	-
QFEGSALGKQLNLK_{45}LLDNWDSVTSTFSKLRE	-	-	-	-	-	-	3	-	-	-
WDSVTSTFSK_{59}LRE	2.16	-	3.05	2.78	2.79	3.58	2.97	-	-	-
NWDSVTSTFSK_{59}LRE	4.27	3.48	3.61	4.38	4.59	3.56	4.39	3.71	3.7	3.94
LKLLDNWDSVTSTFSK_{59}LRE	4.16	4.32	4.91	4.3	4.29	3.76	3.86	4.21	4.07	4.11
NLKLLDNWDSVTSTFSK_{59}LRE	-	3.19	-	-	-	-	-	2.93	-	-
GSALGKQLNLKLLDNWDSVTSTFSK_{59}L	-	3.2	-	-	-	-	-	-	-	-
FWDNLEK_{77}ETE	-	-	-	2.54	2.18	2.13	2.44	-	-	2.4
GLRQEMSK_{88}DLEE	-	2.5	-	2.61	2.29	-	2.59	2.57	2.63	-
TEGLRQEMSK_{88}DLEE	-	-	-	-	-	2.81	-	2.23	3.1	3.05
VK_{94}AKVQPYLD	-	-	-	-	-	2.13	-	-	-	-
VK_{94}AKVQPYLDD	-	-	2.44	2.33	2.13	-	2.11	-	-	2.41
VKAK_{96}VQPYLD	-	-	-	2.44	-	-	-	-	-	-
FQKK_{107}WQEE	-	-	2.05	-	-	-	-	-	-	-
YRQK_{118}VEPLRAE	-	2.69	-	-	2.63	-	-	-	-	-
LYRQK_{118}VEPLRAE	2.98	-	2.69	3.54	2.93	2.84	3.38	2.68	3.06	2.72
MELYRQK_{118}VEPLRAE	-	-	2.89	3.22	3.7	3.12	-	-	2.78	2.5
MELYRQK_{118}VEPLRAELQE	-	-	-	-	-	2.52	-	-	-	-
LQEK_{140}LSPLGEE	-	-	-	-	-	-	-	-	2.33	-

Peptide Sequence (Table S10 Continued)	1	2	3	4	5	6	7	8	9	10
K$_{140}$LSPLGEEMRD	2.12	-	-	-	-	2.49	2.22	-	2.01	-
ALK$_{182}$ENGGARLAE	1.9	2.3	2.1		2.5	2.7	1.2			2.7
KAK$_{208}$PALE	2.2	2.1	-	-	-	-	2	-	2.05	-
SFK$_{226}$VSFL	-	2.2	2.13	-	-	-	-	-	-	-
SFK$_{226}$VSFLSA	-	-	2.48	-	-	-	-	-	-	-
SFK$_{226}$VSFLSAL	-	2.16	-	-	-	-	-	-	-	-
K$_{226}$VSFLSALEE	-	-	-	-	-	-	-	-	-	2.15
SFK$_{226}$VSFLSALE	-	2.66	2.36	-	-	2.61	2.8	-	-	-
FK$_{226}$VSFLSALEE	-	-	-	-	-	-	2.09	-	-	-
SFK$_{226}$VSFLSALEE	4.58	3.9	4.1	4.33	4.51	4.51	3.85	3.49	3.87	3.52
YTKK$_{239}$LNT	2.04	-	-	-	-	-	-	-	-	-
YTKK$_{239}$LNTQ	-	2.6	2.22	2.48	2.46	2.45	2.46	2.44	2.61	2.31
K$_{40}$QLNLK$_{45}$LLDNWDSVTSTF SKLRE	5.68	4.4	4.89	-	5.84	4.65	-	-	-	3.32
SALGK$_{40}$QLNLK$_{45}$LLDNWDSV TSTFSKLRE	-	-	-	-	-	3.72	3.92	-	-	-
GSALGK$_{40}$QLNLK$_{45}$LLDNWDS VTSTF	-	-	-	-	2.99	-	-	-	-	-
GSALGK$_{40}$QLNLK$_{45}$LLDNWDS VTSTFSK$_{59}$LRE	5.85	5.91	6.11	5.26	-	5.04	-	5.64	5.74	5.42
VK$_{94}$AK$_{96}$VQPYLDDFQK$_{106}$K$_{107}$WQEE	4.39	4.74	3.51	-	4.08	-	3.91	4.42	-	4.09
EVK$_{94}$AK$_{96}$VQPYLDDFQ K$_{106}$K$_{107}$WQEE	-	-	3.85	2.75	2.65	2.84	3.33	-	2.69	-
Reference peptide candidates										
^{213}DLRQGLLPVLE^{223}	2.55	2.48	2.75	2.53	2.45	2.66	2.49	2.43	2.59	2.44
^{79}TEGLRQE^{85}	2.18	2.05	2.50	2.10	2.23	2.08	2.27	2.15	2.02	2.39
^{63}QLGPVTQE^{70}	2.25	2.30	2.48	2.36	2.22	2.46	2.27	2.66	2.47	2.62

LC MS/MS analysis of GluC digests generated from apoA-I isolated from human atherosclerotic lesions yielded identification of ≥ 90% of the protein sequence. Peptide sequence and the distribution of Xcorr values produced by Proteome Discoverer are listed above.

Carbamylated lysine residue is shown in red

Xcorr (Proteome Discoverer) = cross-correlation value from the search.

(-)= Not detected.

Table S11. Kinetic and binding affinity data for lipid poor apoA-I binding to MPO determined by Surface Plasmon Resonance spectroscopy.

k_{on} (1/Ms)	k_{off} (1/s)	K_d (M)
5.02×10^{4}	3.77×10^{-3}	7.51×10^{-8}

To determine whether MPO specifically interacts with lipid-poor apoA-I we performed Surface Plasmon Resonance (SPR) spectroscopy measurements under physiological salt and pH conditions. MPO was immobilized on a CM5 sensor chip. Samples of apoA-I concentrations ranging from 0.5 nM to ~3000 nM were prepared in binding buffer (10 mM PBS, pH 7.4) and flowed over the sensor chip at a flow rate of 20 μL/min. The association rate constant (k_{on}), dissociation rate constant (k_{off}), and apparent dissociation constant (K_d) calculated from the kinetic/binding data are listed above. The results indicate that apoA-I binds tightly to MPO with K_d in the nanomolar range.

k_{on} is the kinetic rate constant for apoA-I/MPO binding.

k_{off} is the kinetic rate constant for apoA-I/MPO dissociation.

K_d is the dissociation constant of the complex MPO/apoA-I.

Table S12 .PRIDE accessible raw file names for in-vitro modified apoA-I and apoA-I isolated from human lesion samples used in the proteomics analysis. Files can be accessed using the data set identifier PXD027881.

Table S12A. ApoA-I samples used in the proteomics analysis shown in Fig. 3 and Fig. 4

	Sample Name	Sample Description	File Name
1	HDL_MPO	*in vitro* Modified rHDL by MPO system	HDL_MPO.raw
2	HDL_MPO2	*in vitro* Modified rHDL by MPO system	HDL_MPO2.raw
3	HDL_MPO3	*in vitro* Modified rHDL by MPO system	HDL_MPO3.raw
4	HDL_OCN	*in vitro* Modified rHDL by CNO- system	HDL_OCN.raw
5	HDL_OCN2	*in vitro* Modified rHDL by CNO- system	HDL_OCN2.raw
6	HDL_OCN3	*in vitro* Modified rHDL by CNO- system	HDL_OCN3.raw
7	LP_MPO	*in vitro* Modified LP apoA-I by MPO system	LP_MPO.raw
8	LP_MPO2	*in vitro* Modified LP apoA-I by MPO system	LP_MPO2.raw
9	LP_MPO3	*in vitro* Modified LP apoA-I by MPO system	LP_MPO3.raw
10	LP_OCN	*in vitro* Modified LP apoA-I by CNO- system	LP_OCN.raw
11	LP_OCN2	*in vitro* Modified LP apoA-I by CNO- system	LP_OCN2.raw
12	LP_OCN3	*in vitro* Modified LP apoA-I by CNO- system	LP_OCN3.raw

Table S12B. ApoA-I samples used in the in solution proteomic analysis shown in Fig. 5

	Sample Name	Sample Description	File Name
1	Lum_19may2101	apoA-I isolated from human subject 1	Lum_19may2101.raw
2	Lum_19may2102	apoA-I isolated from human subject 2	Lum_19may2102.raw
3	Lum_19may2103	apoA-I isolated from human subject 3	Lum_19may2103.raw
4	Lum_19may2104	apoA-I isolated from human subject 4	Lum_19may2104.raw
5	Lum_19may2105	apoA-I isolated from human subject 5	Lum_19may2105.raw
6	Lum_19may2106	apoA-I isolated from human subject 6	Lum_19may2106.raw
7	Lum_19may2107	apoA-I isolated from human subject 7	Lum_19may2107.raw
8	Lum_19may2108	apoA-I isolated from human subject 8	Lum_19may2108.raw
9	Lum_19may2109	apoA-I isolated from human subject 9	Lum_19may2109.raw
10	Lum_19may2110	apoA-I isolated from human subject 10	Lum_19may2110.raw

Table S12C. ApoA-I samples used in the in gel proteomic analysis shown in Fig. 6

	Sample Name	Sample Description	File Name
1	Lum_21nov1701	apoA-I Dimer isolated from human subject 1	Lum_21nov1701.raw
2	Lum_21nov1702	apoA-I Dimer isolated from human subject 2	Lum_21nov1702.raw
3	Lum_21nov1703	apoA-I Dimer isolated from human subject 3	Lum_21nov1703.raw
4	Lum_21nov1704	apoA-I Dimer isolated from human subject 4	Lum_21nov1704.raw
5	Lum_21nov1705	apoA-I Dimer isolated from human subject 5	Lum_21nov1705.raw
6	Lum_21nov1706	apoA-I Dimer isolated from human subject 6	Lum_21nov1706.raw
7	Lum_21nov1707	apoA-I Dimer isolated from human subject 7	Lum_21nov1707.raw
8	Lum_21nov1708	apoA-I Dimer isolated from human subject 8	Lum_21nov1708.raw
9	Lum_21nov1709	apoA-I Dimer isolated from human subject 9	Lum_21nov1709.raw
10	Lum_21nov1711-re	apoA-I Dimer isolated from human subject 10	Lum_21nov1711-re.raw

11	Lum_21nov1713	apoA-I Monomer isolated from human subject 1	Lum_21nov1713.raw
12	Lum_21nov1714	apoA-I Monomer isolated from human subject 2	Lum_21nov1714.raw
13	Lum_21nov1715	apoA-I Monomer isolated from human subject 3	Lum_21nov1715.raw
14	Lum_21nov1716	apoA-I Monomer isolated from human subject 4	Lum_21nov1716.raw
15	Lum_21nov1717	apoA-I Monomer isolated from human subject 5	Lum_21nov1717.raw
16	Lum_21nov1718	apoA-I Monomer isolated from human subject 6	Lum_21nov1718.raw
17	Lum_21nov1719	apoA-I Monomer isolated from human subject 7	Lum_21nov1719.raw
18	Lum_21nov1720	apoA-I Monomer isolated from human subject 8	Lum_21nov1720.raw
19	Lum_21nov1721	apoA-I Monomer isolated from human subject 9	Lum_21nov1721.raw
20	Lum_21nov1722	apoA-I Monomer isolated from human subject 10	Lum_21nov1722.raw

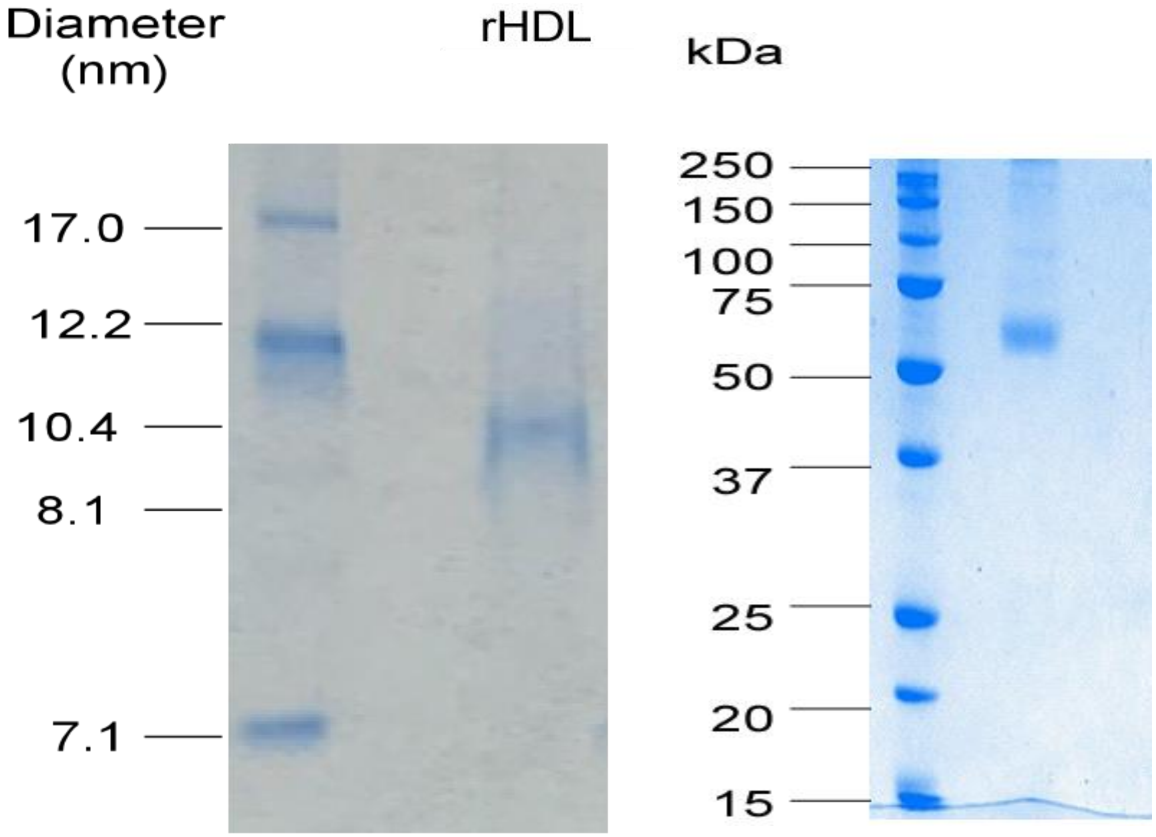

Figure S1. Characterization of reconstituted HDL particles. Reconstituted HDL (rHDL) particles were prepared using a modified cholate dialysis method at a molar ratio of 100:10:1, POPC: Cholesterol: apoA-I. Left: native-PAGE gel analysis of rHDL containing purified recombinant human apoA-I. Reconstituted HDL was purified by gel filtration and loaded (10 µg of protein) on a 4-20% gradient gel. The rHDL particle has a diameter of approximately 10 nm, consistent with preparations used in previous studies. Right: Purified 96-Å diameter POPC/Cholesterol rHDL-containing human apoA-I was cross-linked with BS3 cross-linker for 5 min at 37 °C and then quenched by the addition of Tris, pH 7.4 and samples fractionated on 12% SDS-PAGE gel. Left lane shows molecular mass standards and right lane shows bands for crosslinked rHDL.

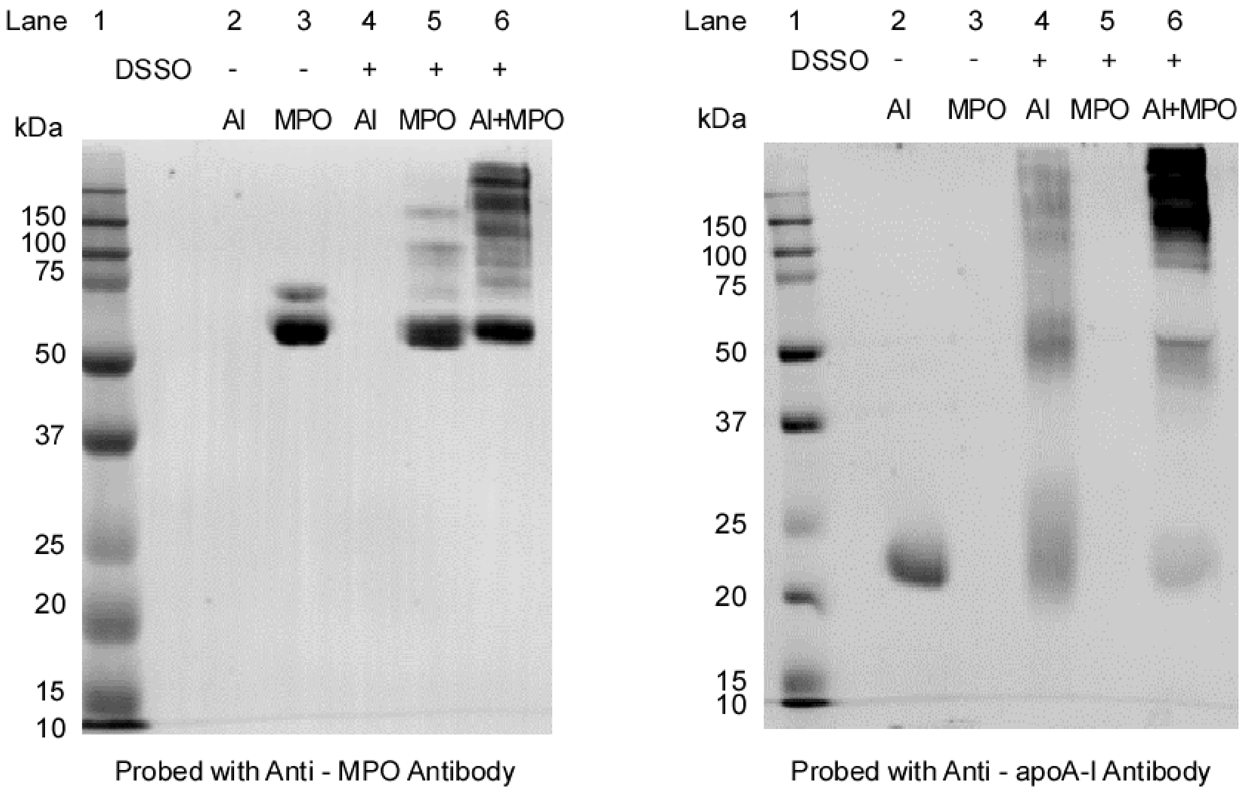

Figure S2. Western blot analysis of lipid poor apoA-I crosslinked with MPO. To further confirm the result of the Surface Plasmon Resonance (SPR) spectroscopy experiment, we crosslinked lipid poor apoA-I with MPO using the DSSO crosslinker as described in Experimental Procedures. The indicated samples were fractionated on a 12% reducing SDS-PAGE gel and proteins transferred to PVDF membranes for western blot analysis. A. Western blot analysis using anti-MPO antibody in the presence or absence of DSSO are shown in the gel. Lane 1: Marker, Lane 2: apoA-I only, Lane 3: MPO only, Lane 4: apoA-I+DSSO, Lane 5: MPO+DSSO, and Lane 6: MPO+apoA-I+DSSO. Samples were probed either with anti-MPO antibody or anti-apoA-I antibody (10G) to detect the presence of MPO or apoA-I in the sample, respectively.

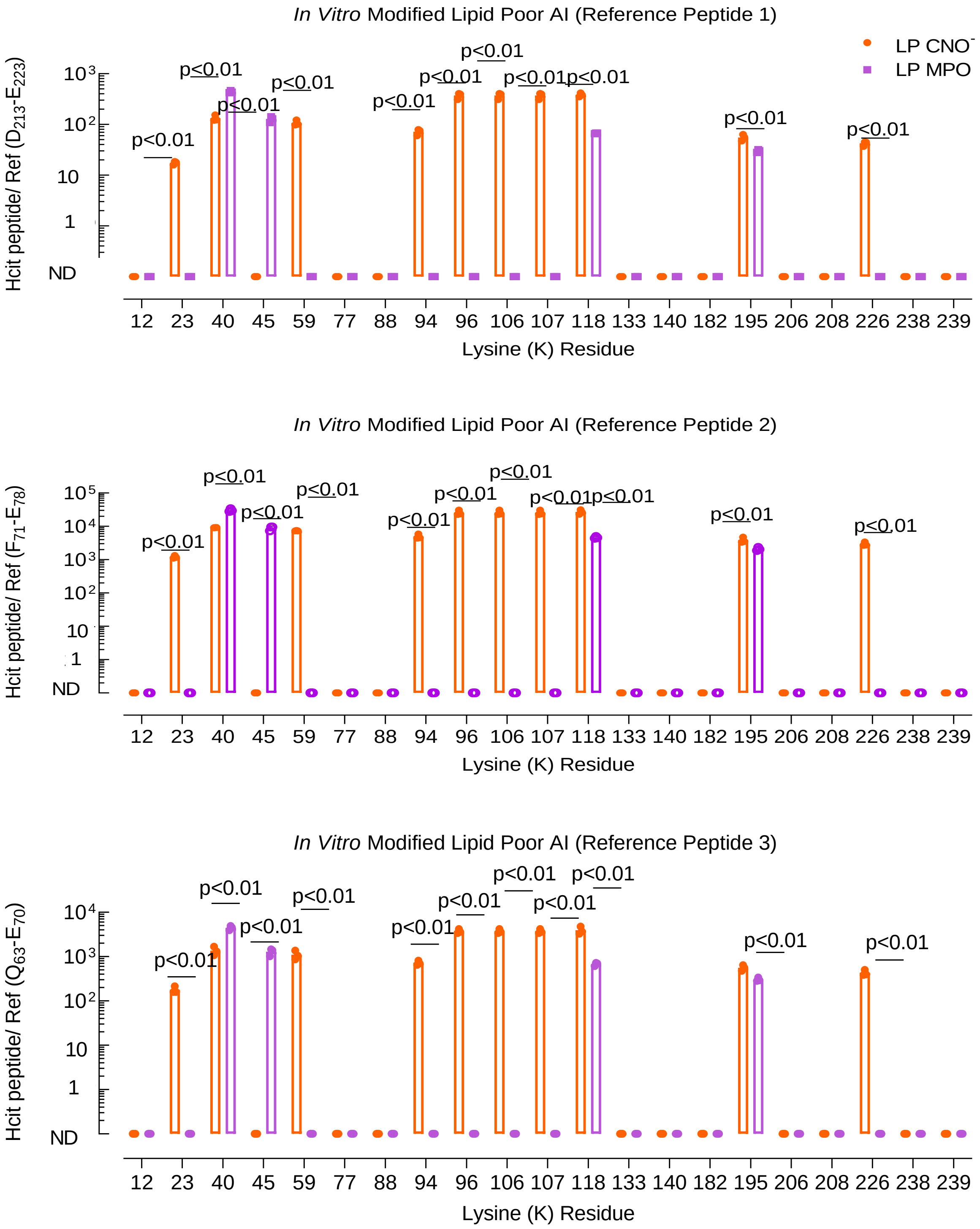

Figure S3. Quantification of carbamylated lysine residues in lipid poor apoA-I modified *in vitro* by the MPO or CNO^- system. The top, middle, and bottom panels show the peak areas of carbamylated lysine containing peptides divided by the peak area average of 3 distinct reference peptide candidates (multiplied by 10^4). Reference peptide candidates D_{213}-E_{223}, F_{71}-E_{78} and Q_{63}-E_{70} were used for quantitation in this analysis. Note that the overall patterns observed are highly aligned, regardless of which reference peptide was used.

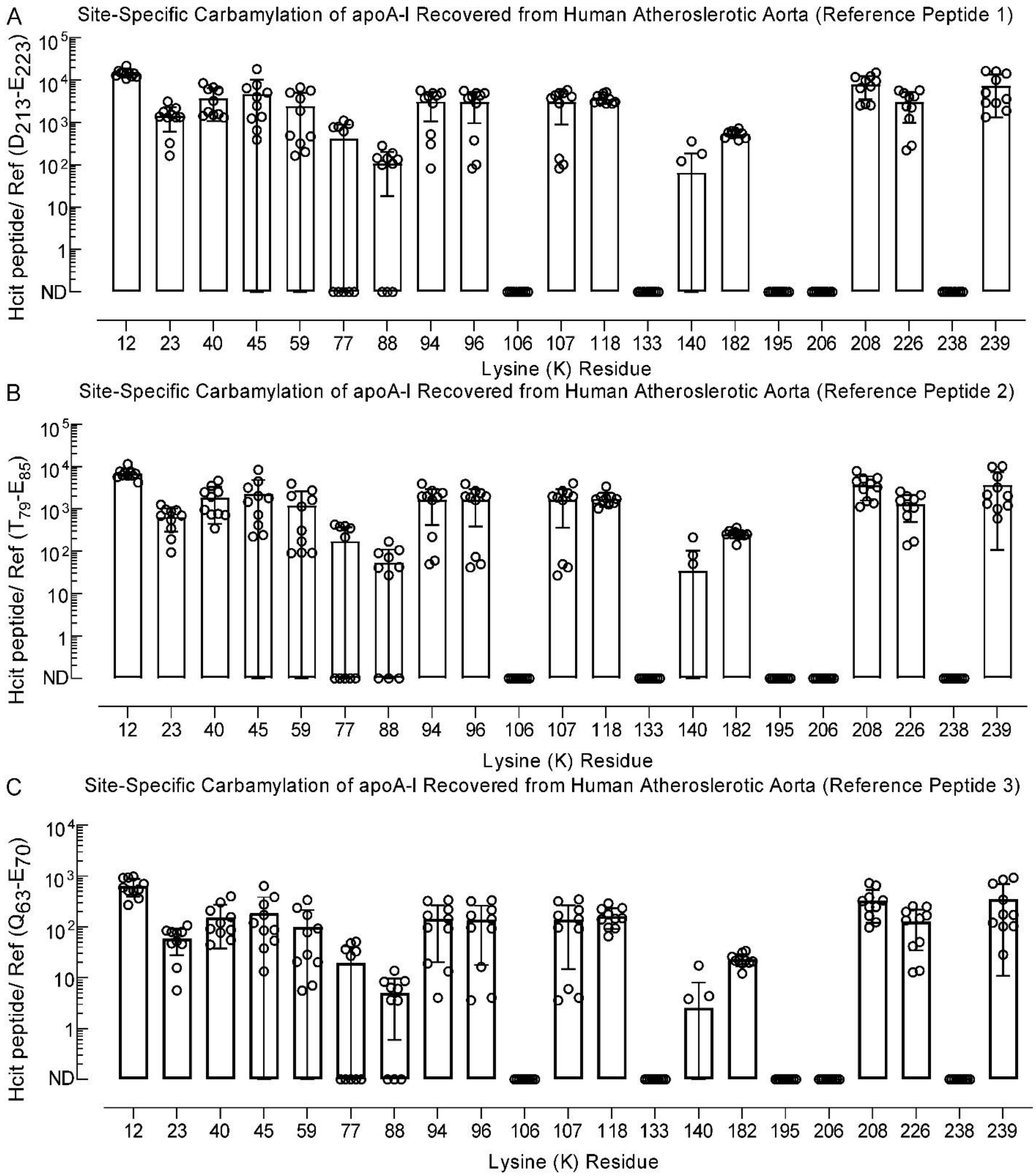

Figure S4. Quantification of site-specific carbamylation of apoA-I recovered from human atherosclerotic aorta. A-C. Each panel displays the peak area of peptides containing modified lysine residues divided by the peak area of a distinct reference peptide candidates (multiplied by 10^4). Reference peptide candidates D_{213}-E_{223}, T_{79}-E_{85} and Q_{63}-E_{70} were used for quantitation in this analysis. Note that the overall patterns observed are highly aligned, regardless of which reference peptide was used.

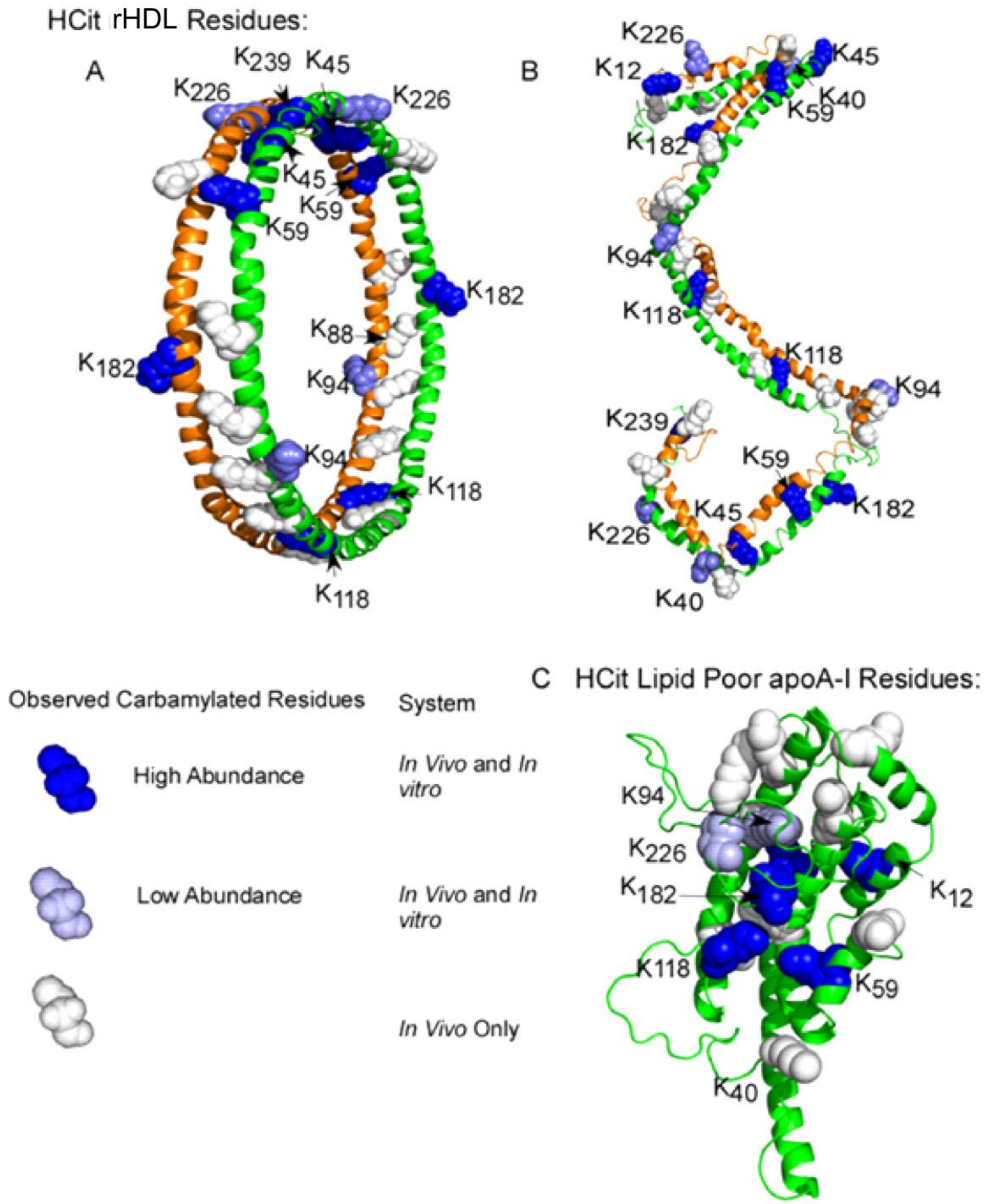

Figure S5. Lysine carbamylation sites identified by the proteomic analysis of rHDL and lipid poor apoA-I modified *in vitro* by CNO$^-$. A) Lysine residues carbamylated by the CNO$^-$ system in rHDL are mapped onto the Double Belt model of rHDL. B) Lysine residues carbamylated by the CNO$^-$ system in rHDL are mapped onto the Double Super Helix model of rHDL. C) Lysine residues carbamylated by the CNO$^-$ system in lipid poor apoA-I are mapped onto a model of lipid poor apoA-I. Dark blue spheres depict lysine residues that were carbamylated to the greatest extent, while the light blue spheres depict lysine residues that are carbamylated to a lesser extent in the *in vitro* model systems. White spheres are additional sites that were observed to be carbamylated in apoA-I recovered from human atherosclerotic aorta.

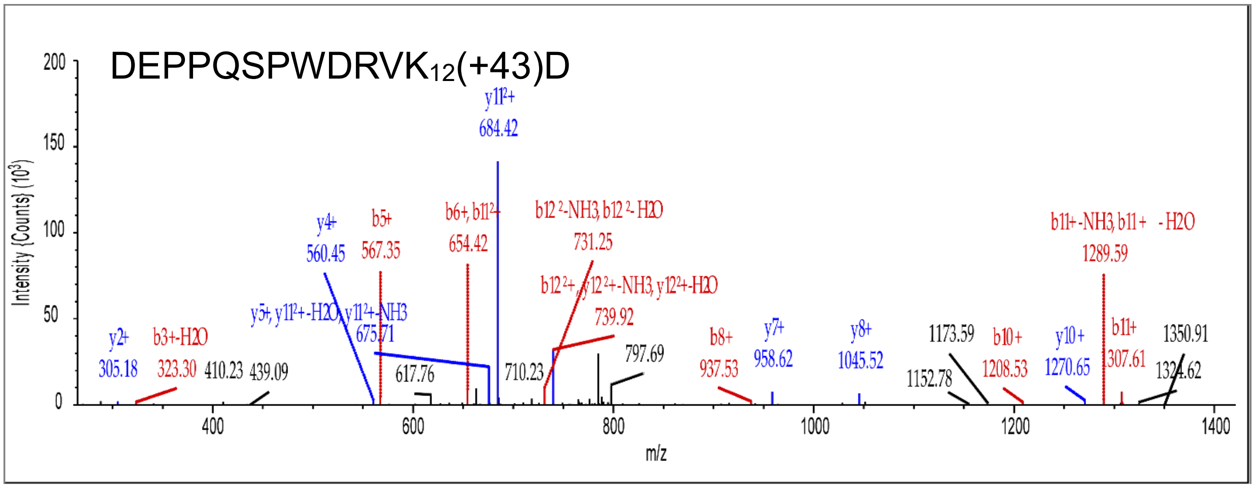

#1	b⁺	b²⁺	Seq.	y⁺	y²⁺	#2
1	116.03422	58.52075	D			13
2	245.07681	123.04204	E	1496.71283	748.86005	12
3	342.12958	171.56843	P	1367.67024	684.33876	11
4	439.18234	220.09481	P	1270.61748	635.81238	10
5	567.24092	284.12410	Q	1173.56471	587.28599	9
6	654.27295	327.64011	S	1045.50613	523.25671	8
7	751.32571	376.16649	P	958.47411	479.74069	7
8	937.40502	469.20615	W	861.42134	431.21431	6
9	1052.43197	526.71962	D	675.34203	338.17465	5
10	1208.53308	604.77018	R	560.31509	280.66118	4
11	1307.60149	654.30438	V	404.21398	202.61063	3
12	1478.70227	739.85477	K-Carbamyl	305.14556	153.07642	2
13			D	134.04478	67.52603	1

Sample:	Observed in:
Human plaque	yes
In-Vitro rHDL OCN	ND
In-Vitro rHDL MPO	ND
In-Vitro LP apoA-I OCN	ND
In-Vitro LP apoA-I MPO	ND

Figure S6: Annotated MS/MS spectrum and table of fragmentation ions for carbamylated apoA-I peptide DEPPQSPWDRVK$_{12}$(carbamyl)D. Human apoA-I was either recovered via immunoprecipitation from homogenized human atherosclerotic aorta (Human plaque), or recombinant human apoA-I (lipid poor (LP) vs. within rHDL) was exposed to the indicated *in vitro* carbamylation systems. ApoA-I was then subjected to proteolysis and proteomics analyses as described under Experimental Procedures. Top - CID spectrum for the indicated apoA-I peptide sequence harboring a carbamyllysine residue. Middle – Fragmentation table of peptide ions. Bottom – Table stating whether the carbamyllysine (K+43)-harboring apoA-I peptide was observed either in apoA-I recovered from human plaque or within the indicated *in vitro* carbamylated apoA-I samples. ND, not detected.

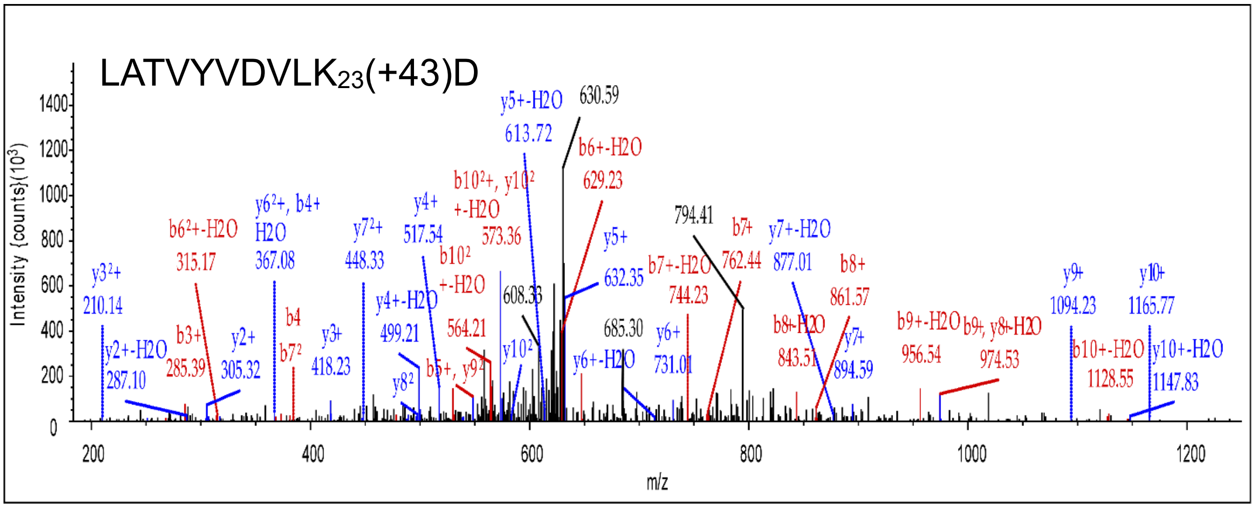

#1	b⁺	b²⁺	Seq.	y⁺	y²⁺	#2
1	114.09134	57.54931	L			11
2	185.12845	93.06787	A	1165.60993	583.30860	10
3	286.17613	143.59170	T	1094.57282	547.79005	9
4	385.24455	193.12591	V	993.52514	497.26621	8
5	548.30788	274.65758	Y	894.45672	447.73200	7
6	647.37629	324.19178	V	731.39340	366.20034	6
7	762.40323	381.70525	D	632.32498	316.66613	5
8	861.47165	431.23946	V	517.29804	259.15266	4
9	974.55571	487.78149	L	418.22963	209.61845	3
10	1145.65649	573.33188	K-Carbamyl	305.14556	153.07642	2
11			D	134.04478	67.52603	1

Sample:	Observed in:
Human plaque	yes
In-Vitro rHDL OCN	ND
In-Vitro rHDL MPO	yes
In-Vitro LP apoA-I OCN	yes
In-Vitro LP apoA-I MPO	ND

Figure S7: Annotated MS/MS spectrum and table of fragmentation ions for carbamylated apoA-I peptide LATVYVDVLK$_{23}$(carbamyl)D. Human apoA-I was either recovered via immunoprecipitation from homogenized human atherosclerotic aorta (Human plaque), or recombinant human apoA-I (lipid poor (LP) vs within rHDL) was exposed to the indicated *in vitro* carbamylation systems. ApoA-I was then subjected to proteolysis and proteomics analyses as described under Experimental Procedures. Top - CID spectrum for the indicated apoA-I peptide sequence harboring a carbamyllysine residue. Middle – Fragmentation table of peptide ions. Bottom – Table stating whether the carbamyllysine (K+43)-harboring apoA-I peptide was observed either in apoA-I recovered from human plaque or within the *indicated in vitro* carbamylated apoA-I samples. ND, not detected.

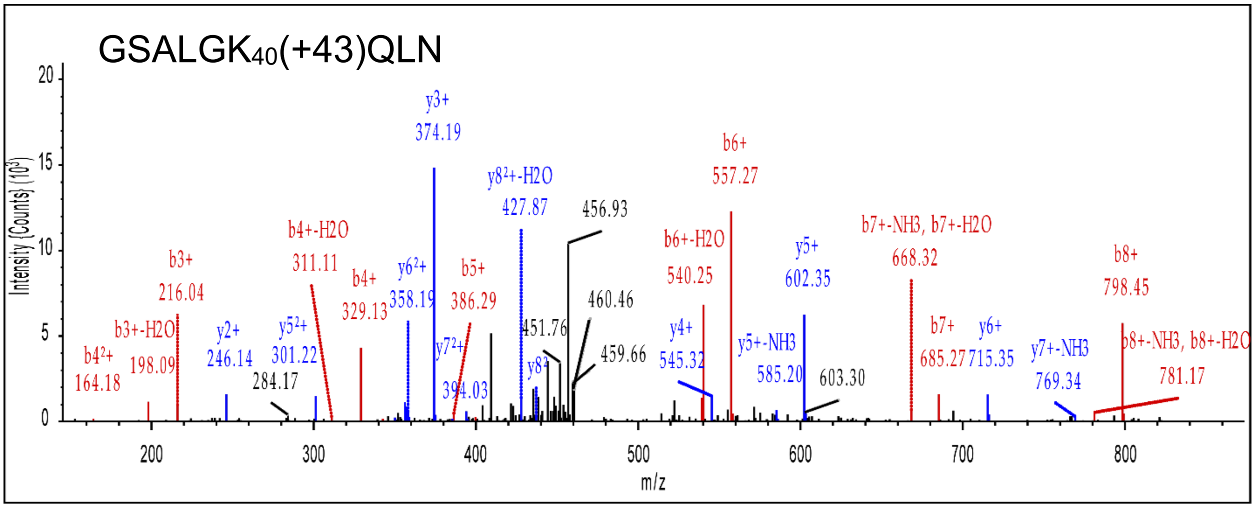

#1	b^+	b^{2+}	Seq.	y^+	y^{2+}	#2
1	58.02874	29.51801	G			9
2	145.06077	73.03402	S	873.47886	437.24307	8
3	216.09788	108.55258	A	786.44683	393.72705	7
4	329.18195	165.09461	L	715.40971	358.20850	6
5	386.20341	193.60534	G	602.32565	301.66646	5
6	557.30419	279.15573	K-Carbamyl	545.30419	273.15573	4
7	685.36276	343.18502	Q	374.20341	187.60534	3
8	798.44683	399.72705	L	246.14483	123.57605	2
9			N	133.06077	67.03402	1

Sample:	Observed in:
Human plaque	yes
In-Vitro rHDL OCN	yes
In-Vitro rHDL MPO	yes
In-Vitro LP apoA-I OCN	yes
In-Vitro LP apoA-I MPO	yes

Figure S8: Annotated MS/MS spectrum and table of fragmentation ions for carbamylated apoA-I peptide GSALGK$_{40}$(carbamyl)QLN. Human apoA-I was either recovered via immunoprecipitation from homogenized human atherosclerotic aorta (Human plaque), or recombinant human apoA-I (lipid poor (LP) vs within rHDL) was exposed to the indicated *in vitro* carbamylation systems. ApoA-I was then subjected to proteolysis and proteomics analyses as described under Experimental Procedures. Top - CID spectrum for the indicated apoA-I peptide sequence harboring a carbamyllysine residue. Middle – Fragmentation table of peptide ions. Bottom – Table stating whether the carbamyllysine (K+43)-harboring apoA-I peptide was observed either in apoA-I recovered from human plaque or within the indicated *in vitro* carbamylated apoA-I samples.

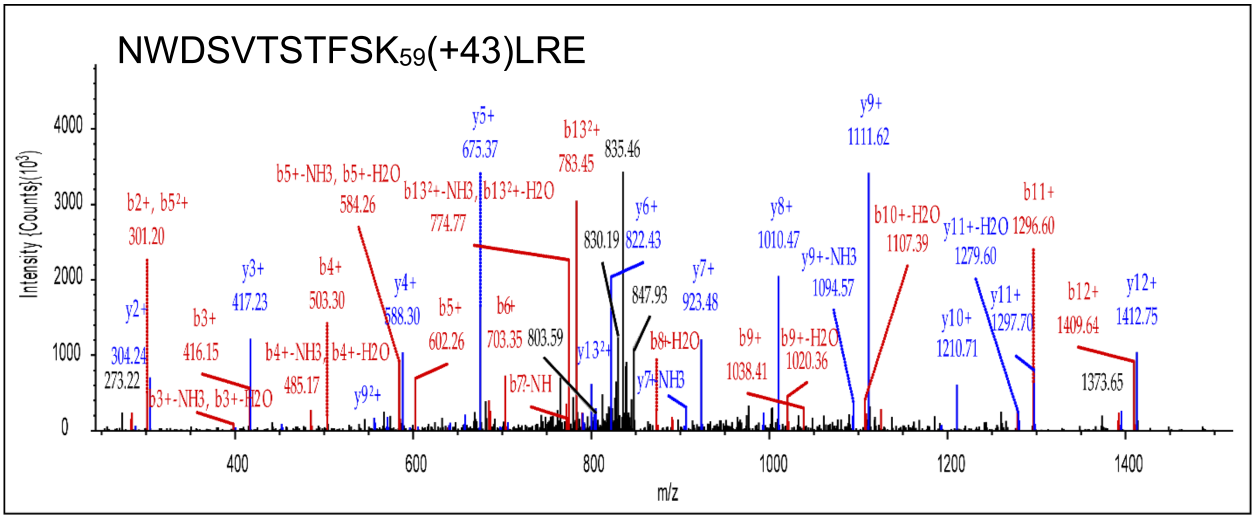

#1	b⁺	b²⁺	Seq.	y⁺	y²⁺	#2
1	115.05020	58.02874	N			14
2	301.12952	151.06840	W	1598.78091	799.89409	13
3	416.15646	208.58187	D	1412.70160	706.85444	12
4	503.18849	252.09788	S	1297.67466	649.34097	11
5	602.25690	301.63209	V	1210.64263	605.82495	10
6	703.30458	352.15593	T	1111.57421	556.29075	9
7	790.33661	395.67194	S	1010.52654	505.76691	8
8	891.38429	446.19578	T	923.49451	462.25089	7
9	1038.45270	519.72999	F	822.44683	411.72705	6
10	1125.48473	563.24600	S	675.37841	338.19285	5
11	1296.58551	648.79639	K-Carbamyl	588.34639	294.67683	4
12	1409.66957	705.33842	L	417.24561	209.12644	3
13	1565.77068	783.38898	R	304.16155	152.58441	2
14			E	148.06043	74.53386	1

Sample:	Observed in:
Human plaque	yes
In-Vitro rHDL OCN	yes
In-Vitro rHDL MPO	ND
In-Vitro LP apoA-I OCN	yes
In-Vitro LP apoA-I MPO	ND

Figure S9: Annotated MS/MS spectrum and table of fragmentation ions for carbamylated apoA-I peptide NWDSVTSTFSK$_{59}$(carbamyl)LRE. Human apoA-I was either recovered via immunoprecipitation from homogenized human atherosclerotic aorta (Human plaque), or recombinant human apoA-I (lipid poor (LP) vs within rHDL) was exposed to the indicated *in vitro* carbamylation systems. ApoA-I was then subjected to proteolysis and proteomics analyses as described under Experimental Procedures. Top - CID spectrum for the indicated apoA-I peptide sequence harboring a carbamyllysine residue. Middle – Fragmentation table of peptide ions. Bottom – Table stating whether the carbamyllysine (K+43)-harboring apoA-I peptide was observed either in apoA-I recovered from human plaque or within the indicated *in vitro* carbamylated apoA-I samples.

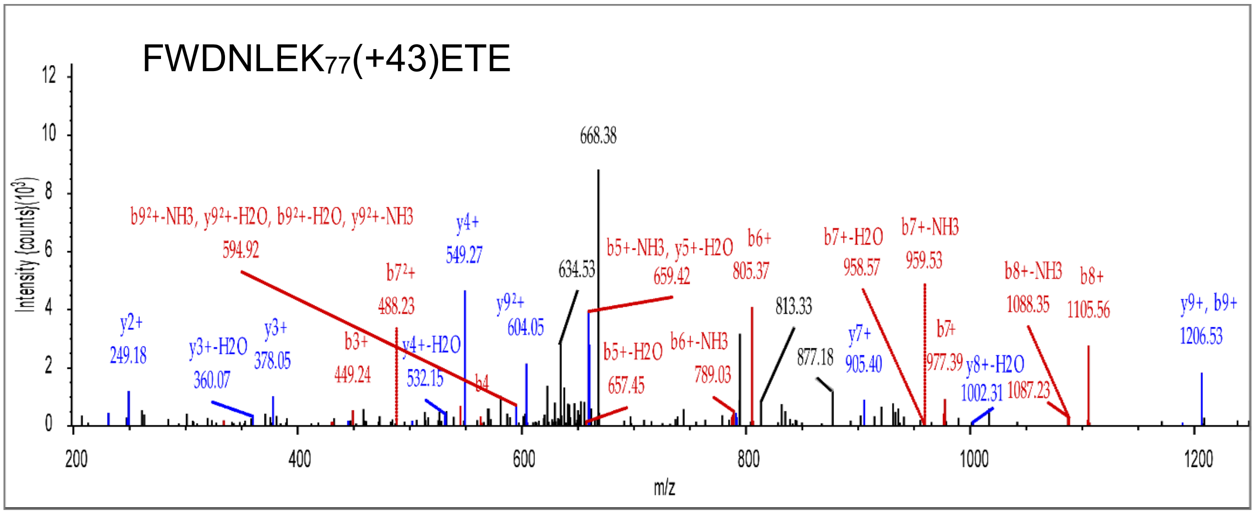

#1	b⁺	b²⁺	Seq.	y⁺	y²⁺	#2
1	148.07569	74.54148	F			10
2	334.15500	167.58114	W	1206.52732	603.76730	9
3	449.18195	225.09461	D	1020.44801	510.72764	8
4	563.22487	282.11608	N	905.42107	453.21417	7
5	676.30894	338.65811	L	791.37814	396.19271	6
6	805.35153	403.17940	E	678.29408	339.65068	5
7	976.45231	488.72979	K-Carbamyl	549.25148	275.12938	4
8	1105.49490	553.25109	E	378.15071	189.57899	3
9	1206.54258	603.77493	T	249.10811	125.05769	2
10			E	148.06043	74.53386	1

Sample:	Observed in:
Human plaque	yes
In-Vitro rHDL OCN	ND
In-Vitro rHDL MPO	ND
In-Vitro LP apoA-I OCN	ND
In-Vitro LP apoA-I MPO	ND

Figure S10: Annotated MS/MS spectrum and table of fragmentation ions for carbamylated apoA-I peptide FWDNLEK$_{77}$(carbamyl)ETE. Human apoA-I was either recovered via immunoprecipitation from homogenized human atherosclerotic aorta (Human plaque), or recombinant human apoA-I (lipid poor (LP) vs within rHDL) was exposed to the indicated *in vitro* carbamylation systems. ApoA-I was then subjected to proteolysis and proteomics analyses as described under Experimental Procedures. Top - CID spectrum for the indicated apoA-I peptide sequence harboring a carbamyllysine residue. Middle – Fragmentation table of peptide ions. Bottom – Table stating whether the carbamyllysine (K+43)-harboring apoA-I peptide was observed either in apoA-I recovered from human plaque or within the indicated *in vitro* carbamylated apoA-I samples.

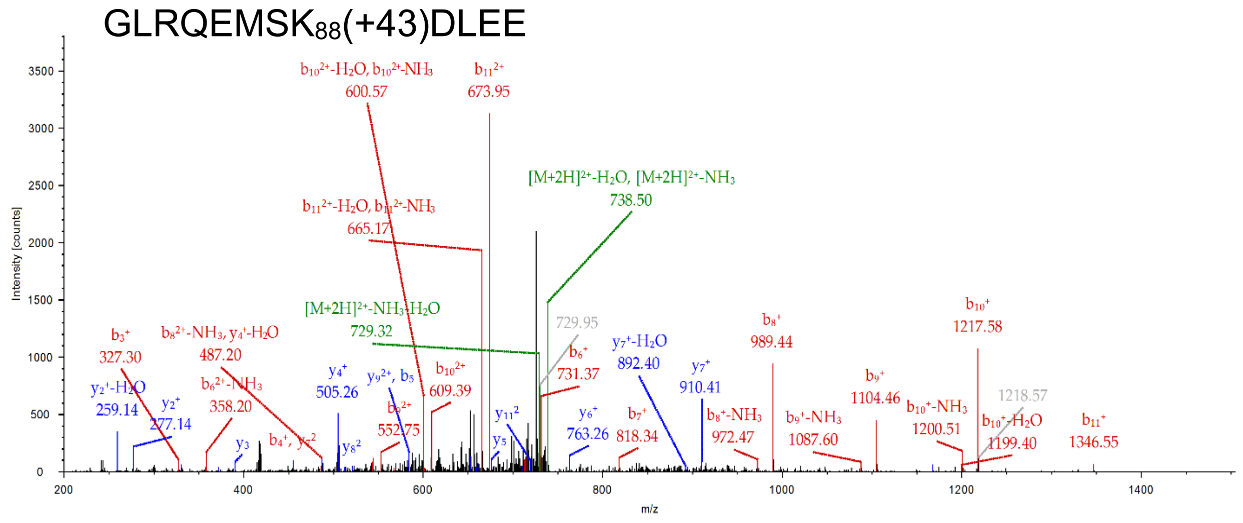

#1	b^+	b^{2+}	Seq.	y^+	y^{2+}	#2
1	58.02874	29.51801	G			12
2	171.11280	86.06004	L	1436.66858	718.83793	11
3	327.21392	164.11060	R	1323.58452	662.29590	10
4	455.27249	228.13988	Q	1167.48341	584.24534	9
5	584.31509	292.66118	E	1039.42483	520.21605	8
6	731.35049	366.17888	M-Oxidation	910.38224	455.69476	7
7	818.38251	409.69490	S	763.34684	382.17706	6
8	989.48329	495.24528	K-Carbamyl	676.31481	338.66104	5
9	1104.51023	552.75876	D	505.21403	253.11066	4
10	1217.59430	609.30079	L	390.18709	195.59718	3
11	1346.63689	673.82208	E	277.10303	139.05515	2
12			E	148.06043	74.53386	1

Sample:	Observed in:
Human plaque	yes
In-Vitro rHDL OCN	ND
In-Vitro rHDL MPO	ND
In-Vitro LP apoA-I OCN	ND
In-Vitro LP apoA-I MPO	ND

Figure S11: Annotated MS/MS spectrum and table of fragmentation ions for carbamylated apoA-I peptide GLRQEMSK$_{88}$(carbamyl)DLEE. Human apoA-I was either recovered via immunoprecipitation from homogenized human atherosclerotic aorta (Human plaque), or recombinant human apoA-I (lipid poor (LP) vs within rHDL) was exposed to the indicated *in vitro* carbamylation systems. ApoA-I was then subjected to proteolysis and proteomics analyses as described under Experimental Procedures. Top - CID spectrum for the indicated apoA-I peptide sequence harboring a carbamyllysine residue. Middle – Fragmentation table of peptide ions. Bottom – Table stating whether the carbamyllysine (K+43)-harboring apoA-I peptide was observed either in apoA-I recovered from human plaque or within the indicated *in vitro* carbamylated apoA-I samples.

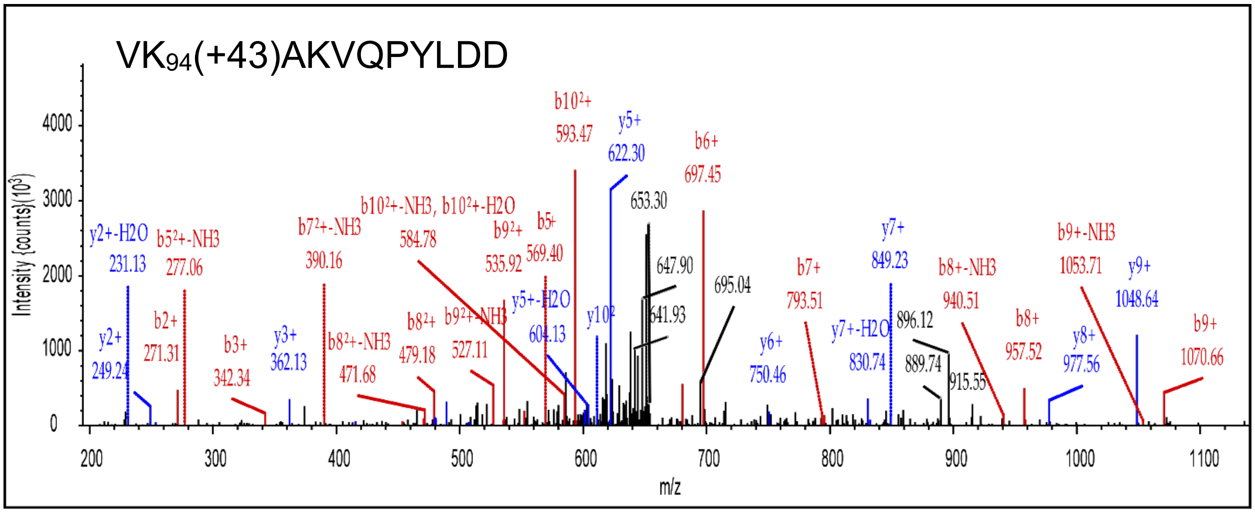

#1	b⁺	b²⁺	Seq.	y⁺	y²⁺	#2
1	100.07569	50.54148	V			11
2	271.17647	136.09187	K-Carbamyl	1219.63173	610.31950	10
3	342.21358	171.61043	A	1048.53095	524.76911	9
4	470.30854	235.65791	K	977.49384	489.25056	8
5	569.37696	285.19212	V	849.39887	425.20308	7
6	697.43554	349.22141	Q	750.33046	375.66887	6
7	794.48830	397.74779	P	622.27188	311.63958	5
8	957.55163	479.27945	Y	525.21912	263.11320	4
9	1070.63569	535.82148	L	362.15579	181.58153	3
10	1185.66263	593.33496	D	249.07173	125.03950	2
11			D	134.04478	67.52603	1

Sample:	Observed in:
Human plaque	yes
In-Vitro rHDL OCN	yes
In-Vitro rHDL MPO	yes
In-Vitro LP apoA-I OCN	yes
In-Vitro LP apoA-I MPO	ND

Figure S12: Annotated MS/MS spectrum and table of fragmentation ions for carbamylated apoA-I peptide VK$_{94}$(carbamyl)AKVQPYLDD. Human apoA-I was either recovered via immunoprecipitation from homogenized human atherosclerotic aorta (Human plaque), or recombinant human apoA-I (lipid poor (LP) vs within rHDL) was exposed to the indicated *in vitro* carbamylation systems. ApoA-I was then subjected to proteolysis and proteomics analyses as described under Experimental Procedures. Top - CID spectrum for the indicated apoA-I peptide sequence harboring a carbamyllysine residue. Middle – Fragmentation table of peptide ions. Bottom – Table stating whether the carbamyllysine (K+43)-harboring apoA-I peptide was observed either in apoA-I recovered from human plaque or within the indicated *in vitro* carbamylated apoA-I samples.

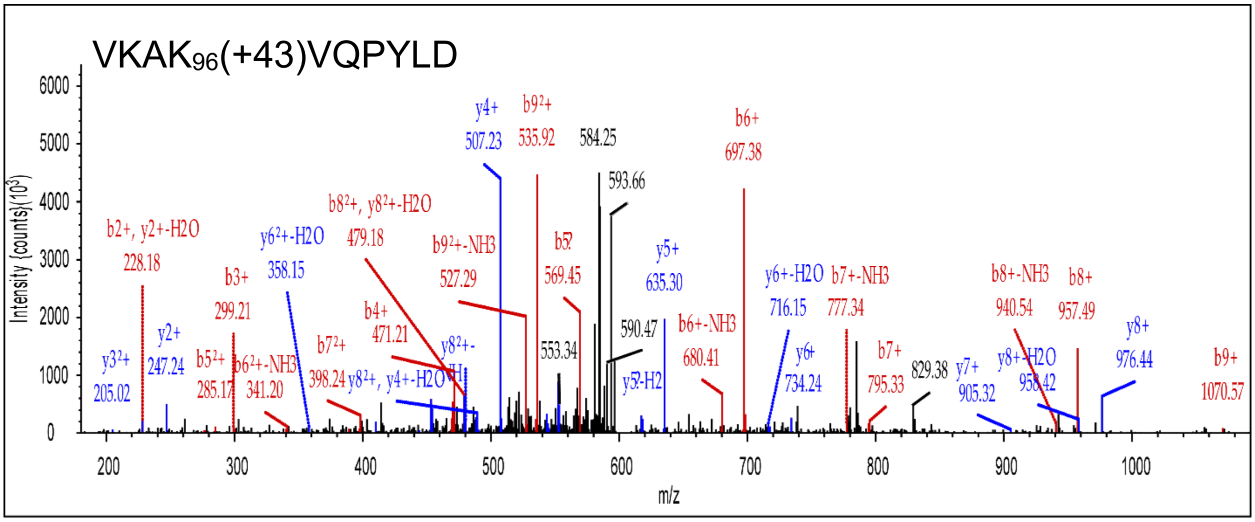

#1	b⁺	b²⁺	Seq.	y⁺	y²⁺	#2
1	100.07569	50.54148	V			10
2	228.17065	114.58897	K	1104.60479	552.80603	9
3	299.20777	150.10752	A	976.50982	488.75855	8
4	470.30854	235.65791	K-Carbamyl	905.47271	453.23999	7
5	569.37696	285.19212	V	734.37193	367.68960	6
6	697.43554	349.22141	Q	635.30352	318.15540	5
7	794.48830	397.74779	P	507.24494	254.12611	4
8	957.55163	479.27945	Y	410.19218	205.59973	3
9	1070.63569	535.82148	L	247.12885	124.06806	2
10			D	134.04478	67.52603	1

Sample:	Observed in:
Human plaque	yes
In-Vitro rHDL OCN	ND
In-Vitro rHDL MPO	ND
In-Vitro LP apoA-I OCN	yes
In-Vitro LP apoA-I MPO	ND

Figure S13: Annotated MS/MS spectrum and table of fragmentation ions for carbamylated apoA-I peptide VKAK$_{96}$(carbamyl)VQPYLD. Human apoA-I was either recovered via immunoprecipitation from homogenized human atherosclerotic aorta (Human plaque), or recombinant human apoA-I (lipid poor (LP) vs within rHDL) was exposed to the indicated *in vitro* carbamylation systems. ApoA-I was then subjected to proteolysis and proteomics analyses as described under Experimental Procedures. Top - CID spectrum for the indicated apoA-I peptide sequence harboring a carbamyllysine residue. Middle – Fragmentation table of peptide ions. Bottom – Table stating whether the carbamyllysine (K+43)-harboring apoA-I peptide was observed either in apoA-I recovered from human plaque or within the indicated *in vitro* carbamylated apoA-I samples.

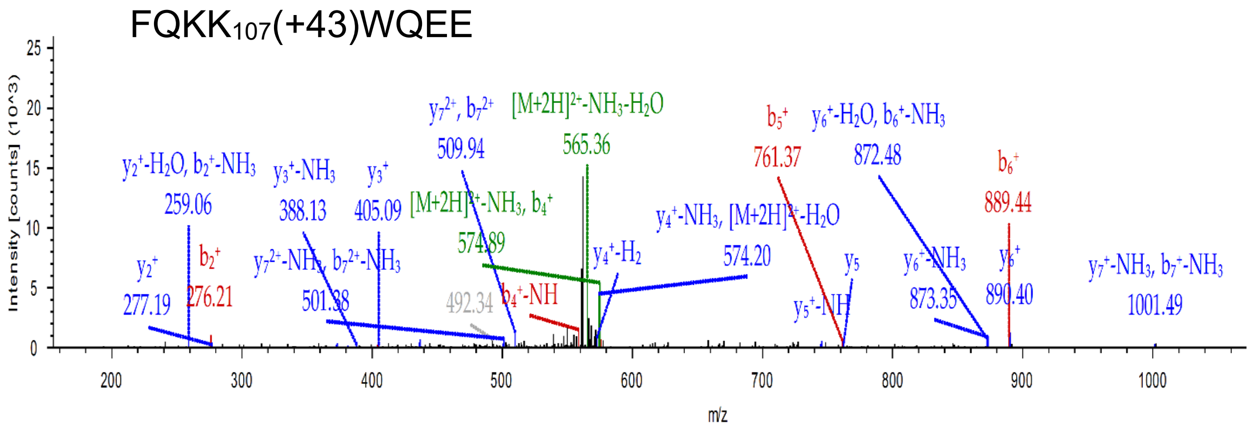

#1	b⁺	b²⁺	Seq.	y⁺	y²⁺	#2
1	148.07569	74.54148	F			8
2	276.13427	138.57077	Q	1018.49524	509.75126	7
3	404.22923	202.61825	K	890.43666	445.72197	6
4	575.33001	288.16864	K-Carbamyl	762.34169	381.67449	5
5	761.40932	381.20830	W	591.24092	296.12410	4
6	889.46790	445.23759	Q	405.16160	203.08444	3
7	1018.51049	509.75888	E	277.10303	139.05515	2
8			E	148.06043	74.53386	1

Sample:	Observed in:
Human plaque	yes
In-Vitro rHDL OCN	ND
In-Vitro rHDL MPO	ND
In-Vitro LP apoA-I OCN	yes
In-Vitro LP apoA-I MPO	ND

Figure S14: Annotated MS/MS spectrum and table of fragmentation ions for carbamylated apoA-I peptide FQKK$_{107}$(carbamyl)WQEE. Human apoA-I was either recovered via immunoprecipitation from homogenized human atherosclerotic aorta (Human plaque), or recombinant human apoA-I (lipid poor (LP) vs within rHDL) was exposed to the indicated *in vitro* carbamylation systems. ApoA-I was then subjected to proteolysis and proteomics analyses as described under Experimental Procedures. Top - CID spectrum for the indicated apoA-I peptide sequence harboring a carbamyllysine residue. Middle – Fragmentation table of peptide ions. Bottom – Table stating whether the carbamyllysine (K+43)-harboring apoA-I peptide was observed either in apoA-I recovered from human plaque or within the indicated *in vitro* carbamylated apoA-I samples.

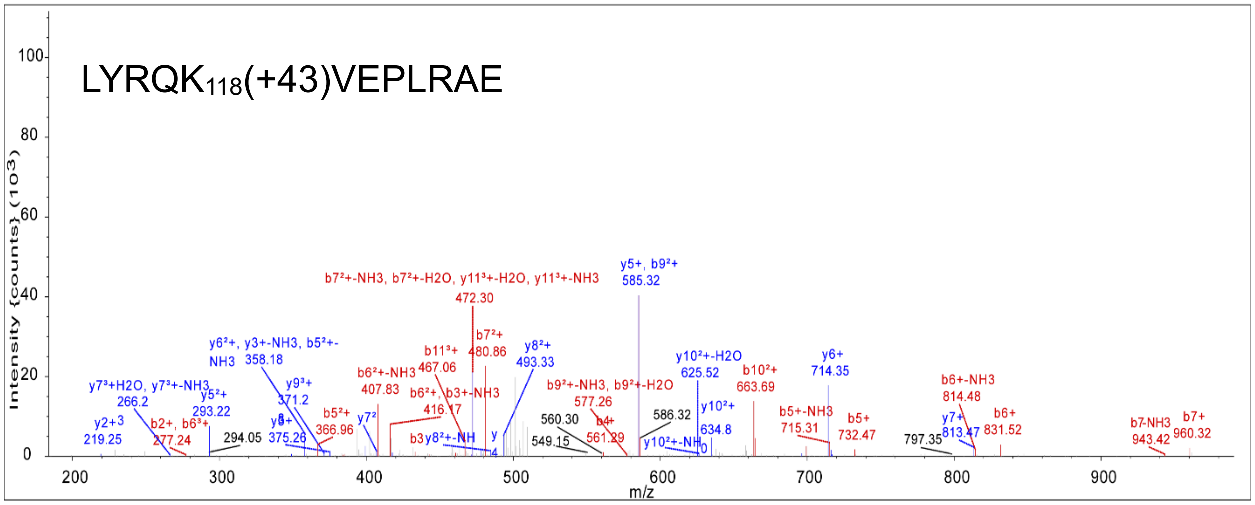

#1	b⁺	b²⁺	Seq.	y⁺	y²⁺	#2
1	114.09134	57.54931	L			12
2	277.15467	139.08097	Y	1431.77029	716.38878	11
3	433.25578	217.13153	R	1268.70696	634.85712	10
4	561.31436	281.16082	Q	1112.60585	556.80656	9
5	732.41513	366.71121	K-Carbamyl	984.54727	492.77727	8
6	831.48355	416.24541	V	813.44649	407.22689	7
7	960.52614	480.76671	E	714.37808	357.69268	6
8	1057.57891	529.29309	P	585.33549	293.17138	5
9	1170.66297	585.83512	L	488.28272	244.64500	4
10	1326.76408	663.88568	R	375.19866	188.10297	3
11	1397.80119	699.40424	A	219.09755	110.05241	2
12			E	148.06043	74.53386	1

Sample:	Observed in:
Human plaque	yes
In-Vitro rHDL OCN	ND
In-Vitro rHDL MPO	ND
In-Vitro LP apoA-I OCN	yes
In-Vitro LP apoA-I MPO	ND

Figure S15: Annotated MS/MS spectrum and table of fragmentation ions for carbamylated apoA-I peptide LYRQK$_{118}$(carbamyl)VEPLRAE. Human apoA-I was either recovered via immunoprecipitation from homogenized human atherosclerotic aorta (Human plaque), or recombinant human apoA-I (lipid poor (LP) vs within rHDL) was exposed to the indicated *in vitro* carbamylation systems. ApoA-I was then subjected to proteolysis and proteomics analyses as described under Experimental Procedures. Top - CID spectrum for the indicated apoA-I peptide sequence harboring a carbamyllysine residue. Middle – Fragmentation table of peptide ions. Bottom – Table stating whether the carbamyllysine (K+43)-harboring apoA-I peptide was observed either in apoA-I recovered from human plaque or within the indicated *in vitro* carbamylated apoA-I samples.

LQEK$_{140}$(+43)LSPLGEE

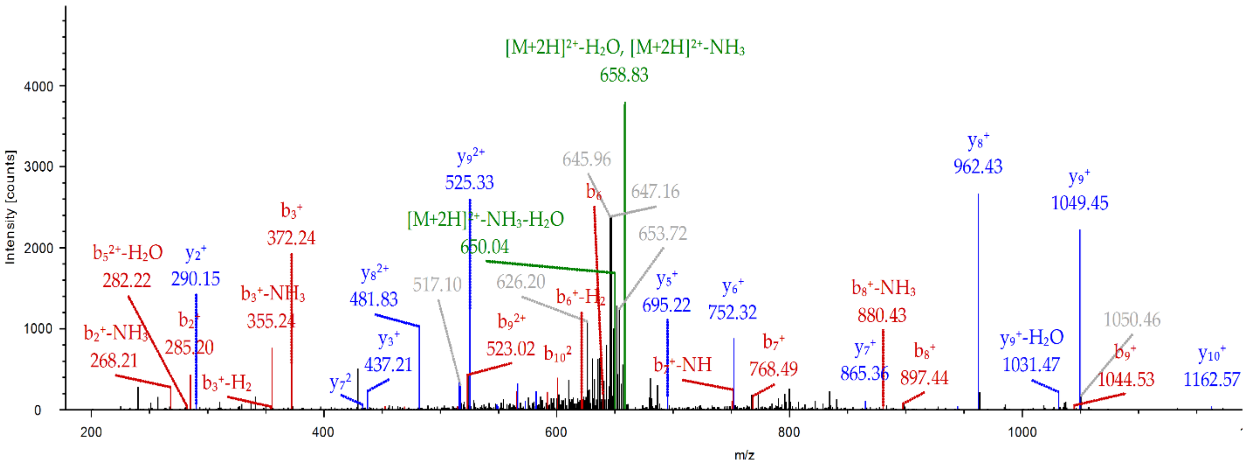

#1	b⁺	b²⁺	Seq.	y⁺	y²⁺	#2
1	114.09134	57.54931	L			11
2	242.14992	121.57860	Q	1172.57936	586.79332	10
3	371.19251	186.09989	E	1044.52078	522.76403	9
4	542.29329	271.65028	K-Carbamyl	915.47819	458.24273	8
5	655.37735	328.19231	L	744.37741	372.69234	7
6	742.40938	371.70833	S	631.29335	316.15031	6
7	839.46214	420.23471	P	544.26132	272.63430	5
8	952.54621	476.77674	L	447.20855	224.10792	4
9	1009.56767	505.28747	G	334.12449	167.56588	3
10	1138.61026	569.80877	E	277.10303	139.05515	2
11			E	148.06043	74.53386	1

Sample:	Observed in:
Human plaque	yes
In-Vitro rHDL OCN	ND
In-Vitro rHDL MPO	ND
In-Vitro LP apoA-I OCN	ND
In-Vitro LP apoA-I MPO	ND

Figure S16: Annotated MS/MS spectrum and table of fragmentation ions for carbamylated apoA-I peptide LQEK$_{140}$(carbamyl)LSPLGEE. Human apoA-I was either recovered via immunoprecipitation from homogenized human atherosclerotic aorta (Human plaque), or recombinant human apoA-I (lipid poor (LP) vs within rHDL) was exposed to the indicated *in vitro* carbamylation systems. ApoA-I was then subjected to proteolysis and proteomics analyses as described under Experimental Procedures. Top - CID spectrum for the indicated apoA-I peptide sequence harboring a carbamyllysine residue. Middle – Fragmentation table of peptide ions. Bottom – Table stating whether the carbamyllysine (K+43)-harboring apoA-I peptide was observed either in apoA-I recovered from human plaque or within the indicated *in vitro* carbamylated apoA-I samples.

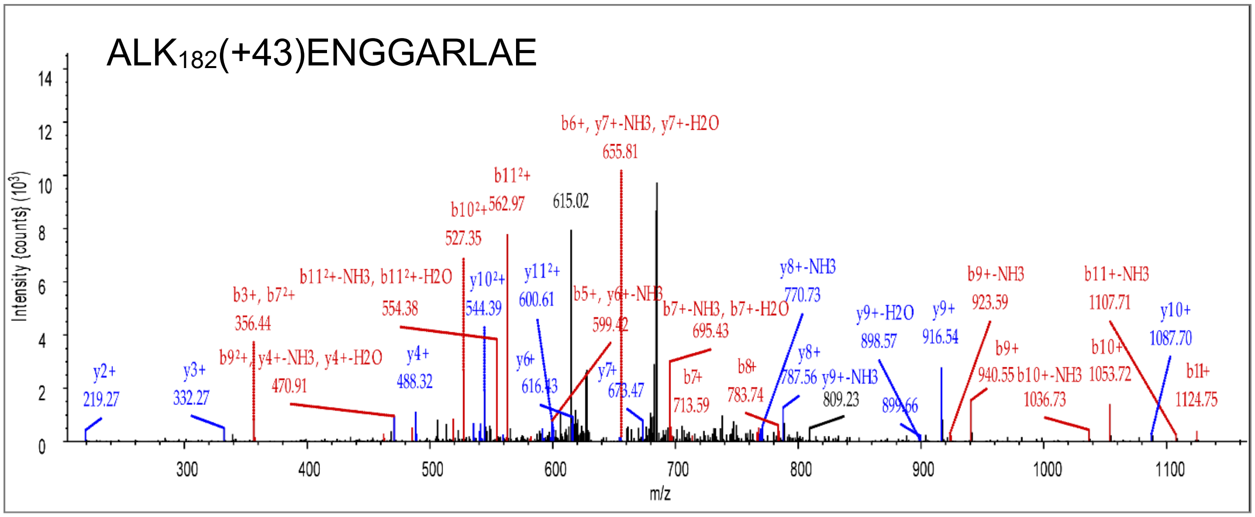

#1	b⁺	b²⁺	Seq.	y⁺	y²⁺	#2
1	72.04439	36.52583	A			12
2	185.12845	93.06787	L	1200.63313	600.82020	11
3	356.22923	178.61825	K-Carbamyl	1087.54906	544.27817	10
4	485.27182	243.13955	E	916.44828	458.72778	9
5	599.31475	300.16101	N	787.40569	394.20648	8
6	656.33622	328.67175	G	673.36276	337.18502	7
7	713.35768	357.18248	G	616.34130	308.67429	6
8	784.39479	392.70103	A	559.31984	280.16356	5
9	940.49590	470.75159	R	488.28272	244.64500	4
10	1053.57997	527.29362	L	332.18161	166.59444	3
11	1124.61708	562.81218	A	219.09755	110.05241	2
12			E	148.06043	74.53386	1

Sample:	Observed in:
Human plaque	yes
In-Vitro rHDL OCN	yes
In-Vitro rHDL MPO	ND
In-Vitro LP apoA-I OCN	ND
In-Vitro LP apoA-I MPO	ND

Figure S17: Annotated MS/MS spectrum and table of fragmentation ions for carbamylated apoA-I peptide ALK$_{182}$(carbamyl)ENGGARLAE. Human apoA-I was either recovered via immunoprecipitation from homogenized human atherosclerotic aorta (Human plaque), or recombinant human apoA-I (lipid poor (LP) vs within rHDL) was exposed to the indicated *in vitro* carbamylation systems. ApoA-I was then subjected to proteolysis and proteomics analyses as described under Experimental Procedures. Top - CID spectrum for the indicated apoA-I peptide sequence harboring a carbamyllysine residue. Middle – Fragmentation table of peptide ions. Bottom – Table stating whether the carbamyllysine (K+43)-harboring apoA-I peptide was observed either in apoA-I recovered from human plaque or within the indicated *in vitro* carbamylated apoA-I samples.

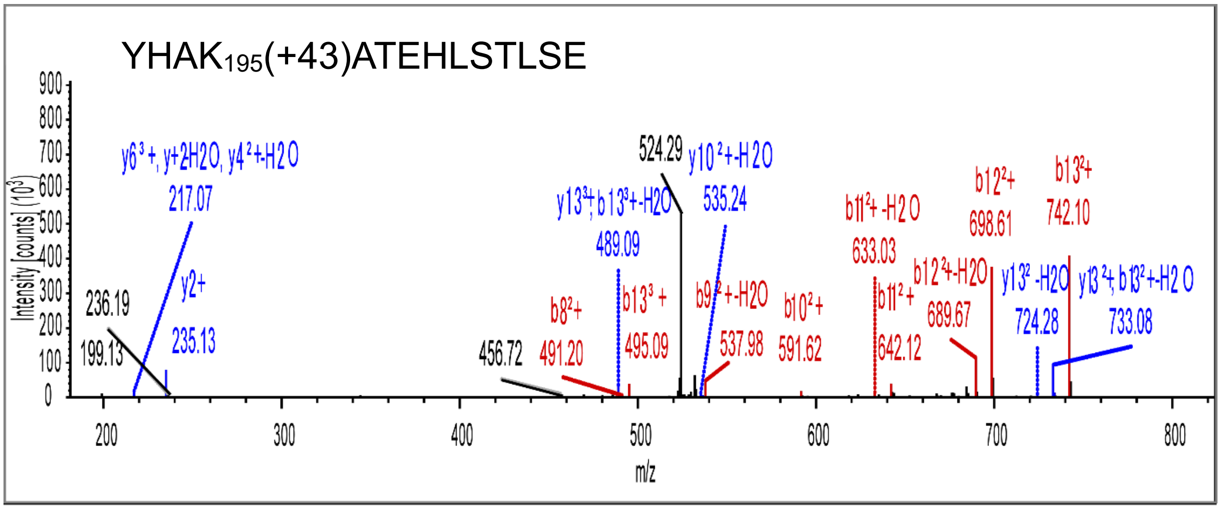

#1	b⁺	b²⁺	b³⁺	Seq.	y⁺	y²⁺	y³⁺	#2
1	164.07061	82.53894	55.36172	Y				14
2	301.12952	151.06840	101.04802	H	1466.72340	733.86534	489.57932	13
3	372.16663	186.58695	124.72706	A	1329.66448	665.33588	443.89301	12
4	543.26741	272.13734	181.76065	K-Carbamyl	1258.62737	629.81732	420.21397	11
5	614.30452	307.65590	205.43969	A	1087.52659	544.26694	363.18038	10
6	715.35220	358.17974	239.12225	T	1016.48948	508.74838	339.50134	9
7	844.39479	422.70103	282.13645	E	915.44180	458.22454	305.81879	8
8	981.45370	491.23049	327.82275	H	786.39921	393.70324	262.80459	7
9	1094.53777	547.77252	365.51744	L	649.34030	325.17379	217.11828	6
10	1181.56980	591.28854	394.52812	S	536.25623	268.63175	179.42360	5
11	1282.61748	641.81238	428.21068	T	449.22420	225.11574	150.41292	4
12	1395.70154	698.35441	465.90536	L	348.17653	174.59190	116.73036	3
13	1482.73357	741.87042	494.91604	S	235.09246	118.04987	79.03567	2
14				E	148.06043	74.53386	50.02500	1

Sample:	Observed in:
Human plaque	ND
In-Vitro rHDL OCN	ND
In-Vitro rHDL MPO	yes
In-Vitro LP apoA-I OCN	yes
In-Vitro LP apoA-I MPO	yes

Figure S18: Annotated MS/MS spectrum and table of fragmentation ions for carbamylated apoA-I peptide YHAK$_{195}$(carbamyl)ATEHLSTLSE. Human apoA-I was either recovered via immunoprecipitation from homogenized human atherosclerotic aorta (Human plaque), or recombinant human apoA-I (lipid poor (LP) vs within rHDL) was exposed to the indicated *in vitro* carbamylation systems. ApoA-I was then subjected to proteolysis and proteomics analyses as described under Experimental Procedures. Top - CID spectrum for the indicated apoA-I peptide sequence harboring a carbamyllysine residue. Middle – Fragmentation table of peptide ions. Bottom – Table stating whether the carbamyllysine (K+43)-harboring apoA-I peptide was observed either in apoA-I recovered from human plaque or within the indicated *in vitro* carbamylated apoA-I samples.

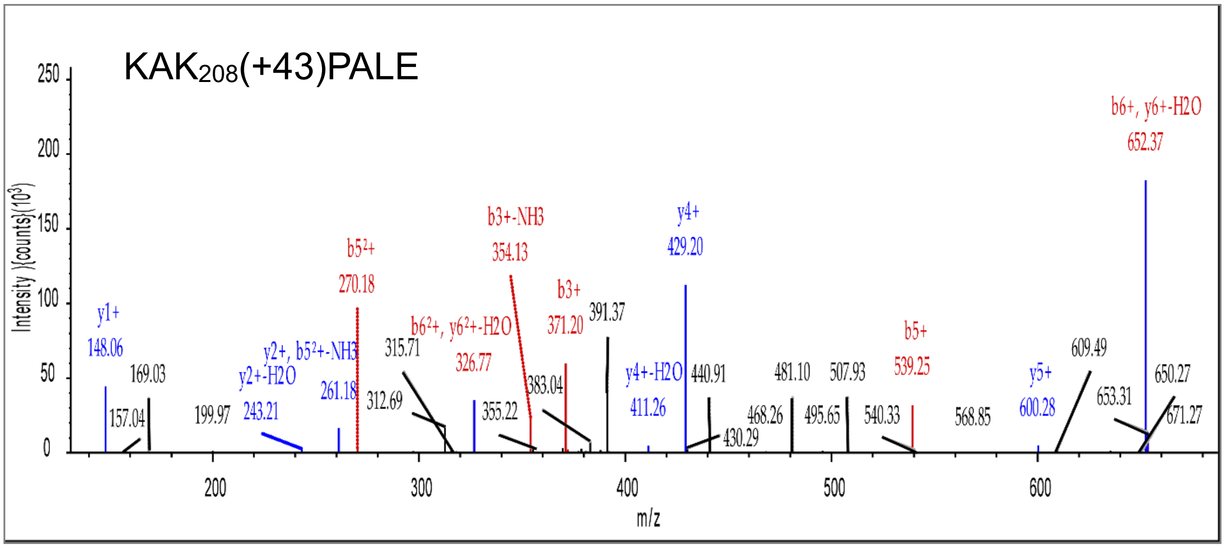

#1	b⁺	b²⁺	Seq.	y⁺	y²⁺	#2
1	129.10224	65.05476	K			7
2	200.13935	100.57331	A	671.37227	336.18977	6
3	371.24013	186.12370	K-Carbamyl	600.33515	300.67121	5
4	468.29289	234.65009	P	429.23438	215.12083	4
5	539.33001	270.16864	A	332.18161	166.59444	3
6	652.41407	326.71067	L	261.14450	131.07589	2
7			E	148.06043	74.53386	1

Sample:	Observed in:
Human plaque	yes
In-Vitro rHDL OCN	ND
In-Vitro rHDL MPO	ND
In-Vitro LP apoA-I OCN	ND
In-Vitro LP apoA-I MPO	ND

Figure S19: Annotated MS/MS spectrum and table of fragmentation ions for carbamylated apoA-I peptide KAK$_{208}$(carbamyl)PALE. Human apoA-I was either recovered via immunoprecipitation from homogenized human atherosclerotic aorta (Human plaque), or recombinant human apoA-I (lipid poor (LP) vs within rHDL) was exposed to the indicated *in vitro* carbamylation systems. ApoA-I was then subjected to proteolysis and proteomics analyses as described under Experimental Procedures. Top - CID spectrum for the indicated apoA-I peptide sequence harboring a carbamyllysine residue. Middle – Fragmentation table of peptide ions. Bottom – Table stating whether the carbamyllysine (K+43)-harboring apoA-I peptide was observed either in apoA-I recovered from human plaque or within the indicated *in vitro* carbamylated apoA-I samples.

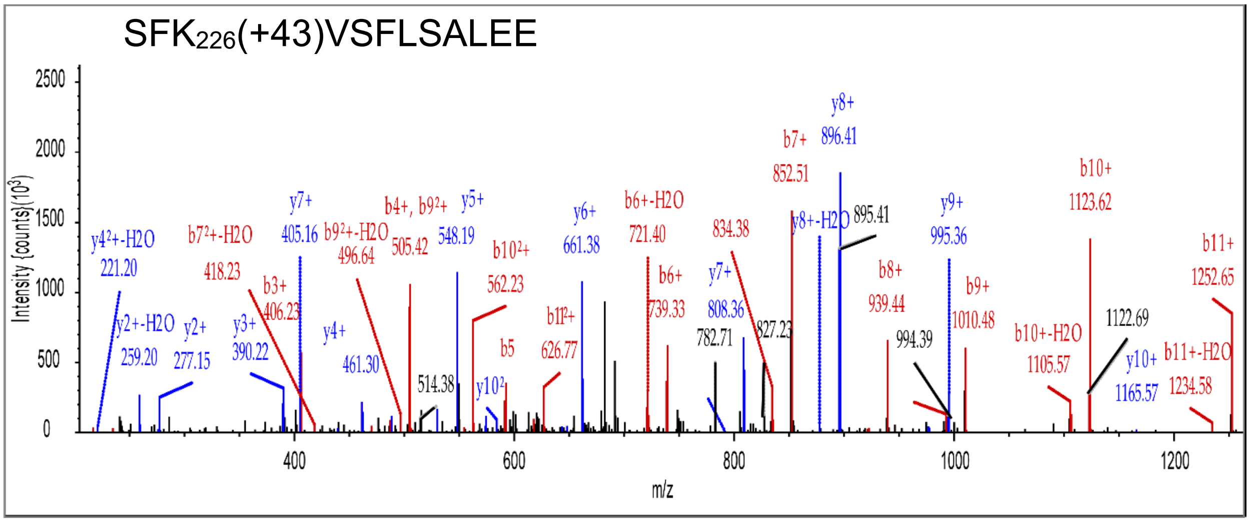

#1	b⁺	b²⁺	Seq.	y⁺	y²⁺	#2
1	88.03930	44.52329	S			12
2	235.10772	118.05750	F	1312.67834	656.84281	11
3	406.20850	203.60789	K-Carbamyl	1165.60993	583.30860	10
4	505.27691	253.14209	V	994.50915	497.75821	9
5	592.30894	296.65811	S	895.44074	448.22401	8
6	739.37735	370.19231	F	808.40871	404.70799	7
7	852.46142	426.73435	L	661.34030	331.17379	6
8	939.49344	470.25036	S	548.25623	274.63175	5
9	1010.53056	505.76892	A	461.22420	231.11574	4
10	1123.61462	562.31095	L	390.18709	195.59718	3
11	1252.65721	626.83225	E	277.10303	139.05515	2
12			E	148.06043	74.53386	1

Sample:	Observed in:
Human plaque	yes
In-Vitro rHDL OCN	yes
In-Vitro rHDL MPO	yes
In-Vitro LP apoA-I OCN	yes
In-Vitro LP apoA-I MPO	ND

Figure S20: Annotated MS/MS spectrum and table of fragmentation ions for carbamylated apoA-I peptide SFK₂₂₆(carbamyl)VSFLSALEE. Human apoA-I was either recovered via immunoprecipitation from homogenized human atherosclerotic aorta (Human plaque), or recombinant human apoA-I (lipid poor (LP) vs within rHDL) was exposed to the indicated *in vitro* carbamylation systems. ApoA-I was then subjected to proteolysis and proteomics analyses as described under Experimental Procedures. Top - CID spectrum for the indicated apoA-I peptide sequence harboring a carbamyllysine residue. Middle – Fragmentation table of peptide ions. Bottom – Table stating whether the carbamyllysine (K+43)-harboring apoA-I peptide was observed either in apoA-I recovered from human plaque or within the indicated *in vitro* carbamylated apoA-I samples.

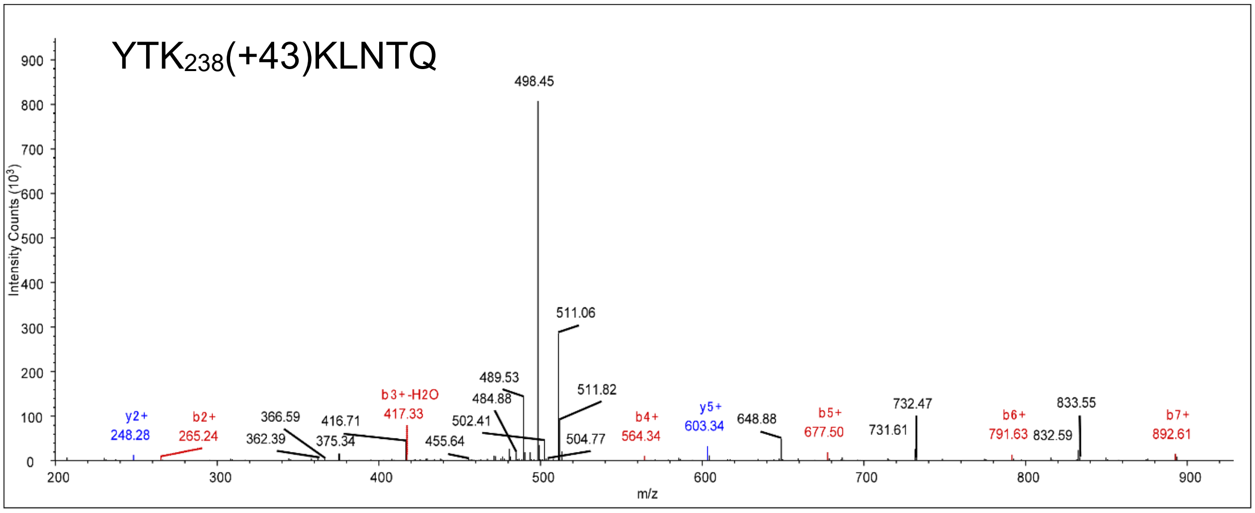

#1	b+	b2+	Seq.	y+	y2+	#2
1	164.07061	82.53894	Y			8
2	265.11828	133.06278	T	875.49451	438.25089	7
3	436.21906	218.61317	K-Carbamyl	774.44683	387.72705	6
4	564.31402	282.66065	K	603.34605	302.17666	5
5	677.39809	339.20268	L	475.25109	238.12918	4
6	791.44101	396.22415	N	362.16702	181.58715	3
7	892.48869	446.74798	T	248.12410	124.56569	2
8			Q	147.07642	74.04185	1

Sample:	Observed in:
Human plaque	yes
In-Vitro rHDL OCN	ND
In-Vitro rHDL MPO	yes
In-Vitro LP apoA-I OCN	ND
In-Vitro LP apoA-I MPO	ND

Figure S21: Annotated MS/MS spectrum and table of fragmentation ions for carbamylated apoA-I peptide YTK$_{238}$(carbamyl)KLNTQ. Human apoA-I was either recovered via immunoprecipitation from homogenized human atherosclerotic aorta (Human plaque), or recombinant human apoA-I (lipid poor (LP) vs within rHDL) was exposed to the indicated *in vitro* carbamylation systems. ApoA-I was then subjected to proteolysis and proteomics analyses as described under Experimental Procedures. Top - CID spectrum for the indicated apoA-I peptide sequence harboring a carbamyllysine residue. Middle – Fragmentation table of peptide ions. Bottom – Table stating whether the carbamyllysine (K+43)-harboring apoA-I peptide was observed either in apoA-I recovered from human plaque or within the indicated *in vitro* carbamylated apoA-I samples.

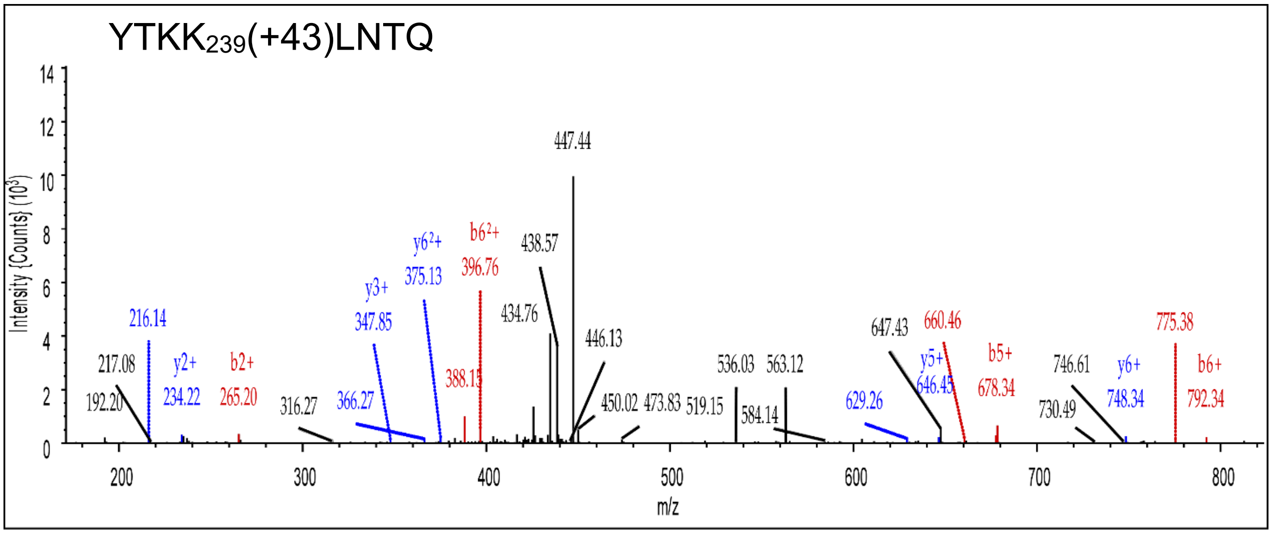

#1	b⁺	b²⁺	Seq.	y⁺	y²⁺	#2
1	164.07061	82.53894	Y			8
2	265.11828	133.06278	T	875.49451	438.25089	7
3	393.21325	197.11026	K	774.44683	387.72705	6
4	564.31402	282.66065	K-Carbamyl	646.35187	323.67957	5
5	677.39809	339.20268	L	475.25109	238.12918	4
6	791.44101	396.22415	N	362.16702	181.58715	3
7	892.48869	446.74798	T	248.12410	124.56569	2
8			Q	147.07642	74.04185	1

Sample:	Observed in:
Human plaque	yes
In-Vitro rHDL OCN	yes
In-Vitro rHDL MPO	ND
In-Vitro LP apoA-I OCN	ND
In-Vitro LP apoA-I MPO	ND

Figure S22: Annotated MS/MS spectrum and table of fragmentation ions for carbamylated apoA-I peptide YTKK$_{239}$ (carbamyl)LNTQ. Human apoA-I was either recovered via immunoprecipitation from homogenized human atherosclerotic aorta (Human plaque), or recombinant human apoA-I (lipid poor (LP) vs within rHDL) was exposed to the indicated *in vitro* carbamylation systems. ApoA-I was then subjected to proteolysis and proteomics analyses as described under Experimental Procedures. Top - CID spectrum for the indicated apoA-I peptide sequence harboring a carbamyllysine residue. Middle – Fragmentation table of peptide ions. Bottom – Table stating whether the carbamyllysine (K+43)-harboring apoA-I peptide was observed either in apoA-I recovered from human plaque or within the indicated *in vitro* carbamylated apoA-I samples.

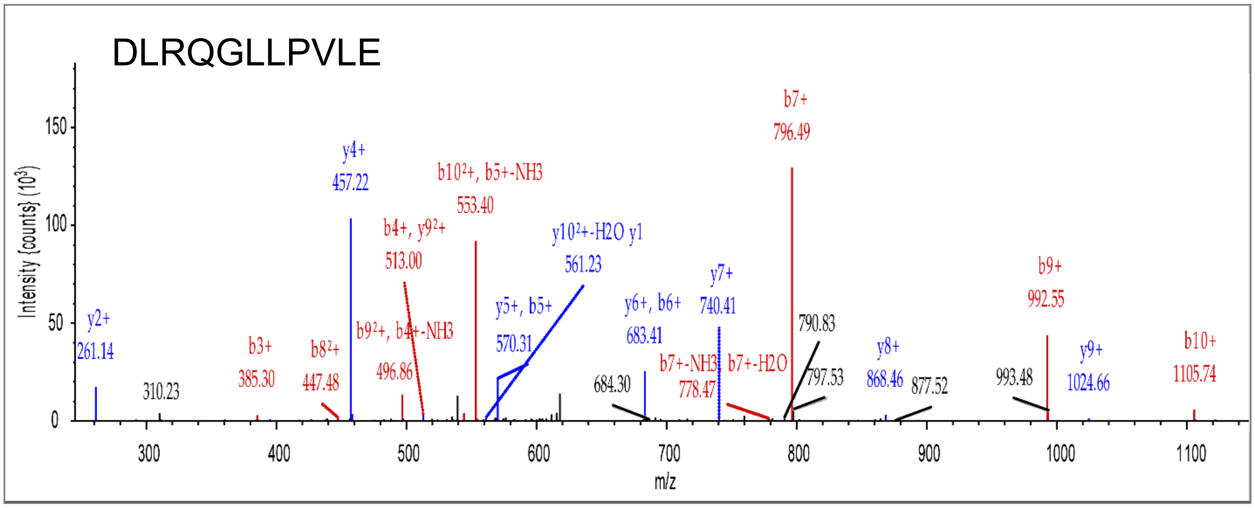

#1	b⁺	b²⁺	Seq.	y⁺	y²⁺	#2
1	116.03422	58.52075	D			11
2	229.11828	115.06278	L	1137.69902	569.35315	10
3	385.21939	193.11334	R	1024.61496	512.81112	9
4	513.27797	257.14262	Q	868.51384	434.76056	8
5	570.29944	285.65336	G	740.45527	370.73127	7
6	683.38350	342.19539	L	683.43380	342.22054	6
7	796.46756	398.73742	L	570.34974	285.67851	5
8	893.52033	447.26380	P	457.26568	229.13648	4
9	992.58874	496.79801	V	360.21291	180.61009	3
10	1105.67281	553.34004	L	261.14450	131.07589	2
11			E	148.06043	74.53386	1

Sample:	Observed in:
Human plaque	yes
In-Vitro rHDL OCN	yes
In-Vitro rHDL MPO	yes
In-Vitro LP apoA-I OCN	yes
In-Vitro LP apoA-I MPO	yes

Figure S23: Annotated MS/MS spectrum and table of fragmentation ions for apoA-I reference peptide DLRQGLLPVLE. Human apoA-I was either recovered via immunoprecipitation from homogenized human atherosclerotic aorta (Human plaque), or recombinant human apoA-I (lipid poor (LP) vs within rHDL) was exposed to the indicated *in vitro* carbamylation systems. ApoA-I was then subjected to proteolysis and proteomics analyses as described under Experimental Procedures. Top - CID spectrum for the indicated apoA-I reference peptide sequence. Middle – Fragmentation table of peptide ions. Bottom – Table stating whether the apoA-I reference peptide was observed either in apoA-I recovered from human plaque or within the indicated *in vitro* carbamylated apoA-I samples.

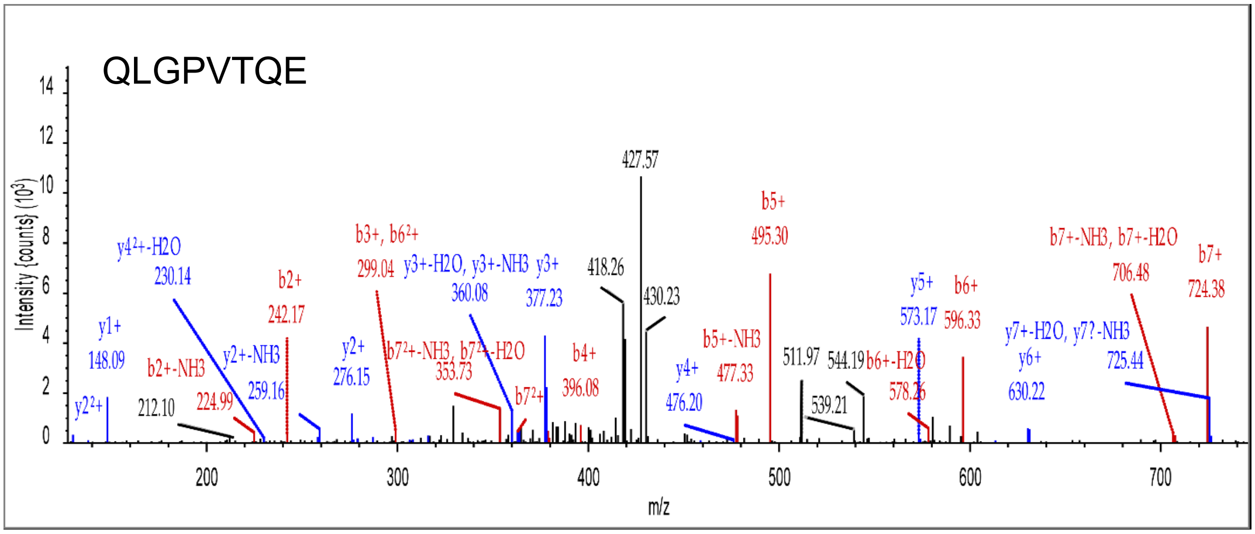

#1	b⁺	b²⁺	Seq.	y⁺	y²⁺	#2
1	129.06585	65.03657	Q			8
2	242.14992	121.57860	L	743.39340	372.20034	7
3	299.17138	150.08933	G	630.30933	315.65830	6
4	396.22415	198.61571	P	573.28787	287.14757	5
5	495.29256	248.14992	V	476.23510	238.62119	4
6	596.34024	298.67376	T	377.16669	189.08698	3
7	724.39882	362.70305	Q	276.11901	138.56314	2
8			E	148.06043	74.53386	1

Sample:	Observed in:
Human plaque	yes
In-Vitro rHDL OCN	ND
In-Vitro rHDL MPO	ND
In-Vitro LP apoA-I OCN	yes
In-Vitro LP apoA-I MPO	yes

Figure S24: Annotated MS/MS spectrum and table of fragmentation ions for apoA-I reference peptide QLGPVTQE. Human apoA-I was either recovered via immunoprecipitation from homogenized human atherosclerotic aorta (Human plaque), or recombinant human apoA-I (lipid poor (LP) vs within rHDL) was exposed to the indicated *in vitro* carbamylation systems. ApoA-I was then subjected to proteolysis and proteomics analyses as described under Experimental Procedures. Top - CID spectrum for the indicated apoA-I reference peptide sequence. Middle – Fragmentation table of peptide ions. Bottom – Table stating whether the apoA-I reference peptide was observed either in apoA-I recovered from human plaque or within the indicated *in vitro* carbamylated apoA-I samples.

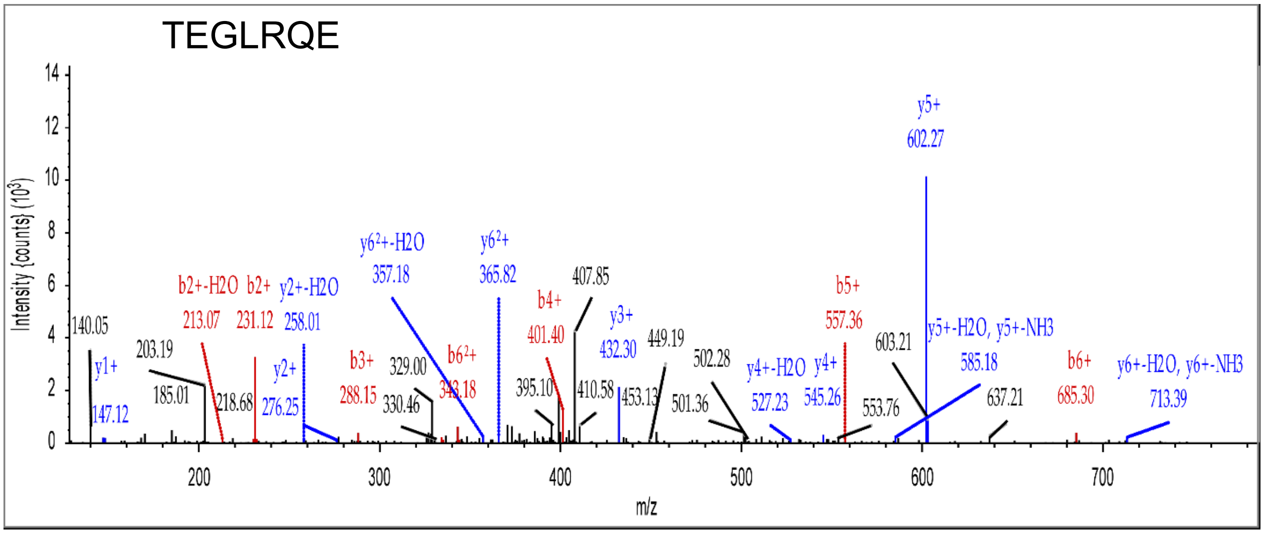

#1	b⁺	b²⁺	Seq.	y⁺	y²⁺	#2
1	102.05496	51.53112	T			7
2	231.09755	116.05241	E	731.36824	366.18776	6
3	288.11901	144.56314	G	602.32565	301.66646	5
4	401.20308	201.10518	L	545.30419	273.15573	4
5	557.30419	279.15573	R	432.22012	216.61370	3
6	685.36276	343.18502	Q	276.11901	138.56314	2
7			E	148.06043	74.53386	1

Sample:	Observed in:
Human plaque	ND
In-Vitro rHDL OCN	yes
In-Vitro rHDL MPO	yes
In-Vitro LP apoA-I OCN	ND
In-Vitro LP apoA-I MPO	ND

Figure S25: Annotated MS/MS spectrum and table of fragmentation ions for apoA-I reference peptide TEGLRQE. Human apoA-I was either recovered via immunoprecipitation from homogenized human atherosclerotic aorta (Human plaque), or recombinant human apoA-I (lipid poor (LP) vs within rHDL) was exposed to the indicated *in vitro* carbamylation systems. ApoA-I was then subjected to proteolysis and proteomics analyses as described under Experimental Procedures. Top - CID spectrum for the indicated apoA-I reference peptide sequence. Middle – Fragmentation table of peptide ions. Bottom – Table stating whether the apoA-I reference peptide was observed either in apoA-I recovered from human plaque or within the indicated *in vitro* carbamylated apoA-I samples.

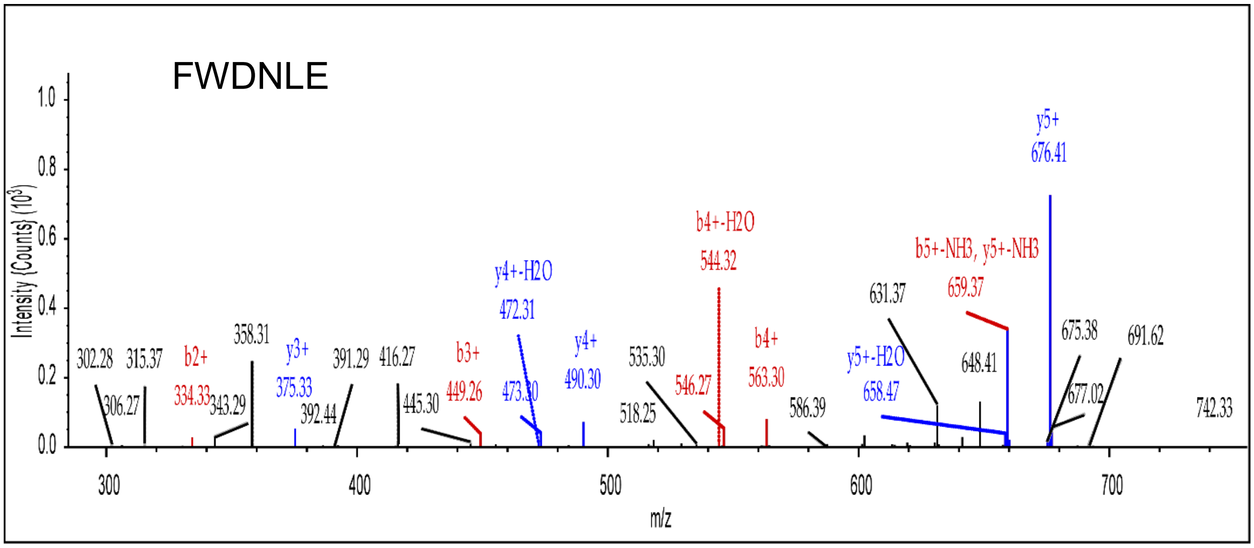

#1	b^+	Seq.	y^+	#2
1	148.07569	F		6
2	334.15500	W	676.29368	5
3	449.18195	D	490.21437	4
4	563.22487	N	375.18743	3
5	676.30894	L	261.14450	2
6		E	148.06043	1

Sample:	Observed in:
Human plaque	ND
In-Vitro rHDL OCN	ND
In-Vitro rHDL MPO	ND
In-Vitro LP apoA-I OCN	yes
In-Vitro LP apoA-I MPO	yes

Figure S26: Annotated MS/MS spectrum and table of fragmentation ions for apoA-I reference peptide FWDNLE. Human apoA-I was either recovered via immunoprecipitation from homogenized human atherosclerotic aorta (Human plaque), or recombinant human apoA-I (lipid poor (LP) vs within rHDL) was exposed to the indicated *in vitro* carbamylation systems. ApoA-I was then subjected to proteolysis and proteomics analyses as described under Experimental Procedures. Top - CID spectrum for the indicated apoA-I reference peptide sequence. Middle – Fragmentation table of peptide ions. Bottom – Table stating whether the apoA-I reference peptide was observed either in apoA-I recovered from human plaque or within the indicated *in vitro* carbamylated apoA-I samples.
